# Non-opioid analgesics for the prevention of chronic postsurgical pain: a systematic review and network meta-analysis

**DOI:** 10.1016/j.bja.2023.02.041

**Published:** 2023-04-12

**Authors:** Brett Doleman, Ole Mathiesen, Alex J. Sutton, Nicola J. Cooper, Jon N. Lund, John P. Williams

**Affiliations:** 1 Department of Anaesthesia and Surgery, Graduate Entry Medicine, University of Nottingham, Royal Derby Hospital, Nottingham, UK; 2 Department of Clinical Medicine, University of Copenhagen, Copenhagen, Denmark; 3 Department of Anaesthesia, Zealand University Hospital, Køge, Denmark; 4 Department of Health Sciences, University of Leicester, Leicester, UK

**Keywords:** chronic postsurgical pain, multimodal analgesia, network meta-analysis, non-opioid analgesia, systematic review

## Abstract

**Background:**

Chronic postsurgical pain is common after surgery. Identification of non-opioid analgesics with potential for preventing chronic postsurgical pain is important, although trials are often underpowered. Network meta-analysis offers an opportunity to improve power and to identify the most promising therapy for clinical use and future studies.

**Methods:**

We conducted a PRISMA-NMA-compliant systematic review and network meta-analysis of randomised controlled trials of non-opioid analgesics for chronic postsurgical pain. Outcomes included incidence and severity of chronic postsurgical pain, serious adverse events, and chronic opioid use.

**Results:**

We included 132 randomised controlled trials with 23 902 participants. In order of efficacy, i.v. lidocaine (odds ratio [OR] 0.32; 95% credible interval [CrI] 0.17–0.58), ketamine (OR 0.64; 95% CrI 0.44–0.92), gabapentinoids (OR 0.67; 95% CrI 0.47–0.92), and possibly dexmedetomidine (OR 0.36; 95% CrI 0.12–1.00) reduced the incidence of chronic postsurgical pain at ≤6 months. There was little available evidence for chronic postsurgical pain at >6 months, combinations agents, chronic opioid use, and serious adverse events. Variable baseline risk was identified as a potential violation to the network meta-analysis transitivity assumption, so results are reported from a fixed value of this, with analgesics more effective at higher baseline risk. The confidence in these findings was low because of problems with risk of bias and imprecision.

**Conclusions:**

Lidocaine (most effective), ketamine, and gabapentinoids could be effective in reducing chronic postsurgical pain ≤6 months although confidence is low. Moreover, variable baseline risk might violate transitivity in network meta-analysis of analgesics; this recommends use of our methods in future network meta-analyses.

**Systematic review protocol:**

PROSPERO CRD42021269642.

**Editor's key points**

• Chronic postsurgical pain is common after surgery, so identification of non-opioid analgesics with potential for preventing chronic postsurgical pain is important.
• A systematic review and network meta-analysis of randomised controlled trials of non-opioid analgesics was performed analysing effects on the incidence and severity of chronic postsurgical pain, serious adverse events, and chronic opioid use.
• The evidence suggests a possible reduction in chronic postsurgical pain up to 6 months by lidocaine (most effective), gabapentinoids, ketamine, and possibly dexmedetomidine.
• The evidence was insufficient for longer-term outcomes, opioid use, or serious adverse events.

Chronic postsurgical pain (CPSP) is common and may occur in 20%–30% of patients undergoing surgery,^1^ with associated severe consequences for patients.^2^ CPSP is ranked as the 11th highest research priority in all anaesthetic specialties from the James Lind Alliance.^3^ Non-opioid analgesia, the gold standard for acute pain management, might help reduce CPSP either via reductions in acute pain or specific molecular targets responsible for perioperative sensitisation.^4^

A recent review evaluated CPSP using a conventional meta-analysis and found possible benefit with gabapentinoids and lidocaine although clinical heterogeneity was observed, so analyses were reported separately based on time-point of outcome measurement, type of surgery, and degree of pain. This has major limitations on power and makes type II errors likely.^5^ Another recent network meta-analysis (NMA) focused on acute pain interventions only rather than CPSP outcomes.^6^

Network meta-analysis has a number of advantages over conventional meta-analyses such as improved power by utilising both direct and indirect estimates, the ability to compare agents not compared in the original trials, and the ability to rank treatments.^7^^,^^8^ However, transitivity is the major assumption of NMA. This is violated when potential effect modifiers are unevenly distributed between treatment comparisons, which leads to biased estimates of treatment effects. We previously demonstrated that baseline risk of pain without the intervention (proportion or average severity of pain in the control group) is an important effect modifier in acute pain trials (studies with higher pain without the intervention gain more clinical benefit with the intervention).^9^ Therefore, if baseline risk is an effect modifier, variable baseline risk between treatment comparisons can threaten the assumption of transitivity. It also has implications for clinical significance, as analgesics might be more effective in higher risk populations.

In an attempt to identify effective and optimal treatments for CPSP, this systematic review with NMA of non-opioid analgesics was adjusted for baseline risk with estimates reported from a fixed baseline value for fair comparison.

## Methods

We registered this review on the PROSPERO database before detailed study searches (CRD42021269642). Changes from the original protocol included expanding the assessment of transitivity to include mean age and sex of participants and time-point of outcome measurement and a *post hoc* analysis of drug administration timing and duration.^5^ This was done to ensure conduct of an NMA was valid before the analysis and help explain residual sources of heterogeneity. This review is reported in line with the PRISMA-NMA checklist (Supplementary data).^10^^,^^11^

### Search strategy and data extraction

We searched the MEDLINE, Embase, and CENTRAL databases for eligible published trials up to September 2021. The duration of the analysis period precluded repeating this.^12^ We searched for unpublished and ongoing trials on Clinicaltrials.gov, EU clinical trials register, and the International Clinical Trials Registry Platform (ICTRP). We searched the following conference proceedings (all years considered): American Society of Anesthesiologists (ASA) annual meetings, Euroanaesthesia, and the International Association of Pain's World Congress on Pain (IASP/WCP).^13^ Furthermore, we searched references and citations from included trials in Google Scholar and in other relevant systematic reviews.

Data were extracted onto an electronic database and included information such as author and year, type of surgery, analgesic and anaesthesia, number of participants randomised (and analysed), chronic opioid use, concurrent non-opioid/parenteral opioid analgesia, dose and route of administration of the intervention, comparator, definition of outcomes included, pain scores used, and information for risk of bias (ROB) assessments. Retracted papers were identified through the retraction watch website (http://retractiondatabase.org) and omitted.^14^^,^^15^ Authors were contacted by email for missing outcome data if needed.

### Inclusion criteria and outcomes

We included parallel-group, randomised controlled trials (RCTs). The population were adult participants (>16 yr of age) undergoing any surgical procedure, with no limitation on language or publication status. Interventions included non-opioid analgesics with known efficacy in reducing acute postoperative pain,^9^ which were delivered by any route except neuraxial administration, without restrictions on dose or duration if administered perioperatively. These included paracetamol, non-steroidal anti-inflammatory drugs (NSAIDs) and cyclooxygenase-2 (COX-2) inhibitors, ketamine, gabapentinoids (pregabalin and gabapentin), alpha-2 agonists (clonidine and dexmedetomidine), nefopam, i.v. lidocaine, and magnesium. To maximise statistical power, interventions with identical mechanisms of action were grouped together. We included all types of surgery requiring the presence of an anaesthetist. Two authors (BD and JPW) decided which trials to include with disagreements resolved by consensus.

We included both single and combination agents. We planned *a priori* to conduct separate analyses of single and combination agents. Comparators were placebo or other non-opioid analgesics. We only included pain outcomes beyond 3 months to comply with current definitions of CPSP.^1^ The primary outcome was the incidence of CPSP ≤6 months. Secondary outcomes included the incidence of CPSP >6 months, severity of CPSP ≤6 months and >6 months, incidence and severity of opioid use ≤6 months and >6 months, and serious adverse events (SAE).^16^

If trials reported multiple time-points for the same outcome, only the earliest was included. If SAEs were not explicitly reported, we attempted to extract these from other reported information if they were deemed to fit the definition, with the most serious included if multiple were reported. Pain severity was included from pain scores that could be converted to a 0–10 numeric rating scale. We attempted to extract the outcome closest to any pain at rest (including neuropathic pain). Neuropathic pain was later assessed on sensitivity analysis. As specific chronic pain scales are often ordinal and include other dimensions of pain, these were reported narratively (Supplementary data).

### Risk of bias

Risk of bias was undertaken using the Cochrane Risk of Bias (ROB) Tool (Version 1) by two study authors (BD and JPW) and disagreements were resolved by consensus. Attrition bias was rated as high risk when >10% of participants were lost to follow-up, as we deemed only a few events for rarer outcomes such as CPSP can have a relatively larger influence on study estimates. Trials were high risk for selective outcome reporting if they did not prespecify chronic pain in their protocol outcomes or did not fully report specified outcomes appearing in their methods. Other ROB was high if there was a clinically significant difference in baseline characteristics that may influence chronic pain.

We assigned trials an overall ROB and applied strict criteria to this overall assessment.^17^ Trials were only low ROB if they were low ROB for selection bias (randomisation and allocation concealment), blinding (outcome, participants, and investigators), and attrition bias (<10% loss to follow-up) with no high ROB elements. High ROB was assigned if any domain received high risk. Unclear ROB was anything in between these two assessments. These assessments informed both confidence in evidence and sensitivity analysis of low ROB trials only.^13^

### Confidence in estimates

The confidence in estimates was assessed using Confidence in Network Meta-Analysis (CINeMA).^18^ This framework takes into account factors such as within-study bias (from ROB assessment), reporting bias (from tests for small study effects), indirectness, imprecision, heterogeneity, and incoherence. Reporting bias was modified, as we assessed selective outcome reporting as a significant part of within-study bias (to avoid double counting). For tests for small study effects, we conducted a network meta-regression with inverse sample size as a covariate because of the correlation between standard errors with baseline risk and odds ratios (ORs).^19^^,^^20^ We compared fixed covariate estimates from the largest study with those from the unadjusted NMA estimates as per ROB-MEN.^21^

Imprecision takes account of confidence intervals and whether they cross the line of null effect (some concerns) or opposing clinical effects (major concerns). Heterogeneity was assessed on the basis of prediction intervals as per imprecision. For incoherence and heterogeneity, we only rated estimates as some concerns as we expected significant violations as a result of variations in baseline risk. We defined a minimal clinically important effect size as an OR of 0.80 for the incidence of pain, a mean difference (MD) of −1 for severity of pain,^22^ −10 mg oral daily morphine consumption, and an increase in SAEs of 1.05. Confidence in outcomes was downgraded owing to any concerns in the above domains from high to moderate, low, or very low. We downgraded twice if major concerns and once for some concerns with the exception of ROB, because of the strict criteria we described previously and the fact that most trials were only high risk on the basis of one domain (Supplementary data). We conducted these assessments in combination treatment analyses only in order to include all the available evidence. Of note, CINeMA was performed within a frequentist framework using netmeta in R (R Foundation for Statistical Computing, Vienna, Austria).

### Transitivity

The main assumption for performing an NMA is that of transitivity. This states that potential effect modifiers (factors which influence how effective the treatment is) are equally distributed between each treatment comparison in the network and that trials are jointly randomisable. Violation of this assumption causes issues when comparing the different treatments and negatively affects the validity of an NMA, although methods can be used to mitigate it.^23^

In order to test the transitivity assumption, we conducted a Bayesian random effects, network meta-regression on all included trials including potential trial-level effect modifiers as covariates. If multiple effect modifiers were identified and were unevenly distributed between treatment comparisons, we planned not to undertake an NMA. These covariates included mean age and proportion of female participants, baseline risk (control group proportion with pain for incidence or mean pain for severity),^9^ inclusion of participants on opioids, type of surgery (breast, thoracotomy, spinal, cardiac, and arthroplasty), use of regional anaesthetic techniques (for example, epidural or femoral nerve block), and time of outcome assessment. This was performed for the main nominal and ratio pain outcomes. Covariates were given a shared coefficient (because of the low number of trials for some interventions) with vague priors from the outcome scale determined heuristically. Markov chain Monte Carlo (MCMC) burn-in iterations were set at 5000 with 20 000 iterations used for inference. This analysis was conducted using the GeMTC online programme via the R package gemtc. Baseline risk was further analysed using WinBUGS, which takes into account the uncertainty of the estimate and its correlation to the treatment effect, thus excluding regression to the mean, in order to identify whether it remained a significant predictor.^24^

### Statistical analysis

Results from multidimensional chronic pain questionnaires were reported in a narrative review (Supplementary data). Where means and standard deviations (sd) were not reported, these were calculated from other reported statistics such as 95% confidence intervals, standard errors, medians, and inter-quartile range/range^25^ or estimated from other, similar study estimates (for sd). We created a network plot and network/treatment characteristic descriptive data to describe the geometry of the network for each outcome. In order to demonstrate the distribution of baseline risk between the different interventions, we constructed covariate plots of the baseline risk (*y*-axis) in each trial grouped by treatment (*x*-axis). This was performed in order to visualise the variable baseline risk and thus identify potential violations of transitivity (Supplementary data).

We used a random effects model for analysis and took account of correlation between effects from multi-arm trials. Because of the results of the transitivity assessment on network meta-regression, we report inferences from a fixed value of baseline risk in order to reduce the effects of variable baseline risk between comparisons, which could potentially violate the assumption of transitivity. Coefficients were treated as exchangeable. We selected a baseline risk value close to the mean to improve precision. We produced a regression plot to demonstrate the relationship between baseline risk (*x*-axis) and effect estimates (*y*-axis) for each intervention for all pain outcomes (Supplementary data). The Markov chain Monte Carlo simulation was performed using an adaptation of 10 000, a burn-in of 20 000 with 100 000 iterations used for inference (these were increased if convergence was an issue). Model fit was assessed by comparing the posterior mean of the residual deviance to the number of data points. Vague priors were used with a mean of 0 and an sd of 15*u* (where *u* is the largest maximum likelihood estimator in a trial). Convergence of the Markov chain Monte Carlo simulation was assessed using density plots, trace plots, and the Gelman-Rubin potential scale reduction factor (PSRF) with values <1.05 considered acceptable.

Inferences are presented as forest plots relative to placebo with effect estimates presented as posterior medians of OR or MD with 95% credible intervals (CrIs). For binary outcome data the link function was logit and likelihood distribution was assumed binomial, and for continuous data the link function was identity and likelihood distribution was assumed to be normal. Treatment ranking is presented as surface under the cumulative ranking (SUCRA) plots and tables. The SUCRA value from 0% to 100% gives the probability that the treatment is ranked top or one of the top ranks. We also present results in league heat plots which provide information on all comparisons within a specific outcome. All of these analyses are presented at a fixed baseline risk as described previously. We also assessed local inconsistency using the node-splitting method (reported when able to perform). As these analyses did not take account of baseline risk, we expected to observe inconsistency. Global inconsistency was assessed from the design-by-treatment interaction as part of CINeMA assessments. We conducted *a priori* sensitivity analyses with combination datasets including only low ROB trials and those that used neuropathic pain definitions for incidence. We also conducted a *post hoc* network meta-regression using timing of administration (preemptive or post-incision/mixed) and duration of intervention (>24 h). All analyses were conducted in gemtc^26^ and BUGSnet^27^ in R statistical software.

## Results

### Description of included trials

**Fig 1:**
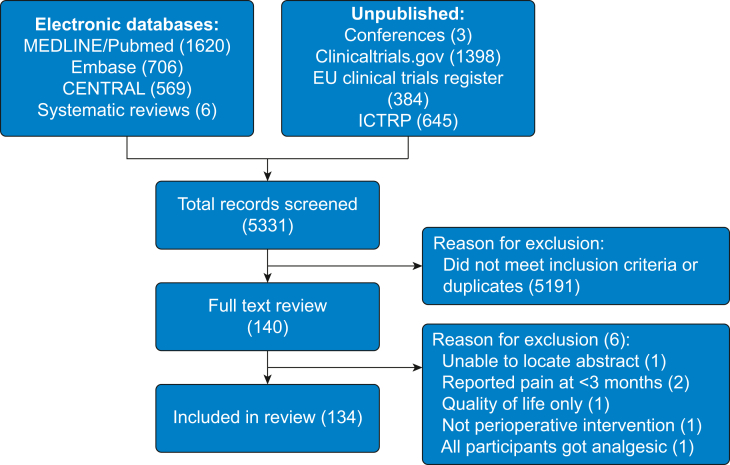
PRISMA flowchart of included trials. ICTRP, International Clinical Trials Registry Platform.

We included 134 trials (132 cohorts) with 23 902 participants from 5331 trials identified from all sources (Fig. 1). Most trials were performed with gabapentinoids (45), ketamine (33), and lidocaine (12). Details on specific agents, administration regimen, route, and dose can be found in the Supplementary data file (Table 3). Five trials were unpublished. There were few trials evaluating combination agents and few directly comparing different non-opioid analgesics. There were 93/132 (70%) RCTs that were high ROB, because of high attrition rates and possible selective outcome reporting. Types of surgery most commonly included were breast (26), thoracotomy (21), arthroplasty (16), spinal (14), and cardiac surgery (nine). Most trials used general anaesthesia (76) with 14 trials using an epidural for analgesia. For the primary outcome, 89% of interventions were administered before incision and 44% were continued beyond 24 h (Supplementary data).

### Transitivity

Only baseline risk was identified as a significant predictor for incidence (β −0.58; 95% CrI −1.00 to −0.12) and severity of pain (β −0.59; 95% CrI −0.87 to −0.29). They remained significant predictors when taking the uncertainty of the covariate into account. In addition, covariate plots identified varied distributions of baseline risk which could violate transitivity in conventional NMA.

### Incidence of chronic postsurgical pain ≤6 months

For single agents, this outcome included 88 trials with 8673 participants. There were 45 pairwise and 15 direct comparisons. The majority of trials were for gabapentinoids (33), ketamine (31), and lidocaine (12) with few trials for other interventions. Three interventions demonstrated estimates consistent with a reduction in the incidence of CPSP ≤6 months at a fixed covariate value of 0.35. The relative order of these based on SUCRA values were lidocaine (OR 0.32; 95% CrI 0.17–0.58), ketamine (OR 0.64; 95% CrI 0.44–0.92), then gabapentinoids (OR 0.67; 95% CrI 0.47–0.92). Dexmedetomidine was close to demonstrating an effect and was ranked second behind lidocaine (OR 0.36; 95% CrI 0.12–1.00).

**Fig 2:**
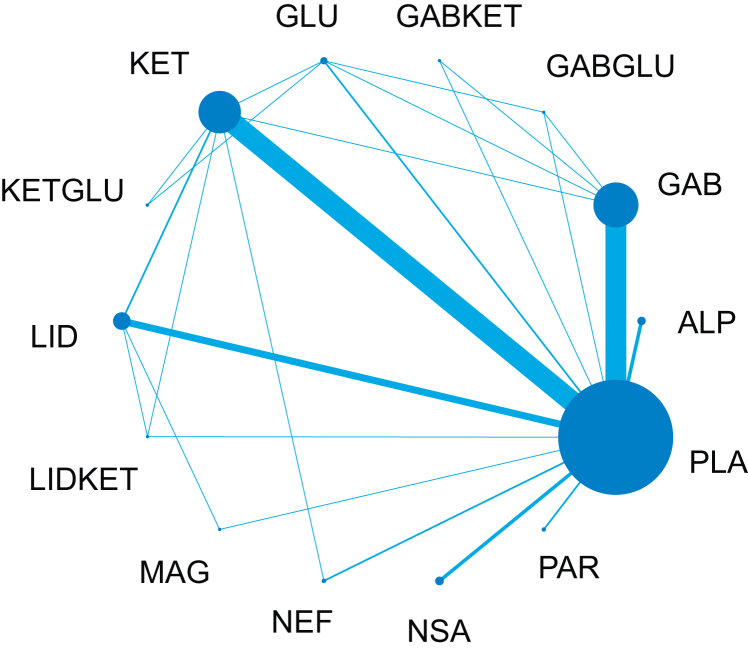
Network plot for the incidence of chronic postsurgical pain ≤ 6 months. Node size (blue circle) is proportional to the number of trials evaluating that intervention and the blue lines the number of comparisons between each treatment. ALP, alpha-2 agonists; GAB, gabapentinoids; GABGLU, gabapentinoids and glucocorticoids; GABKET, gabapentinoids and ketamine; GLU, glucocorticoids; KET, ketamine; KETGLU, ketamine and glucocorticoids; LID, lidocaine; LIDKET, lidocaine and ketamine; MAG, magnesium; NEF, nefopam; NSA, NSAIDs and COX-2 inhibitors; PAR, paracetamol; PLA, placebo.

**Fig 3:**
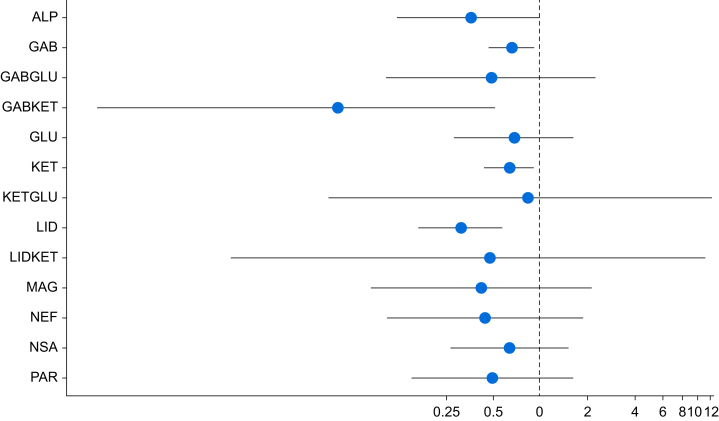
Forest plot showing the posterior median odds ratio with 95% CrIs at a fixed covariate value of 0.35 for the incidence of chronic postsurgical pain ≤6 months, which demonstrates possible reductions with ketamine, gabapentinoids and ketamine, gabapentinoids and lidocaine. X-axis is log scale to aid visualisation as a result of extreme values with effects relative to placebo. ALP, alpha-2 agonists; CrI, credible interval; GAB, gabapentinoids; GABGLU, gabapentinoids and glucocorticoids; GABKET, gabapentinoids and ketamine; GLU, glucocorticoids; KET, ketamine; KETGLU, ketamine and glucocorticoids; LID, lidocaine; LIDKET, lidocaine and ketamine; MAG, magnesium; NEF, nefopam; NSA, NSAIDs and COX-2 inhibitors; PAR, paracetamol.

For combination agents, this outcome included 88 trials with 8801 participants. There were 91 pairwise and 25 direct comparisons (Fig. 2). Five interventions showed estimates consistent with a reduction in CPSP ≤6 months at a fixed covariate value of 0.35 (Fig. 3). The relative order of these based on SUCRA values were gabapentinoids and ketamine (OR 0.05; 95% CrI 0.00–0.51), lidocaine (OR 0.32; 95% CrI 0.17–0.58), ketamine (OR 0.64; 95% CrI 0.44–0.92), and gabapentinoids (OR 0.67; 95% CrI 0.47–0.92). Dexmedetomidine was close to demonstrating an effect and was ranked third behind lidocaine (OR 0.36; 95% CrI 0.12–1.01).

There was no evidence of inconsistency on local tests although direct estimates were imprecise. On sensitivity analysis, reductions in neuropathic pain were seen with gabapentinoids and lidocaine. When including only low ROB trials, 16 trials remained with no single intervention estimates precise enough to demonstrate a reduction in CPSP. On CINeMA assessment, lidocaine, ketamine, and gabapentinoids *vs* placebo were low confidence. Nearly all other comparisons were very low confidence because of problems with ROB and imprecision. On *post hoc* network meta-regression, preemptive administration (β −0.02; 95% CrI −0.80 to 0.75) or duration of intervention >24 h (β −0.16; 95% CrI −0.65 to 0.31) did not predict efficacy of the interventions.

### Incidence of chronic postsurgical pain >6 months

For single agents, this outcome included 16 trials with 1613 participants. Three interventions made up the majority of trials including ketamine (six), gabapentinoids (five), and NSAIDs (four). One intervention demonstrated estimates consistent with a reduction in the incidence of CPSP >6 months (NSAIDs, OR 0.28; 95% CrI 0.08−0.83).

On sensitivity analysis, no intervention demonstrated any benefit. On CINeMA assessment, the evidence from NSAIDs compared with placebo was low confidence. All other comparisons were low to very low confidence.

### Severity of CPSP ≤6 months

For single agents, this outcome included 65 trials with 4996 participants. There were 36 pairwise and 14 direct comparisons. Most trials were for gabapentinoids (27) and ketamine (22). Three interventions showed estimates consistent with a reduction in pain at a fixed covariate value of 2 (mean pain score of 2 in control group). The relative order of these based on SUCRA values were lidocaine (MD −0.77; 95% CrI −1.95 to −0.09), gabapentinoids (MD −0.58; 95% CrI −0.80 to −0.36), then ketamine (MD −0.36; 95% CrI −0.62 to −0.1).

**Fig 4:**
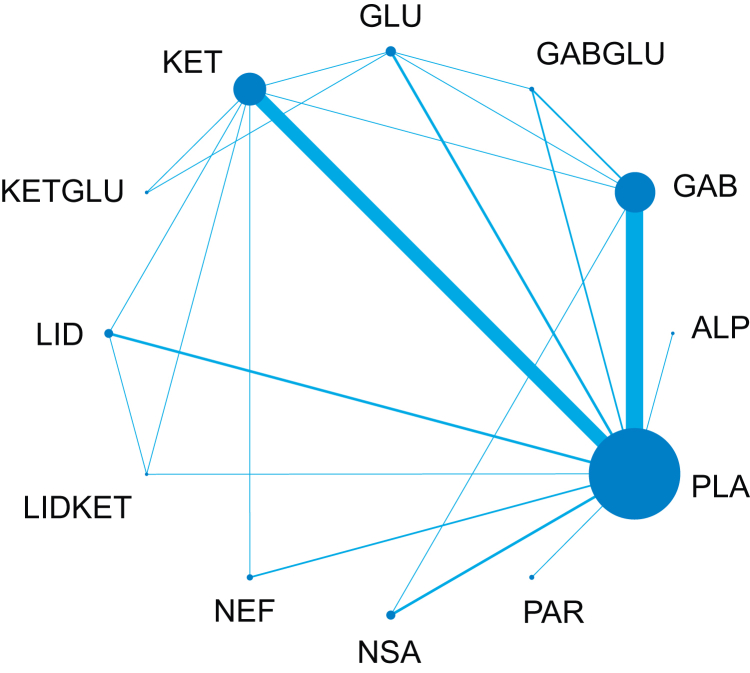
Network plot for the severity of chronic postsurgical pain ≤6 months. Node size (blue circle) is proportional to the number of trials evaluating that intervention and the blue lines the number of comparisons between each treatment. ALP, alpha-2 agonists; GAB, gabapentinoids; GABGLU, gabapentinoids and glucocorticoids; GLU, glucocorticoids; KET, ketamine; KETGLU, ketamine and glucocorticoids; LID, lidocaine; LIDKET, lidocaine and ketamine; NEF, nefopam; NSA, NSAIDs and COX-2 inhibitors; PAR, paracetamol; PLA, placebo.

**Fig 5:**
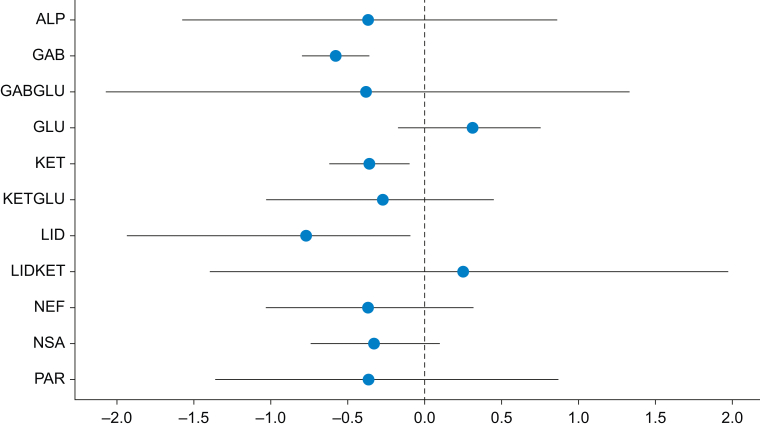
Forest plot showing the posterior median mean difference with 95% CrIs at a fixed covariate value of 2 for the severity of chronic postsurgical pain ≤6 months. X-axis is on a linear scale. Effects are relative to placebo. ALP, alpha-2 agonists; CrI, credible interval; GAB, gabapentinoids; GABGLU, gabapentinoids and glucocorticoids; GLU, glucocorticoids; KET, ketamine; KETGLU, ketamine and glucocorticoids; LID, lidocaine; LIDKET, lidocaine and ketamine; NEF, nefopam; NSA, NSAIDs and COX-2 inhibitors; PAR, paracetamol.

For combination agents, this outcome included 65 trials with 5088 participants. There were 66 pairwise and 22 direct comparisons (Fig. 4). Three interventions showed estimates consistent with a reduction in pain at a fixed covariate value of 2 (Fig. 5). The relative order of these based on SUCRA values were lidocaine (MD −0.77; 95% CrI −1.93 to −0.10), gabapentinoids (MD −0.58; 95% CrI −0.8 to −0.36), then ketamine (MD −0.36; 95% CrI −0.62 to −0.10).

Local inconsistency was evident in some comparisons as these analyses did not take account of baseline risk. On sensitivity analysis, including only low ROB trials, only gabapentinoids demonstrated estimates consistent with a reduction in pain (MD −0.63; 95% CrI −1.26 to −0.02). On CINeMA assessment, lidocaine, gabapentinoids, and ketamine compared with placebo were low confidence. All other comparisons were mainly low to very low.

### Severity of chronic postsurgical pain >6 months

There were no combination agents in this analysis so results are reported for single agents only. This outcome included 14 trials with 2273 participants. There were 15 pairwise and six direct comparisons. The interventions with the highest number of trials were gabapentinoids (four) and ketamine (four). None of the included trials had estimates consistent with a reduction in the severity of CPSP. Results were similar on sensitivity analysis of low ROB trials. On CINeMA assessment, most evidence was moderate to low confidence mainly as a result of issues with ROB.

### Serious adverse events

The types of included outcomes were heterogenous as few trials explicitly reported this outcome. Examples of the type of outcomes included were mortality, respiratory depression, delirium, local anaesthetic toxicity (lidocaine), and cardiovascular/renal dysfunction (NSAIDs) (Supplementary Data). This outcome included 38 trials with 12 367 participants. We excluded combination interventions as events were few and it caused problems with the model. There were 21 pairwise and seven direct comparisons. Most included trials were for gabapentinoids (14) and ketamine (10). No estimates were consistent with a difference in SAEs. Only one comparison could be assessed for inconsistency with no evidence found. Sensitivity analysis of low ROB trials showed similar estimates. On CINeMA assessment, most evidence was moderate to low confidence because of issues with imprecision and ROB.

### Incidence of opioid use ≤6 months

This outcome was only performed including combination agents, as only one event was contributed by one combination intervention. We had to remove one study containing zero events for lidocaine because of problems with convergence. This outcome included nine trials with 1284 participants. No interventions were associated with reductions in the incidence of opioid use ≤6 months. Three trials evaluating gabapentinoids were low ROB with no difference in results. On CINeMA assessment, most evidence was low confidence because of issues with ROB and imprecision.

### Incidence of opioid use >6 months

This outcome included three trials with 604 participants of gabapentinoids and ketamine compared with placebo. There was no difference in the incidence of opioid use at >6 months. Only one study was at low ROB. On CINeMA assessment, all evidence was low to very low confidence because of issues with ROB and imprecision.

### Severity of opioid use ≤6 months

This outcome included three trials with 199 participants of gabapentinoids and ketamine compared with placebo. There was no difference in the severity of opioid use at ≤6 months. No trials were at low ROB. On CINeMA assessment, all evidence was low confidence because of issues with ROB and imprecision.

### Severity of opioid use >6 months

Only one study was included for this outcome comparing ketamine with placebo so NMA could not be performed.

## Discussion

This review evaluated the available evidence from RCTs for non-opioid analgesics and their influence on CPSP, SAEs, and chronic opioid use. We found evidence of shorter-term reductions in CPSP for lidocaine, gabapentinoids, ketamine, and possibly dexmedetomidine. Lidocaine appeared to be the most effective. There was limited evidence for longer-term reductions in pain and opioid use at any time-point. Moreover, the confidence in the estimates was mainly low to very low. We found no difference between the interventions for SAEs, although this outcome demonstrated imprecision. Few trials evaluated combination agents with one study suggesting a large reduction in pain with a combination of pregabalin and ketamine.

The pathophysiology of CPSP is largely unknown,^28^ but includes level of acute pain,^1^ persistent peripheral inflammatory components, and central changes such as central sensitisation and hyperalgesia. The analgesics with the most preventive potential in this analysis, lidocaine,^29^ gabapentinoids,^30^ and ketamine,^31^ all have well-known anti-hyperalgesic properties. Although it remains possible that reductions in CPSP observed in this review are a consequence of reductions in acute pain.

A recent systematic review evaluated pharmacotherapy for CPSP and included 110 RCTs, but was limited in scope.^5^ Limitations of this review include reporting of multiple outcomes based on factors not found to be effect modifiers on our meta-regression analysis, less strict criteria for assessing attrition bias, use of *I*^2^ rather than prediction intervals for communicating statistical heterogeneity,^32^ lack of assessment of long-term opioid use or SAEs, no formal assessment of the certainty of evidence using GRADE,^13^ and no assessment of publication bias or assessment for imprecision. In terms of findings, some effects were observed for pregabalin, lidocaine, and NSAIDs, which were similar to some of our findings. However, our review found benefits with ketamine and dexmedetomidine on NMA. The previously published review^5^ reported intervention effect sizes without consideration of baseline risk despite vast ranges in underlying prevalence. As we have demonstrated, interventions can be more effective in higher baseline risk conditions. Therefore, if an intervention has been studied in certain lower baseline risk groups, effect estimates will be lower and might not be considered as clinically significant.^33^ For example, we found that for the primary outcome, lidocaine studies had a lower average baseline risk which could underestimate clinical significance for this agent. Moreover, the previous review^5^ further limited power by separating outcomes on the basis of time-point of outcome, drug duration, degree of pain, and type of surgery, whilst also separating interventions with identical pharmacological mechanisms. The major advantage of our NMA is improved power and the ability to take account of direct evidence where available. Also, by not separating outcomes in this way we further improved power. Although the trade-off of this could be increasing clinical heterogeneity, our adjustment for baseline risk and the lack of effect modification by time-point or type of surgery mitigates this substantially.

An NMA has been published evaluating interventions for preventing CPSP.^34^ Similar to our results, they found benefits with gabapentinoids and systemic local anaesthetics. The inclusion criteria for that NMA included neural blocks, opioids, and agents not consistently associated with reductions in acute pain (antidepressants). Despite the inclusion of more interventions, they included fewer trials than our analysis which could limit power. Moreover, the analysis did not consider baseline risk as a continuous predictor (a proxy of surgical subtypes was used). As we have demonstrated, variable baseline risk is a major threat to transitivity and we would argue such analyses do not allow valid comparisons between different agents. To illustrate with a clinical example, in order to assess the volatility of inhalation anaesthetics, we use saturated vapour pressure (SVP). However, because SVP (analogous to CPSP efficacy) varies with temperature (analogous to baseline risk in our NMA), if SVP values are taken from different agents at different temperatures, then sevoflurane could incorrectly appear more volatile than isoflurane. This is why SVP is taken at 20^o^C (analogous to fixed value of baseline risk in our analysis).

There are a number of limitations with our work. Firstly, the evidence was mainly low to very low confidence because of issues with residual bias, heterogeneity, and imprecision.^15^ Therefore, we would strongly caution against extrapolating too much from these preliminary findings because of the disagreement between meta-analyses and large RCTs.^17^ Changes in clinical practice are often informed after results of large RCTs, although our decision to include agents known to reduce acute pain means that some high-risk patients, often with overlapping risk factors for CPSP,^1^ could potentially derive some benefit on CPSP incidence if specific agents are used to reduce acute pain. Secondly, there was a large degree of clinical heterogeneity outside the influence of baseline risk, such as drug doses and regimens, which makes identifying optimum regimens problematic. However, our *post hoc* analysis demonstrated that timing of administration or duration of intervention were not predictive of efficacy, a finding supported by other studies.^34^ There may also be residual violations to transitivity outside of the assessed predictors in all our models. Thirdly, meta-regression analysis deals with study-level covariates and can be susceptible to ecology bias and confounding. Fourthly, there were few data from direct comparisons and combination therapies, and some included outcomes. Specific to SAEs, agents such as alpha-2 agonists (cardiac arrest),^35^ gabapentinoids (respiratory depression),^36^ and NSAIDs/COX-2 inhibitors (bleeding and myocardial events)^37^ have adverse effects which could preclude their use. Finally, some agents not included in our review have shown promise in reducing CPSP (such as duloxetine and venlafaxine),^34^ and therefore our review lacks comparative data for these agents. Their lack of inclusion was as a result of inclusion only of agents known to reduce acute postoperative pain consistently.

Despite this, our analysis provides future directions for clinical practice and research. With regards to clinical practice, our choice of including proven non-opioid analgesics was prespecified, as these agents are commonly used to reduce acute pain, opioid consumption, and opioid adverse events. Therefore, any clinical decisions on the potential benefits on CPSP are not taken in isolation, but with these other advantages in mind. As we have demonstrated, clinical significance can be improved if given to higher baseline risk groups, and with possible benefits of greater reductions in CPSP.

With regards to future research, because of the low confidence in our results, large adequately powered trials with low ROB methodology and more complete follow-up are required to substantiate these findings, especially for longer-term outcomes. With this in mind, the publication of ROCKet (ACTRN12617001619336: 4884 participants using ketamine), GAP (ISRCTN63614165: 1196 participants using gabapentin), PLAN (NCT04874038: 1150 participants using lidocaine), LOLIPOP (NCT05072314: 4300 participants using lidocaine), and CODEX (NCT04289142: 2400 participants using dexmedetomidine) will hopefully provide more definitive evidence on the subject. Although for some of these trials (GAP and CODEX), CPSP is a secondary outcome that might affect interpretation.^38^

In view of these ongoing trials and published reviews, it could be argued that our findings constitute research waste.^5^ However, we have highlighted the advantages of our review over those published previously. Furthermore, the evidence from our review might come many years before publication of these ongoing trials and potentially benefit current patients. It also improves external validity by including a range of surgeries and identifies baseline risk as a major determinant of efficacy, which could provide benefit in current clinical practice or future trial design. Moreover, baseline risk was identified as a major threat to transitivity in NMAs, which are often used to inform clinical practice, so use of these methods in future NMAs can help improve the accuracy of the evidence base and thus clinical practice.

In conclusion, current evidence suggests a possible reduction in chronic postsurgical pain with lidocaine (most effective), gabapentinoids, ketamine, and possibly dexmedetomidine up to 6 months. There was insufficient evidence for longer-term outcomes, opioid use, or serious adverse events.

## Authors’ contributions

Conception: BD, OM, AJS, JPW, JNL

Data analysis: BD, OM, AJS, JPW, NC

Writing manuscript, approving final version, and agreement to be accountable for data: all authors

## Declaration of interest

The authors declare that they have no conflicts of interest.

## Funding

NJC and AJS are supported by the National Institute for Health and Care Research (NIHR) Complex Reviews Support Unit (project number 14/178/29) and NIHR Applied Research Collaboration East Midlands (ARC EM). The views expressed are those of the authors and not necessarily those of the NIHR or the Department of Health and Social Care.
