# Supplementary material for: Non-opioid analgesics for the prevention of chronic postsurgical pain: a systematic review and network meta-analysis
Source: Br J Anaesth. 2023 Apr 12;130(6):719–28. doi: 10.1016/j.bja.2023.02.041 (PMC10251124; doi:10.1016/j.bja.2023.02.041)
Supplement: Multimedia component 1 [file mmc1.docx]

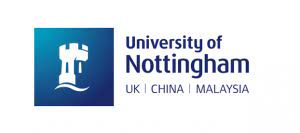

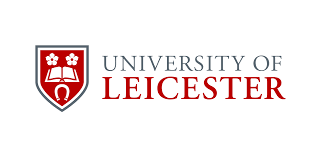


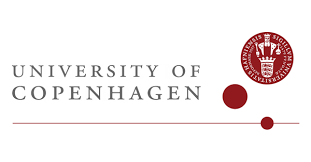


**NON-OPIOID ANALGESICS FOR THE PREVENTION OF CHRONIC POSTSURGICAL PAIN: A SYSTEMATIC REVIEW AND NETWORK META-ANALYSIS**

**Supplementary Data**

**Brett Doleman, Ole Mathiesen, Alex Sutton, Nicola Cooper, Jon Lund and John P Williams**

Table of Contents

[PRISMA Flowchart 3](#_Toc116897930)

[Search strategy for MEDLINE 4](#_Toc116897931)

[Transitivity assessment 5](#_Toc116897932)

[Characteristics of included studies 6](#_Toc116897933)

[Outcomes in included studies 25](#_Toc116897934)

[Risk of bias in included studies 41](#_Toc116897935)

[Incidence of CPSP ≤ 6 months (single agents) 59](#_Toc116897936)

[Incidence of CPSP ≤ 6 months (combination agents) 68](#_Toc116897937)

[CINeMA assessment for incidence of CPSP ≤ 6 months 80](#_Toc116897938)

[Incidence of CPSP > 6 months (single agents) 86](#_Toc116897939)

[CINeMA assessment for incidence of CPSP > 6 months 96](#_Toc116897940)

[Severity of CPSP ≤ 6 months (single agents) 99](#_Toc116897941)

[Severity of CPSP ≤ 6 months (combination agents) 108](#_Toc116897942)

[CINeMA assessment for severity of CPSP ≤ 6 months 120](#_Toc116897943)

[Severity of CPSP > 6 months (single agents) 125](#_Toc116897944)

[CINeMA assessment for severity of CPSP > 6 months 134](#_Toc116897945)

[Serious adverse events (single agents) 137](#_Toc116897946)

[CINeMA assessment for serious adverse events 145](#_Toc116897947)

[Incidence of opioid use ≤ 6 months (combination agents) 148](#_Toc116897948)

[CINeMA assessment for incidence of opioids ≤ 6 months 154](#_Toc116897949)

[Incidence of opioid use > 6 months (single agents) 157](#_Toc116897950)

[CINeMA assessment for incidence of opioids >6 months 163](#_Toc116897951)

[Severity of opioid use ≤ 6 months (single agents) 165](#_Toc116897952)

[CINeMA assessment for severity of opioid use ≤ 6 months 171](#_Toc116897953)

[Severity of opioid use > 6 months (single agents) 173](#_Toc116897954)

[R code used in analysis 174](#_Toc116897955)

[WinBUGS code for baseline risk analysis 182](#_Toc116897956)

[PRISMA-NMA Checklist 186](#_Toc116897957)

[Ongoing and unpublished clinical trials identified 189](#_Toc116897958)

[References for included studies 204](#_Toc116897959)

# **PRISMA Flowchart**

**
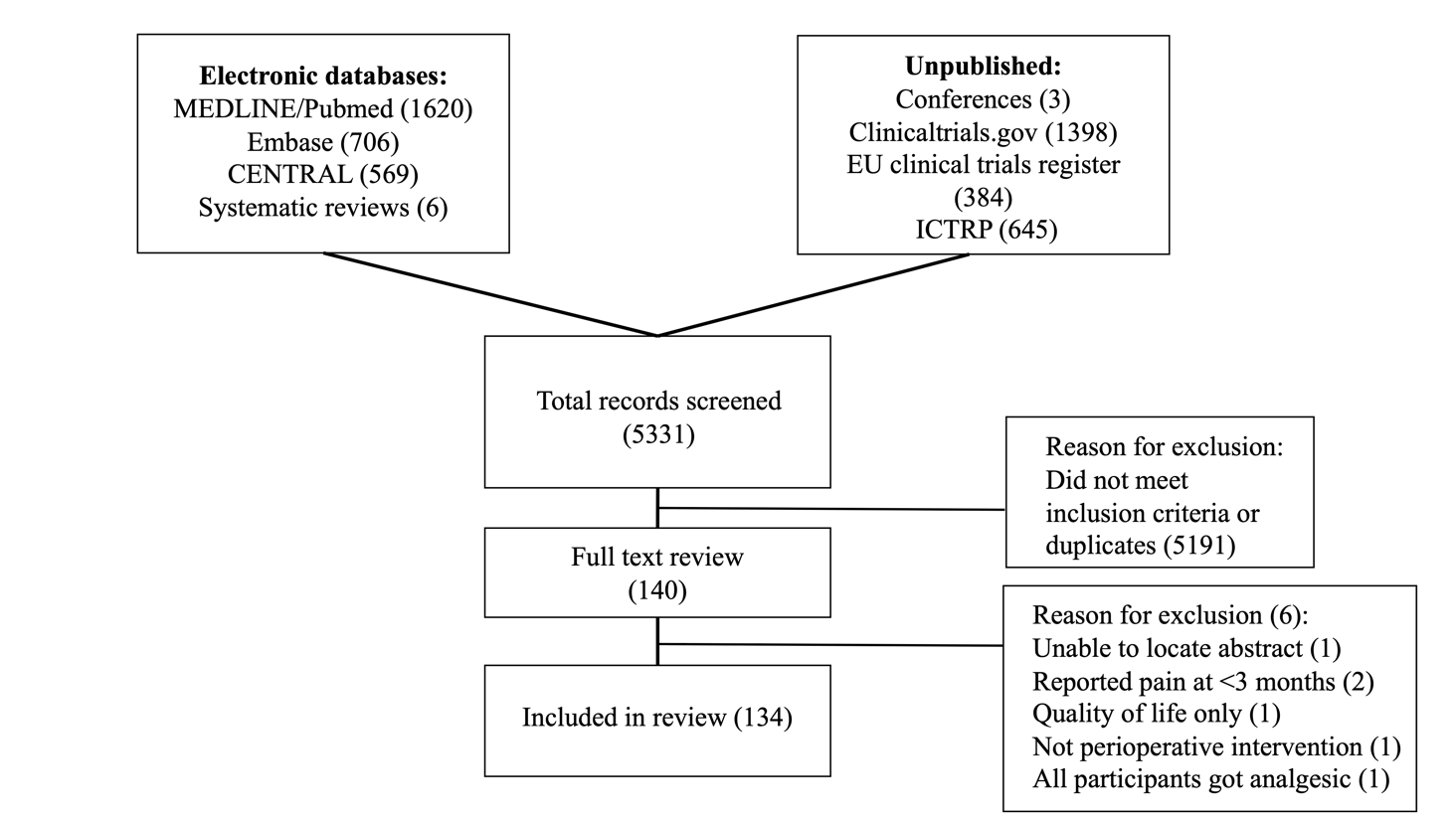
**

**Figure 1:** PRISMA flowchart showing the published and unpublished sources searched. Unpublished abstracts included in those under the section ‘Included in review’. Search conducted in September 2021.

**Search strategy for MEDLINE** (search adapted for other electronic databases)

**MEDLINE**

1. (postoperative).ti,ab
2. (postsurg*).ti,ab
3. (surgery)ti,ab
4. 1 OR 2 OR 3
5. (chronic pain).ti,ab
6. (chronic*).ti,ab
7. (persisten*).ti,ab
8. 5 OR 6 OR 7
9. 4 AND 8
10. (paracetamol).ti,ab
11. (acetaminophen).ti,ab
12. (NSAID* OR non-steroidal anti-inflammatory drug OR cyclooxygenase enzyme* OR cox OR ibuprofen OR ketoprofen OR diclofenac OR indomethacin OR ketorolac OR naproxen OR celecoxib OR parecoxib OR valdecoxib).ti,ab
13. (ketamine).ti,ab
14. (clonidine).ti,ab
15. (dexamethasone).ti,ab
16. (lidocaine).ti,ab
17. (magnesium).ti,ab
18. (dexmedetomidine).ti,ab
19. (gabapentin).ti,ab
20. (pregabalin).ti,ab
21. 10 OR 11 OR 12 OR 13 OR 14 OR 15 OR 16 OR 17 OR 18 OR 19 OR 20
22. (randomized controlled trial).pt
23. (controlled clinical trial).pt
24. (randomi?ed).ab
25. (placebo).ab
26. (drug therapy).fs
27. (randomly).ab
28. (trial).ab
29. (groups).ab
30. 22 OR 23 OR 24 OR 25 OR 26 OR 27 OR 28 OR 29
31. 9 AND 21 AND 30

# **Transitivity assessment**

**CPSP incidence ≤ 6 months**

| **Baseline risk** | **Age** | **Female (%)** | **Thoracotomy** | **Breast** | **Spinal surgery** |
| --- | --- | --- | --- | --- | --- |
| **-0.58 (-1.00 to -0.12)** | 0.06 (-0.38 to 0.52) | 0.59 (-1.22 to 2.44) | -0.26 (-0.81 to 0.25) | -0.07 (-0.6 to 0.45) | 0.74 (-0.22 to 1.72) |

| **Arthroplasty** | **Cardiac** | **Opioids** | **Anaesthesia** | **Time** |
| --- | --- | --- | --- | --- |
| -0.52 (-1.41 to 0.32) | -0.15 (-1.14 to 0.93) | 0.64 (-0.22 to 1.49) | -0.23 (-0.73 to 0.28) | 0.09 (-0.37 to 0.54) |

**Table 1:** CPSP ≤ 6 months incidence. β coefficients (median) for each study-level covariate with 95% credible intervals (CrIs) from Bayesian network meta-regression. Red values indicate covariates consistent with effect modifiers based on CrIs. See methods section for covariate details. Baseline risk remained a predictor when re-analysed taking the uncertainty of the covariate into account in WinBUGS: **-0.29 (95% CrIs -0.48 to -0.08)**. Note, this analysis used log odds for baseline risk rather than proportions.

**CPSP severity ≤ 6 months**

| **Baseline risk** | **Age** | **Female (%)** | **Thoracotomy** | **Breast** | **Spinal surgery** |
| --- | --- | --- | --- | --- | --- |
| **-0.59 (-0.87 to -0.29)** | -0.03 (-0.36 to 0.26) | -0.09 (-0.4 to 0.24) | 0.16 (-0.26 to 0.57) | -0.24 (-0.68 to 0.24) | -0.06 (-0.49 to 0.36) |

| **Arthroplasty** | **Cardiac** | **Opioids** | **Anaesthesia** | **Time** |
| --- | --- | --- | --- | --- |
| 0.25 (-0.28 to 0.74) | -0.11 (-0.61 to 0.38) | -0.11 (-0.91 to 0.67) | -0.22 (-0.51 to 0.1) | -0.04 (-0.41 to 0.33) |

**Table 2:** CPSP ≤ 6 months severity. β coefficients (median) for each study-level covariate with 95% credible intervals (CrIs) from Bayesian network meta-regression. Red values indicate covariates consistent with effect modifiers based on CrIs. See methods section for covariate details. Baseline risk remained a predictor when re-analysed taking the uncertainty of the covariate into account in WinBUGS: **-0.25 (95% CrIs -0.36 to -0.15)**.

# **Characteristics of included studies**

| **Author and year** | **Type of surgery** | **Anaesthesia** | **Number (analysed)** | **Concurrent non-opioid analgesia** | **Parenteral opioid analgesia** | **Intervention** | **Comparator** |
| --- | --- | --- | --- | --- | --- | --- | --- |
| ***Alpha-2 agonists (ALP)*** | | | | | | | |
| Alzeftawy 2015 | Breast | GA | 76 (73) | IM diclofenac 75mg OD | IV morphine PRN | IV dexmedetomidine 1mcg/kg STAT then 0.2mcg/kg/hr during surgery | Placebo (saline) |
| Bielka 2018 | Laparoscopic cholecystectomy | GA | 60 (60) | IV dexketoprofen 50mg STAT then ketoprofen 150mg/day and paracetamol 3g/day | SC morphine 5mg PRN | IV dexmedetomidine 0.5mcg/kg/hr intra-operatively | Placebo (saline) |
| Han 2019a | Hysterectomy | GA | 80 (69) | Not reported | IV tramadol 50mg PRN and sufentanil PCA | IV dexmedetomidine 0.5mcg/kg/hr intra-operatively | Placebo (saline) |
| Jain 2012 | Breast | GA | 86 (69) | IV lidocaine 1mg/kg STAT and IV/PO paracetamol 500-1000mg PRN | Not reported | IV dexmedetomidine 1mcg/kg STAT then 0.5mcg/kg/hr during surgery then 0.2mcg/kg/hr for 24 hours | Placebo (saline) |
| Mao 2020 | Thoracotomy | GA | 62 (56) | IV flurbiprofen 1mg/kg before and after surgery then PCA and IV dexamethasone 0.2mg/kg STAT | PCA sufentanil | IV dexmedetomidine 0.5mcg/kg STAT then 0.2-0.4mcg/kg/hr intra-operatively then 0.06mcg/kg/hr for 5 days | Placebo (saline) |
| ***Alpha-2 agonists and NSAIDs (ALPNSA)*** | | | | | | | |
| Sessler 2020 | Mixed | Not reported | 10010 (9963) | Not reported | Not reported | 1) PO aspirin 200mg STAT then 100mg OD for 7-30 days  2) PO clonidine 0.2mg then patch 0.2mg/day for 3 days 3) Combination of above | Placebo (tablets and patch) |
| ***Glucocorticoids (GLU)*** | | | | | | | |
| Bergeron 2009 | THA | SA | 50 (31) | PO paracetamol 650mg QDS and PO ibuprofen 400mg QDS | PCA morphine | IV dexamethasone 40mg | Placebo (saline) |
| Chan 2020 | TKA | SA and LA wound | 138 (137) | Wound ketorolac STAT, PO pregabalin 50mg NOCTE, paracetamol 1000mg QDS and celecoxib 200mg BD | PCA morphine | 1) IV dexamethasone 8mg 2) IV dexamethasone 16mg | Placebo (saline) |
| Jeyamohan 2015 | Spinal (cervical) | GA | 112 (93 at 3 months and 76 at 12 months) | Not reported | Not reported | IV dexamethasone 0.2mg/kg STAT then 4 postoperative doses of 0.06mg/kg | Placebo (saline) |
| Nielsen 2015 | Spinal (lumbar) | GA | 160 (112) | PO paracetamol 1000mg and ibuprofen 400mg STAT then QDS | IV sufentanil 0.2 mcg/kg and morphine PCA with boluses | IV dexamethasone 16mg | Placebo (saline) |
| Nielsen 2016 | Spinal (lumbar) | GA | 160 (99) | PO paracetamol 1000mg and ibuprofen 400mg STAT then QDS | IV sufentanil 0.2 mcg/kg and morphine PCA with boluses | IV dexamethasone 16mg | Placebo (saline) |
| Turan 2015 | Cardiac | GA | 1043 (1023) | Not reported | Not reported | IV methylprednisolone 500mg | Placebo |
| Weis 2006 | Cardiac | GA | 36 (28) | Not reported | Not reported | IV hydrocortisone 100mg STAT then 10mg/hour for 24 hours then reduced over 4 days | Placebo (saline) |
| ***Ketamine (KET)*** | | | | | | | |
| Bakr 2014 | Breast | GA | 40 (40) | Not reported | PCA morphine | IV ketamine 0.5mg/kg STAT then 0.25mg/kg/hr for 24 hours | Placebo (saline) |
| Bilgen 2012 | Caesarean section | GA | 140 (140) | IM diclofenac 75mg PRN | IV morphine 0.1mg/kg bolus then PCA morphine with boluses | 1) IV ketamine 0.25mg/kg 2) IV ketamine 0.5mg/kg  3) IV ketamine 1mg/kg | Placebo (saline) |
| Borys 2020 | Thoracotomy | GA | 80 (63) | IV metamizole 2000mg STAT then PRN | IV morphine 0.15mg/kg STAT then PRN then PCA morphine | IV ketamine 1mg/kg | No placebo |
| Brinck 2020 | Spinal (lumbar) | GA | 198 (182 at 3 months and 153 at 24 months) | IV paracetamol 1000mg STAT | IV fentanyl 50mcg and IV oxycodone boluses then PCA oxycodone | IV S-ketamine 0.5mg/kg STAT then:  1) 0.12mg/kg/hr  2) 0.6mg/kg/hr | Placebo (saline) |
| Cameron 2020 | Cardiac | GA | 82 (80) | IV paracetamol 1000mg QDS | IV fentanyl 25mcg PRN for 2 hours then SC hydromorphone 0.5-1mg PRN | IV ketamine 0.5mg/kg STAT then 0.5mg/kg/hr during surgery | Placebo (saline) |
| Chaparro 2010 | Breast | GA | 106 (50) | PO valdecoxib 40mg BD before surgery and IV lidocaine 1.5mg/kg at induction | Pethidine | IV ketamine 0.5mg/kg STAT then 0.2mg/kg/hr intra-operatively | Placebo (saline) |
| Chumbley 2019 | Thoracotomy | GA with epidural or PVB | 77 (67 at 3 months and 56 at 12 months) | Not reported | PCA (only some patients who didn't get regional analgesia) | IV ketamine 0.1mg/kg STAT then 0.1mg/kg/hr for 96 hours | Placebo (saline) |
| Crousier 2008 | Breast | GA | 36 (30) | PO paracetamol | IV morphine 1mg PRN | IV ketamine 0.5mg/kg STAT then 0.25mg/kg/hr intra-operatively | Placebo |
| Czarnetzki 2020 | Spinal (lumbar) | GA | 160 (141 at 6 months and 139 at 12 months) | IV ketorolac 30mg or ibuprofen 400mg PRN then after 48hrs, PO paracetamol 1000mg QDS and PO ibuprofen 400mg TDS | IV morphine 1-2mg boluses then PCA morphine | IV ketamine 0.25mg/kg STAT then 0.25mg/kg/hr then reduced to 0.1mg/kg/hr one hour before end of surgery until left recovery | Placebo (saline) |
| De Kock 2001 | Rectal cancer surgery | GA with epidural | 60 (56 at 6 months and 54 at 12 months) | Epidural clonidine 1mcg/kg STAT then infusion | Epidural sufentanil 2.5mcg STAT then infusion. PCA morphine postoperatively | IV ketamine:  1) 0.25mg/kg STAT then  0.125 mg/kg/hr  2) 0.5mg/kg STAT then 0.25mg/kg/hr intra-operatively | Placebo (saline) |
| Dualé 2009 | Thoracotomy | GA with wound/intra-pleural LA | 86 (69) | IV paracetamol 1000mg QDS and IV nefopam 80mg/day | IV morphine 5mg boluses then PCA morphine | IV ketamine 1mg/kg STAT then 1mg/kg/hr until skin closure then 1mg/kg over 24 hours | Placebo (saline) |
| Dullenkopf 2009 | Mixed (general and orthopaedic) | GA | 120 (80) | IV metamizole 1000mg STAT then QDS and IV paracetamol 1000mg PRN then PO QDS | IV morphine 0.03mg/kg PRN | 1) IV ketamine 0.15mg/kg 2) IV ketamine 0.5mg/kg | Placebo (saline) |
| Han 2019b (*unpublished*) | Breast | GA | 100 (86) | Flurbiprofen | Dezocine | IV ketamine 0.5mg/kg STAT then 0.25mg/kg/hr intra-operatively | Placebo (saline) |
| Hayes 2004 | Amputation | GA | 45 (32) | Not reported | PCA morphine or fentanyl | IV ketamine 0.5mg/kg STAT then 0.15mg/kg/hr for 3 days | Placebo (saline) |
| Hu 2014 | Thoracotomy | GA | 81 (78) | Not reported | PCA sufentanil | IV ketamine 1mg/kg STAT then 2mcg/kg/min for 72 hours | Placebo (saline) |
| Joseph 2012 | Thoracotomy | GA with epidural | 60 (37) | IV paracetamol 1000mg QDS and IV nefopam 20mg PRN or IV ketoprofen 50-100mg PRN | PCEA with sufentanil for 48 hours | IV ketamine 0.5mg/kg STAT then 3mcg/kg/min intra-operatively then 1.5mcg/kg/min for 48 hours | Placebo (saline) |
| Kang 2020 | Breast | GA | 184 (168) | PO pregabalin 150mg STAT then 75mg BD for 2 weeks | IV morphine 0.1mg/kg then morphine PCA with boluses | IV ketamine 0.5mg/kg STAT then 0.12mg/kg/hr intra-operatively | Placebo (saline) |
| Katz 2004 | Prostatectomy | GA | 168 (108) | Not reported | IV morphine boluses then PCA morphine | IV ketamine 0.2mg/kg STAT then 0.0025mg/kg/min (pre- or post-incision) | Placebo (saline) |
| Lee 2018 | Thyroidectomy | GA | 64 (49) | IV ketorolac 30mg PRN | IV pethidine 25mg STAT then IV fentanyl 50mcg or IV pethidine 25mg  PRN | IV ketamine 0.15mg/kg STAT then 2mcg/kg/min intra-operatively | Placebo (saline) |
| Lou 2017 | Breast | GA | 66 (63) | Not reported | PCA (opioid not reported) | IV ketamine 0.5mg/kg/hr for one hour daily for 7 days | Placebo (saline) |
| Malek 2006 | Breast | GA | 100 (86) | Not reported | IM pethidine 50-100mg PRN | IV ketamine 1mg/kg/day for 48 hours | Placebo (saline) |
| Mendola 2012 | Thoracotomy | GA with epidural | 66 (57) | IV paracetamol 45mg/kg/day and IV ketorolac 1.5mg/kg/day PRN then PO paracetamol and codeine on day 4 | Epidural sufentanil 0.4mcg/kg STAT then PCEA. IV morphine PRN | IV S-ketamine 0.1mg/kg/hr for 60 hours | Placebo (saline) |
| Nielsen 2017 | Spinal (lumbar) | GA | 150 (95) | PO paracetamol 1000mg before surgery then QDS | Participants usual opioids given before surgery and IV morphine 0.4mg/kg then IV sufentanil 5mcg if pain in recovery, then PCA morphine | IV S-ketamine 0.5mg/kg STAT then 0.25mg/kg/hr infusion | Placebo (saline) |
| Nielsen 2019 | Spinal (lumbar) | GA | 150 (98) | PO paracetamol 1000mg before surgery then QDS | Participants usual opioids given before surgery and IV morphine 0.4mg/kg then IV sufentanil 5mcg if pain in recovery, then PCA morphine | IV S-ketamine 0.5mg/kg STAT then 0.25mg/kg/hr infusion | Placebo (saline) |
| Perrin 2009 | TKA | GA and SA | 16 (12) | PO paracetamol 1500mg STAT then 750mg QDS with PO 800mg ibuprofen as rescue for severe pain | IT 100mcg morphine and PCA morphine with 2.5mg IV boluses | IV ketamine 0.5mg/kg STAT then 4mcg/kg/min intra-operatively | Placebo (saline) |
| Pehlivan 2019 | Thoracotomy | GA | 40 (40) | IV paracetamol 1000mg and IV dexketoprofen PRN | IV morphine 0.05mg/kg bolus then PCA morphine | IV ketamine 0.5mg/kg STAT then 2mcg/kg/min over 24 hours | Placebo (saline) |
| Peyton 2017 | Mixed (thoracic, abdominal, breast and inguinal hernia) | GA | 80 (80) | As per local clinical practice | Bolus and PCA (not specified) | IV ketamine 0.5mg/kg STAT then 0.25mg/kg/hr during surgery then 0.1mg/kg/hr for 24 hours | Placebo (saline) |
| Remerand 2009 | THA | GA | 160 (142) | IV paracetamol 1000mg QDS and ketoprofen 50mg QDS | IV morphine bolus then PCA morphine | IV ketamine 0.5mg/kg STAT then 2mcg/kg/min over 24 hours | Placebo (saline) |
| Spreng 2010 | Haemorrhoid-ectomy | GA with wound LA | 83 (77) | PO paracetamol 1000-2000mg, IV ketorolac 30mg and IV dexamethasone 8mg STAT then PO co-codamol 500/30mg. On discharge, paracetamol, diclofenac and co-codamol | IV fentanyl 0.05-0.1mg PRN | IV S-ketamine 0.35mg/kg STAT then 5mcg/kg/min intra-operatively | Placebo (saline) |
| Suppa 2012 | Caesarean section | SA | 56 (26) | PO paracetamol 1000mg QDS and PO ketorolac 30-90mg/day after 24 hours | IT sufentanil 5mcg and PCA morphine | IM S-ketamine 0.5mg/kg STAT then IV 2mcg/kg/min for 12 hours | Placebo (saline) |
| Suzuki 2006 | Thoracotomy | GA with epidural | 49 (44) | IV flurbiprofen 50mg PRN then PO loxoprofen 60mg or diclofenac 25mg | Epidural morphine | IV ketamine 0.05mg/kg/hr for 72 hours | Placebo (saline) |
| Sveticic 2008 | Mixed (orthopaedic) | GA and/or RA | 401 (352) | IV propacetamol 2000mg QDS and IV ketorolac 30mg PRN | PCA morphine | IV ketamine PCA | Placebo |
| Tena 2014 | Thoracotomy | GA with epidural | 125 (104) | IV paracetamol 1000mg QDS and metamizole 2000mg PRN | PCEA with fentanyl then SC methadone PRN | IV ketamine 0.5mg/kg STAT then 0.25mg/kg/hr for 48 hours | Placebo (saline) |
| ***Ketamine and Glucocorticoids (KETGLU)*** | | | | | | | |
| Galinski 2007 | Inguinal herniorrhaphy | GA with wound LA | 60 (50) | IV dexketoprofen 50mg and IV paracetamol 1000mg STAT then PO paracetamol 500mg QDS and PO ibuprofen 600mg TDS | IV tramadol 50mg PRN | 1) IV dexamethasone 8mg 2) IV ketamine 0.5mg/kg  3) Combination of above | Placebo (both active groups given saline placebo) |
| Shanthanna 2020 | VATS | GA with ICB (some PVB/epidural if open) | 27 (26) | IV ketorolac 15-30mg then NSAIDs and paracetamol | PCA (opioid not reported) | 1) IV ketamine 0.5mg/kg STAT then 0.1mg/kg/hr for 24 hours then PO memantine 5mg BD increasing over 4 weeks  2) IV dexamethasone 25mg STAT and postoperatively | Placebo (saline for both IVs and tablets) |
| Pavelescu 2020 (*unpublished*) | THA | GA | 58 | IV paracetamol 1000mg and ketoprofen 100mg PRN | Not reported | 1) IV dexamethasone 12mg STAT then 8mg postoperatively  2) IV ketamine 10mg STAT then 10mg/hr intra-operatively | Active groups only |
| ***Ketamine and Nefopam (KETNEF)*** | | | | | | | |
| Aveline 2014 | TKA | GA | 75 (69) | PO paracetamol 400mg QDS and PO ketoprofen 150mg BD | IV morphine 0.15mg/kg STAT then PCA morphine and boluses | 1) IV ketamine  2) IV nefopam: both 0.2mg/kg STAT then infusion 120mcg/kg/hr intra-operatively then 60mcg/kg/hr until day 2 | Placebo (saline) |
| ***Lidocaine (LID)*** | | | | | | | |
| Beaussier 2018 | Laparoscopic colectomy | GA | 63 (46) | IV paracetamol 1000mg QDS | IV morphine 2mg PRN then PCA morphine | IV lidocaine 1% 6ml STAT then 1.5% 4ml/hr for 48 hours | Placebo (saline) |
| Choi 2017 | Thyroidectomy | GA | 90 (84) | IV propacetamol 2000mg STAT then PO ibuprofen 200mg TDS | IV fentanyl 50mcg or tramadol 25mg PRN | IV lidocaine 2mg/kg STAT then 3mg/kg/hr intra-operatively | Placebo (saline) |
| Dai 2020 | Abdominal surgery (cancer resection) | GA | 120 (115) | Not reported | PCA sufentanil and IM tramadol 100mg PRN | IV lidocaine 1.5mg/kg/hr intra-operatively | Placebo (saline) |
| Galos 2020 | Breast | GA | 120 (96) | IV paracetamol 1g TDS | IV tramadol 50mg PRN | IV lidocaine 1.5mg/kg STAT then 2mg/kg/hr intra-operatively then 1mg/kg/hr for 24 hours | Placebo (saline) |
| Ghimire 2020 | Inguinal hernioplasty (laparoscopic) | GA with wound LA | 64 (60) | IV paracetamol 1000mg STAT and IV ketorolac 30mg TDS | IV morphine 1mg PRN then IV tramadol 50mg PRN | IV lidocaine 1.5mg/kg STAT then 2mg/kg/hr intra-operatively | Placebo (saline) |
| Grigoras 2012 | Breast | GA | 36 (36) | IV paracetamol 1000mg and IV diclofenac 75mg STAT then PO/PR diclofenac 50mg BD and PO/PR paracetamol 1000mg QDS | IV morphine PRN then PCA morphine with IM tramadol 100mg PRN | IV lidocaine 1.5mg/kg STAT then 1.5mg/kg/hr until 1 hour postoperatively | Placebo (saline) |
| Ibrahim 2018 | Spinal | GA | 44 (40) | IV ketorolac 60mg STAT then 30mg TDS and IV paracetamol 1000mg STAT then TDS then both PO for 2 weeks | IV morphine 0.1mg/kg PRN | IV lidocaine 2mg/kg STAT then 3mg/kg/hr intra-operatively | Placebo (saline) |
| Kendall 2018 | Breast | GA | 150 (121) | IV dexamethasone 4mg STAT then PO paracetamol and hydrocodone | IV hydromorphone 10mcg/kg STAT then IV 0.5mg PRN with PCA hydromorphone | IV lidocaine 1.5mg/kg STAT then 2mg/kg/hr until 1 hour postoperatively | Placebo (saline) |
| Martin 2008 | THA | GA | 60 (58) | PO paracetamol and NSAIDs after 48 hours | PCA morphine with 2-3mg boluses then SC morphine | IV lidocaine 1.5mg/kg STAT then 1.5mg/kg/hr until 1 hour postoperatively | Placebo (saline) |
| Terkawi 2015 | Breast | GA | 80 (61) | Not standardised | IV morphine 4mg or fentanyl 50mcg PRN | IV lidocaine 1.5mg/kg STAT then 2mg/kg/hr for 2 hours in PACU | Placebo (saline) |
| Toner 2021 | Breast | GA ± wound LA | 150 (149) | Not reported | Not reported | IV lidocaine 1.5mg/kg STAT then 2mg/kg/hr intra-operatively then SC 1.33mg/kg/hr for 12 hours | Placebo (saline) |
| Vasilev 2019 | Laparoscopic cholecystectomy | GA | 96 (92) | IV ketorolac 30mg PRN | IV trimeperidine 20mg PRN | IV lidocaine 1.5mg/kg STAT then 2mg/kg/hr intra-operatively | Placebo (saline) |
| ***Lidocaine and Alpha-2 agonists (LIDALP)*** | | | | | | | |
| Rekatsina 2019 (*unpublished*) | Hysterectomy | Not reported | 91 (74) | Not reported | Not reported | 1) IV dexmedetomidine 0.6mcg/kg STAT then 0.6mcg/kg/hr intra-operatively  2) IV lidocaine 1.5mg/kg STAT then 1.5mg/kg/hr intra-operatively | Placebo |
| ***Lidocaine and Pregabalin/Gabapentin (LIDGAB)*** | | | | | | | |
| Khan 2019 | Breast | GA ± wound LA | 100 (100) | Not standardised | Not standardised | 1) PO pregabalin 300mg STAT then 75mg BD for 9 days  2) IV lidocaine 1.5mg/kg STAT then 2mg/kg/hr intra-operatively  3) Combination of above | Placebo (tablets and infusion) |
| ***Lidocaine and Ketamine (LIDKET)*** | | | | | | | |
| Dhir 2020 | Open liver resection | GA and SA | 124 (113) | PO paracetamol 650mg QDS | IT morphine 300mcg then PCA hydromorphone | 1) IV lidocaine 0.33mg/kg/hr intra-operatively  2) IV ketamine 70mcg/kg/hr intra-operatively  3) Combination of above | Placebo (saline) |
| Jendoubi 2017 | Open nephrectomy | GA | 63 (60) | IV paracetamol 1000mg and IV nefopam 20mg STAT then QDS and TDS respectively | IV morphine 2mg PRN then PCA morphine | 1) IV lidocaine 1.5mg/kg STAT then 1mg/kg/hr for 24 hours  2) IV ketamine 0.15mg/kg STAT then 0.1mg/kg/hr for 24 hours | Placebo (saline) |
| ***Lidocaine and Magnesium (LIDMAG)*** | | | | | | | |
| Kim 2017 | Breast | GA | 126 (116) | IV propacetamol 20mg/kg and IV nefopam 0.4mg/kg STAT | Opioid not reported | 1) IV lidocaine 2mg/kg STAT then 2mg/kg/hr intra-operatively  2) IV magnesium 20mg/kg STAT then 20mg/kg/hr intra-operatively | Placebo (saline) |
| ***Nefopam (NEF)*** | | | | | | | |
| Kim 2018 | Thyroidectomy | GA | 58 (58) | IV ketorolac 15-30mg PRN | IV fentanyl 50mcg PRN | IV nefopam 0.2mg/kg STAT then 120mcg/kg/hr intra-operatively | Placebo (saline) |
| Na 2016 | Breast | GA | 94 (83) | IV ketorolac 30mg STAT then PRN and PO meloxicam 7.5mg | IV fentanyl 0.5mcg/kg PRN | IV nefopam 20mg | Placebo (saline) |
| ***NSAIDs/COX-2 inhibitors (NSA)*** | | | | | | | |
| Comez 2015 | Thoracotomy | GA with epidural | 40 (40) | Not reported | Epidural fentanyl 50mcg and morphine 3mg | IV dexketoprofen 50mg intra-operatively and one dose postoperatively | No mention |
| Haddad 2019 | Laparoscopic cholecystectomy | GA | 62 (49) | IV dexamethasone 8mg and IV ketamine 0.15mg/kg STAT then 1g paracetamol QDS and 20mg nefopam 6xday | IV morphine and tramadol PRN | IV parecoxib 40mg STAT | Placebo (saline) |
| Fransen 2006 | THA | GA, EA and SA | 902 (898) | Not reported | Not reported | PO ibuprofen 400mg TDS for 14 days | Placebo |
| Ge 2021 (*unpublished*) | Hepatectomy for carcinoma | GA with epidural | 105 (95) | Not reported | Epidural fentanyl | IV parecoxib 40mg STAT then BD for 3 days | Placebo (saline) |
| Lakdja 1997 | Breast | GA | 30 (28) | Not reported | PCA morphine | PO ibuprofen 400mg before and after surgery then TDS for 32 hours | Placebo |
| Ling 2016 | Thoracotomy | GA with epidural | 86 (76) | Not reported | Epidural morphine 2mg STAT then PCEA fentanyl and morphine | IV parecoxib 40mg STAT then BD for 60 hours | Placebo |
| Sun 2013 | Breast | GA | 60 (60) | Not reported | PCA fentanyl | IV flurbiprofen 50mg STAT then 6 hours later | Placebo (intralipid) |
| van Helmond 2016 | Breast | GA and PVB | 138 (94) | PO paracetamol 1000mg QDS | IV piritramide 3mg PRN | IV parecoxib 40mg STAT and after surgery then PO celecoxib 200mg BD for 5 days | Placebo |
| ***NSAIDs/COX-2 inhibitors and Glucocorticoids (NSAGLU)*** | | | | | | | |
| Romundstad 2006 | Breast | Sedation | 219 (175) | PO paracetamol 500mg PRN | None | 1) IV methylprednisolone 125mg STAT  2) IV parecoxib 40mg STAT | Placebo (saline) |
| ***Paracetamol (PAR)*** | | | | | | | |
| Koyuncu 2018 | Hysterectomy | GA | 140 (140) | PO diclofenac 75mg PRN | IV tramadol 0.5mg/kg STAT then PCA tramadol | IV paracetamol 1000mg QDS for 72 hours | Placebo (saline) |
| Turan 2017 | Cardiac | GA | 150 (105) | None permitted | PCA fentanyl or hydromorphone with boluses | IV paracetamol 1000mg QDS for 24 hours | Placebo (saline) |
| ***Paracetamol and Nefopam (PARNEF)*** | | | | | | | |
| Lee 2017 (*unpublished*) | Thyroidectomy | Not reported | 84 | Not reported | IV tramadol 50mg PRN | 1) IV propacetamol QDS for 24 hours  2) IV nefopam QDS for 24 hours  3) Combination of above | Active groups only |
| ***Pregabalin/Gabapentin (GAB)*** | | | | | | | |
| Acin 2009 | Inguinal, femoral or umbilical herniorrhaphy | Not reported | 140 (140) | Pyrazolone | Not reported | PO pregabalin 75mg NOCTE for 3 days before and 12 days after surgery | No placebo |
| Ahiskalioglu 2016 | Inguinal herniorrhaphy | SA | 60 | IV dexketoprofen 50mg BD | PCA fentanyl | PO pregabalin 150mg 1 hour before surgery | Placebo |
| Amr 2010 | Breast | GA | 100 (100) | PO co-codamol PRN from 24 hours | IV morphine PRN | PO gabapentin 300mg/day for 10 days | Placebo |
| Bouzia 2017 | Cardiac | GA | 101 (93) | IV paracetamol 1000mg QDS | IV morphine 5mg then PCA morphine | PO pregabalin:  1) 75mg  2) 150mg | Placebo |
| Buvanendran 2010 | TKA | CSE with wound LA | 240 (228) | PO celecoxib 400mg STAT then 200mg BD | IT fentanyl 25mcg then fentanyl PCEA | PO pregabalin 300mg STAT then reducing dose over 2 weeks | Placebo |
| Brogly 2008 | Thyroidectomy | GA with SCPB | 50 (47) | IV paracetamol 1000mg PRN and regional clonidine | IV tramadol 50mg PRN | PO gabapentin 1200mg 2 hours before surgery | Placebo |
| Brulotte 2015 | Thoracotomy | GA with epidural | 114 (99) | PR paracetamol 1300mg STAT then PO 650mg QDS | Epidural fentanyl and IV fentanyl 50-100mcg PRN and SC hydromorphone for shoulder pain | PO pregabalin 150mg BD for 10 doses, first dose 1 hour before surgery | Placebo |
| Burke 2010 | Spinal (lumbar) | GA with wound LA | 40 (38) | IV paracetamol 1000-2000mg and IV diclofenac 75mg STAT then PO paracetamol and diclofenac on ward | IV morphine 0.1mg/kg STAT and PRN. IM opioids on ward. | PO pregabalin 300mg 90 minutes before surgery then two doses of 150mg postoperatively | Placebo |
| Clarke 2009 | THA | SA | 126 (82) | PO paracetamol 1000mg QDS, PO celecoxib 400mg BD and IV dexamethasone 8mg STAT | IT fentanyl 10mcg then PCA morphine | PO gabapentin 600mg:  1) pre-operatively  2) postoperatively | Placebo |
| Clarke 2014 | TKA | SA and SNB/FNB | 212 (155) | PO celecoxib 400mg 2 hours before surgery then 200mg BD for 72 hours | IT fentanyl 10mcg then PCA morphine | PO gabapentin 600mg 2 hours before surgery then 200mg TDS for 4 days | Placebo |
| Clarke 2015 | THA | SA | 184 (130) | PO celecoxib 400mg STAT then 200mg BD | IT fentanyl 10mcg then PCA morphine | PO pregabalin 150mg then 75mg BD for 7 days | Placebo |
| Fassoulaki 2002 | Breast | GA | 50 (46) | IM paracetamol 600mg PRN then co-codamol | IM propoxyphene 75mg PRN | PO gabapentin 400mg TDS starting day before surgery for 10 days | Placebo |
| Fassoulaki 2012 | Hysterectomy | GA | 80 (62) | PO co-codamol PRN from day 3 | PCA morphine | PO pregabalin 150mg TDS day before surgery and for 5 days postoperatively | Placebo |
| Fawzi 2014 | Thoracotomy | GA | 60 (60) | PO paracetamol 1000mg QDS | IV morphine 0.1mg/kg PRN | PO pregabalin 75mg BD for 5 days before and after surgery | Placebo (Ferrex 50 plus) |
| Gaber 2019 | Thoracotomy | GA | 60 (60) | PCA ketorolac | IV morphine 0.1mg/kg STAT then PCA morphine | PO pregabalin 150mg 1 hour before surgery then BD for 5 days | Placebo (multi-vitamin) |
| Gianesello 2012 | Spinal (lumbar) | GA | 60 (60) | IV dexamethasone 8mg STAT and IV ketorolac infusion 2mg/hr | IV morphine 0.1mg/kg STAT then IV morphine infusion 0.01mg/kg/hr with boluses | PO pregabalin 300mg 1 hour before surgery then 150mg BD for 48 hours | Placebo |
| Grosen 2014 | Thoracotomy | GA with epidural | 104 (67) | PO paracetamol 2000mg STAT then 4000mg/day and ibuprofen 800mg/day | Epidural morphine and IV morphine 1-2mg PRN | PO gabapentin 1200mg 2 hours before surgery then increasing doses over 5 days | Placebo |
| Hah 2018 | Mixed (thoracic, breast and orthopaedic) | Mixed | 422 (410) | IV ketamine given to around 7% in each group | Not reported | PO gabapentin 1200mg then 600mg TDS for 72 hours | Active placebo (lorazepam) |
| Homma 2019 | Thoracotomy and VATS | GA with epidural | 100 (92) | PO loxoprofen 180mg/day or PO paracetamol 1200mg/day (if low eGFR) then PR diclofenac | Epidural fentanyl | PO pregabalin 50mg/day from day 2 for 3 months | No mention |
| Joshi 2013 | Cardiac | GA | 40 (40) | IV paracetamol 1000mg STAT then QDS and IV diclofenac 75mg PRN | IV tramadol 1mg/kg PRN | PO pregabalin 150mg 2 hours before induction then 75mg BD for 2 days | Placebo |
| Khurana 2014 | Spinal (lumbar) | GA | 90 (90) | IV diclofenac 75mg STAT | IV tramadol 1-2mg/kg PRN | 1) PO gabapentin 300mg  2) PO pregabalin 75mg 1 hour before surgery then TDS for 7 days | Placebo |
| Kim 2010 | Thyroidectomy | GA | 99 (94) | IV ketorolac 30mg STAT then PO ibuprofen 200mg BD | IV fentanyl 50mcg or IM tramadol 50mg PRN | PO pregabalin 150mg 1 hour before surgery and at 12 hours | Placebo |
| Kinney 2012 | Thoracotomy | GA with epidural | 146 (120) | IV ketorolac 15mg PRN and paracetamol 650mg QDS | Epidural hydromorphone and IV fentanyl PRN or PCA | PO gabapentin 600mg 2 hours before surgery | Active placebo (diphen-hydramine) |
| Konstantatos 2016 | VATS | GA with wound LA | 100 (100) | PO paracetamol 1000mg QDS | IV morphine 0.1-0.15mg/kg STAT then PCA morphine | PO pregabalin 300mg 30 minutes before surgery then 150mg BD for 5 days | Placebo |
| Koşucu 2014 | Thoracotomy | GA | 62 (60) | IV diclofenac 75mg STAT then PRN | IV morphine 4mg STAT then PCA morphine with boluses then pethidine 50mg PRN | PO gabapentin 1200mg 1 hour before surgery | Placebo |
| Macheridou 2012 (*unpublished*) | Breast | GA | 40 (38) | PO paracetamol PRN | IV tramadol PRN | PO pregabalin 75mg BD for 8 days | Placebo |
| Moore 2011 | Caesarean section | SA | 46 (36) | IV ketorolac 30mg and PR paracetamol 1000mg STAT then PO diclofenac 50mg TDS and PO paracetamol 1000mg QDS | IT fentanyl 10mcg and morphine 100mcg then SC morphine 2mg PRN | PO gabapentin 600mg 1 hour before surgery | Placebo |
| Myhre 2017 | Living donor nephrectomy | GA with wound LA | 80 (72 at 6 months and 70 at 12 months) | IV dexamethasone 8mg and IV paracetamol 1000mg STAT then QDS | IV fentanyl 50-100mcg STAT then PCA ketobemidone | PO pregabalin 150mg 1 hour before surgery then BD for 1 day | Placebo |
| Nikolajsen 2006 | Amputation | EA with GA/SA | 46 (34) | PO paracetamol 1000mg QDS | Not reported | PO gabapentin 300mg STAT then increasing doses over 30 days (up to 2400mg/day) | Placebo |
| Pesonen 2011 | Cardiac | GA | 70 (57) | IV paracetamol 1000mg STAT then TDS | IV oxycodone 0.05mg/kg PRN | PO pregabalin 150mg 1 hour before surgery then 75mg BD for 5 days | Placebo |
| Petersen 2018 | TKA | Not reported | 300 (215) | Not reported | Not reported | PO gabapentin:  1) 900mg 2 hours before surgery then 1300mg/day for 7 days  2) 600mg 2 hours before surgery then 900mg/day for 7 days | Placebo |
| Quail 2017 | Inguinal herniorrhaphy | GA with wound LA | 100 (41 at 6 months and 33 at 12 months) | IV lidocaine 1-1.5mg/kg and IV ketorolac 30mg STAT | IV fentanyl 0-500mcg PRN | PO gabapentin 300mg STAT then TDS for 2 days | Placebo |
| Reyad 2019 | Breast | GA | 200 (200) | IV paracetamol 1000mg and IV ketorolac 30mg STAT then ketorolac infusion. PO paracetamol/NSAIDs on discharge | IV morphine 0.1mg/kg STAT then morphine infusion with IV/IM morphine 3-5mg PRN | PO pregabalin 75mg 1 hour before induction then BD for 7 days | Placebo |
| Sadatsune 2016 | Carpal tunnel release | IVRA | 40 (37) | PO paracetamol 1000mg PRN | Not reported | PO gabapentin 600mg 1 hour before surgery | Placebo |
| Sen 2009a | Inguinal herniorrhaphy | SA | 60 (59) | IM diclofenac 75mg and PO paracetamol 500mg PRN | PCA tramadol | PO gabapentin 1200mg 1 hour before surgery | Placebo |
| Shimony 2016 | Craniotomy | GA or awake with wound LA | 100 (54) | IV paracetamol and metamizole 1000mg and IM diclofenac 75mg PRN | IV morphine 1mg and IV tramadol 100mg PRN | PO pregabalin 150mg NOCTE then STAT before surgery then BD for 72 hours | Placebo (starch) |
| Short 2012 | Caesarean section | SA | 132 (112) | IV ketorolac 30mg and PR paracetamol 1300mg STAT then PO diclofenac 50mg TDS and PO paracetamol 1000mg QDS | IT fentanyl 10mcg and morphine 100mcg then IV/SC morphine 2mg PRN | PO gabapentin 1 hour before surgery:  1) 300mg  2) 600mg | Placebo |
| Sidiropoulou 2016 | Thoracotomy | GA | 30 (30) | IV paracetamol 1000mg STAT then QDS then PO co-codamol from day 3 | PCA morphine | PO pregabalin 75mg BD starting the day before surgery for 5 days | Placebo (both tablet and saline infusion) |
| Singla 2015a | Inguinal herniorrhaphy | GA with wound LA | 425 (403) | PO naproxen 500mg BD and PO paracetamol 500-650mg PRN | Tramadol or oxycodone (unclear route) | PO pregabalin for 1 week:  1) 50mg/day  2) 150mg/day  3) 300mg/day | Placebo |
| Singla 2015b | TKA | SA, EA or CSE with peripheral nerve block | 307 | PO paracetamol 500mg and hydromorphone or paracetamol 500mg and oxycodone PRN | PCA/PCEA opioid (morphine, hydromorphone or fentanyl) | PO pregabalin for 6 weeks:  1) 150mg/day  2) 300mg/day | Placebo |
| Singla 2015c | Hysterectomy | GA | 501 | PO NSAIDs (naproxen, ibuprofen, diclofenac, ketorolac, or ketoprofen) or paracetamol | PCA morphine | PO pregabalin for 4 weeks:  1) 150mg/day  2) 300mg/day | Placebo |
| Ucak 2011 | Cardiac | GA | 40 (40) | After 24 hours, PO paracetamol 500mg TDS | IV tramadol 1mg/kg PRN | PO gabapentin 1200mg/day before and 2 days after surgery | Placebo |
| Vig 2019 | Breast | GA | 80 (71) | IV dexamethasone 8mg STAT and IV/PO paracetamol 15mg/kg STAT then QDS and IV/PO diclofenac 1.5mg/kg PRN | Not reported | PO pregabalin 75mg BD for 7 days starting before surgery | Placebo |
| YaDeau 2015 | TKA | CSE with FNB | 120 (93) | PO meloxicam 7.5-15mg and PO dexamethasone 6mg STAT then meloxicam and oxycodone and paracetamol | Epidural hydromorphone | PO pregabalin 0.5 hours before transfer to theatre then 1xBD for 14 days then NOCTE for 2 days:  1) 2x50mg  2) 2x100mg  3) 2x150mg | Placebo |
| Zarei 2016 | Spinal (lumbar) | GA with wound LA | 105 (105) | IV dexamethasone 16mg STAT | PCA morphine with boluses | PO pregabalin 300mg before surgery then:  1) 150mg BD for 2 doses  2) 150mg BD for 14 days | Placebo |
| ***Pregabalin/Gabapentin and Glucocorticoids (GABGLU)*** | | | | | | | |
| Choi 2013 | Spinal (lumbar) | GA | 120 (108) | IV ketorolac 30mg PRN | IV fentanyl 1mcg/kg STAT then IV infusion 0.4mcg/kg/hr for 48 hours | 1) PO pregabalin 150mg BD for 3 days  2) PO pregabalin 150mg BD for 3 days and IV dexamethasone 16mg | Placebo (saline and placebo tablets) |
| Momon 2019 | Spinal (lumbar) | GA with wound LA | 160 (127) | IV paracetamol 1000mg and IV ketoprofen 1mg/kg STAT then QDS | IV oxycodone 3mg PRN | 1) PO pregabalin 150mg 1 hour before surgery  2) IV dexamethasone 0.2mg/kg  3) Combination of above | Placebo (infusion and tablets) |
| ***Pregabalin/Gabapentin and Ketamine (GABKET)*** | | | | | | | |
| Anwar 2019 | Cardiac | GA | 150 (148) | PO paracetamol 1000mg QDS | PCA morphine | 1) PO pregabalin 150mg 2 hours before surgery and BD for 14 days with reducing dose  2) PO pregabalin 150mg 2 hours before surgery and for 14 days BD with reducing dose and IV ketamine 0.1mg/kg/hr at end of surgery for 48 hours | Placebo (lactose tablets and infusion) |
| Çelebi 2013 | Laparotomy | GA | 90 (90) | IM diclofenac BD | PCA fentanyl with boluses | 1) PO gabapentin 600mg 2 hours before surgery  2) IV ketamine 0.3mg/kg STAT then 0.2mg/kg at the end of surgery | Placebo infusion |
| Martinez 2014 | THA | GA | 142 (120) | Paracetamol and NSAIDs after 48 hours | IV morphine PRN then PCA | 1) PO pregabalin 150mg pre-operatively  2) IV ketamine 0.5mg/kg STAT then 3mcg/kg/hr intra-operatively  3) Combination of above | Placebo (saline and placebo tablets) |
| Sen 2009b | Hysterectomy | GA | 60 (60) | PO paracetamol 500mg PRN | IV morphine 4mg STAT then PCA morphine | 1) PO gabapentin 1200mg 2) IV ketamine 0.3mg/kg STAT then 0.05mg/kg/hr intra-operatively | Placebo (tablets and saline infusion) |
| ***Pregabalin/Gabapentin and NSAIDs (GABNSA)*** | | | | | | | |
| Matsutani 2015 | Thoracotomy | GA with epidural | 68 (68) | PR diclofenac 50mg PRN | Epidural fentanyl and PCA fentanyl and IM pentazocine | 1) PO pregabalin 75mg 2 hours before surgery then BD  2) PO loxoprofen 60mg 2 hours before surgery then TDS (both for 2 weeks duration) | No placebo |
| ***Pregabalin/Gabapentin and Paracetamol (GABPAR)*** | | | | | | | |
| Pavelescu 2009 (*unpublished*) | Thoracotomy | GA with epidural | 86 | Not reported | IV tramadol 100mg PRN | 1) PO pregabalin 150mg STAT then 150mg QDS for 72 hours  2) IV paracetamol 2000mg STAT then 1000mg QDS for 72 hours | Active groups only |

**Table 3:** Characteristics of included studies. Abbreviations: BD: twice daily, CSE: combined spinal and epidural, FNB: femoral nerve block, GA: general anaesthesia, ICB: inter-costal block, IM: intra-muscular, IT: intra-thecal, IV: intravenous, IVRA: intravenous regional anaesthesia, LA: local anaesthesia, NOCTE: at night, OD: once daily, PCA: patient-controlled analgesia, PCEA: patient-controlled epidural analgesia, PO: oral, PR: rectal, PRN: as required, PVB: paravertebral block, QDS: four times daily, RA: regional anaesthesia, SA: spinal anaesthesia, SCPB: superficial cervical plexus block, SNB: sciatic nerve block, STAT: bolus dose, TDS: three times daily.

# **Outcomes in included studies**

| **Author and year** | **Incidence of chronic pain** | **Chronic opioid use** | **Severity of chronic pain** | **SAEs** | **Notes** |
| --- | --- | --- | --- | --- | --- |
| ***Alpha-2 agonists (ALP)*** | | | | | |
| Alzeftawy 2015 | Chronic pain incidence (different characteristics used) | Not reported | 0-10cm VAS (values not reported) | Respiratory depression | 1) Troublesome pain used for incidence  2) SAE not included as no events (*author contacted but no reply)* |
| Bielka 2018 | Chronic pain incidence (other and pre-existing causes excluded) | Not reported | Not reported | Not reported | Not applicable |
| Han 2019a | Chronic and neuropathic pain incidence | Not reported | DN4 (used for neuropathic pain incidence) | Not reported | Chronic pain incidence included |
| Jain 2012 | Chronic pain incidence (regional pain) | No (analgesics only) | BPI (current pain 0-10) and SF-MPQ (total score lower in dexmedetomidine group: p<0.001) | Not reported | Not applicable |
| Mao 2020 | Chronic pain incidence | Not reported | Not reported | Death | Pain data is reported in discussion |
| ***Alpha-2 agonists and NSAIDs (ALPNSA)*** | | | | | |
| Sessler 2020 | Incisional pain | Not reported | BPI and NPI | Death or myocardial infarction | Can't extract separate data for groups causing unit of analysis issues so not included (*author contacted, reply received but no reply from co-investigator with data)* |
| ***Glucocorticoids (GLU)*** | | | | | |
| Bergeron 2009 | Not reported | Not reported | Harris hip score (no difference in pain at one year: p>0.05) | Not reported | Unable to include pain as scale up to 44 points so cannot be converted to a 0-10 scale |
| Chan 2020 | Not reported | Not reported | 0-10 NRS and WOMAC (significant decrease in linear trend with dexamethasone at 3 months only: p=0.02) | Death | 1) Dexamethasone groups combined  2) Median and IQR converted to mean (SD) |
| Jeyamohan 2015 | Not reported | Not reported | 0-100mm VAS (axial) and ODI (p=0.76 at 3 months and p=0.91 at 12 months) | Intubation for airway issues | SD estimated from other studies |
| Nielsen 2015 | Back pain | No (analgesics only) | 0-100mm VAS | New weakness or paralysis of legs | Not applicable |
| Nielsen 2016 | Back pain | No (analgesics only) | 0-100mm VAS and ODI (higher pain with dexamethasone at one year: p=0.01) | New weakness or paralysis of legs | Long term follow up of Nielsen 2015 |
| Turan 2015 | Incisional pain | No (analgesics only) | 0-10 VRS, mBPI and NPQ-SF only completed by those with pain: mBPI similar in both groups (p=0.93) | Composite of death, MI, stroke, AKI or respiratory failure | 1) SAEs taken from original publication  2) Average pain used for severity |
| Weis 2006 | Not reported | Not reported | SF-36 (lower pain in glucocorticoid group: p=0.01) | Not reported | Pain from SF-36 could not be included as ordinal scale in questionnaire so not comparable |
| ***Ketamine (KET)*** | | | | | |
| Bakr 2014 | Neuropathic pain (LANSS ≥ 12) | Not reported | LANSS (significantly lower in ketamine group at 3 and 6 months: both p<0.001) | Respiratory depression | SAE results not reported |
| Bilgen 2012 | Mild pain incidence | Not reported | VRS (not reported) | Not reported | Ketamine groups combined |
| Borys 2020 | Incidence of post-thoracotomy pain syndrome | Not reported | NPSI (no significant difference between groups) | Not reported | Not applicable |
| Brinck 2020 | Not reported | Not reported | 0-10 NRS | Cardiac arrest, pneumonia and delirium | 1) SAEs included at 48 hours only  2) Ketamine groups combined  3) Median and IQR converted to mean (SD) |
| Cameron 2020 | Pain at sternotomy site | Not reported | Not reported | Delirium | Chronic pain not included as no events in either group |
| Chaparro 2010 | Neuropathic pain (burning) | Not reported | Not reported | Not reported | Methodology from original paper |
| Chumbley 2019 | Any pain on movement | Yes (only incidence at 6 and 12 months can be extracted from included data) | 0-10 NRS on movement, LANSS (no difference at 3 months: p=0.42 or 12 months: p=0.72) and mBPI (no difference at 3 months: p=0.62 or 12 months: p=0.56) | Death | 1) Median and IQR converted to mean (SD) 2) 12 month data not included as no pain  3) Unable to include opioid consumption as N=5 |
| Crousier 2008 | Neuropathic pain | Not reported | VAS and QEDN score (no significant difference) | Not reported | Translated from French |
| Czarnetzki 2020 | Neuropathic pain | Yes (incidence of strong opioid use) | 0-10 NRS (worst pain in leg or back), DN4 (used to derive incidence), Roland Morris questionnaire (no difference in total score at 6 months: p=0.53 or 12 months: p=0.70) and COMI (no difference in total score at 6 months: p=0.95 or 12 months: p=0.84) | Not reported | Median and IQR converted to mean (SD) |
| De Kock 2001 | Residual pain in wound | Not reported (analgesics only) | Not reported | Death | Data extracted from graphs |
| Dualé 2009 | From 'ongoing pain' in NPSI | Opioid consumption in mg over 7 days | 0-100mm VAS and NPSI (no difference in total score: p=0.26) | Delirium or respiratory depression | 1) SAE not included as no events in either group  2) Median and IQR/range converted to mean (SD)  3) Opioid consumption / 7 to get daily values |
| Dullenkopf 2009 | Not reported | Not reported | 0-10cm VAS (rest) | Not reported | 1) Median and range (divide by 4) converted to mean (SD)  2) Ketamine groups combined |
| Han 2019b (*unpublished*) | Presence of persistent pain (any pain) | Not reported | 0-10 NRS and DN4 (both used to define incidence) | Delirium | Not applicable |
| Hayes 2004 | Phantom pain | Not reported (neuropathic agents only) | 0-10 NRS (highest score) | Death | Median and IQR converted to mean (SD) |
| Hu 2014 | Chronic pain incidence | Not reported (analgesics only) | 0-10 NRS | Death | Unclear in which group death occurred so not included |
| Joseph 2012 | Not reported | Not reported (analgesics only) | 0-10 NRS at rest | Respiratory depression | SAE not reported |
| Kang 2020 | Surgical site pain | Not reported | 0-10 NRS in those with pain and DN4 incidence (no difference: p=0.72) | Not reported | Median and IQR converted to mean (SD) |
| Katz 2004 | Surgical site pain | Not reported (analgesics only) | 0-10cm VAS (rest), FUPQ and MPQ (no inferential statistics reported although abstract states no difference) | Not reported | 1) Ketamine groups combined  2) SAEs difficult to attribute to study drugs  3) Pain severity only for those with pain |
| Lee 2018 | Chronic pain incidence | Not reported (analgesics only) | 0-10cm VAS (rest) | Respiratory depression | 1) No SAE events  2) Median and IQR converted to mean (SD) |
| Lou 2017 | Chronic pain incidence | Not reported | 0-10cm VAS (reported as 'no difference' p>0.05) | Not reported | 1) Article in Chinese  2) Data from abstract |
| Malek 2006 | Chronic pain incidence | Not reported | 0-10 NRS and MPQ | Not reported | Not applicable |
| Mendola 2012 | Chronic pain incidence (NRS ≥ 1) | Not reported (analgesics only) | 0-10 NRS (worst pain) and NPSI (used for incidence if > 0) | Respiratory failure and myocardial infarction | Pain severity extracted from graph |
| Nielsen 2017 | Not reported | Incidence and opioid consumption in mg/day | 0-100mm VAS (back pain), DN4 (no difference between groups: p=0.54), DaneSpine  questionnaire and Oswestry questionnaire (significant reduction in ketamine group: p=0.006) | Not reported | Median and IQR converted to mean (SD) |
| Nielsen 2019 | Not reported | Opioid consumption in mg/day | 0-100mm VAS (rest), DN4 (no significant difference: p=0.67), DaneSpine questionnaire and Oswestry questionnaire (significantly lower in ketamine group: p=0.04) | Not reported | 1) Median and IQR converted to mean (SD)  2) Unclear if daily analgesics were opioids for incidence  3) Long term follow up of Nielsen 2017 |
| Perrin 2009 | Incidence of mild to moderate pain | Not reported | WOMAC (pain subscale, no group result p value reported) | Not reported | Trial terminated early (N=12) |
| Pehlivan 2019 | Pain at surgical site | Not reported | Not reported | Delirium or respiratory depression | No SAE events |
| Peyton 2017 | Pain in area of surgery in last month | Not reported (analgesics only) | 0-100 NRS from mBPI and NPQ (used for incidence) | Death | 1) Only participants with pain included in severity  2) Median and IQR converted to mean (SD) |
| Remerand 2009 | Pain in operated hip | Yes | 0-100 NRS | Not reported | 1) Chronic opioid use not included as no events in either group  2) Data from 6 months included as only time-point reported |
| Spreng 2010 | Pain at surgical site (NRS ≥ 3) | Not reported (analgesics only) | 0-10 NRS (rest) | Not reported | Not applicable |
| Suppa 2012 | Pain around scar | Not reported (analgesics only) | Not reported | Respiratory depression, severe haemodynamic instability or severe neurological impairment | No SAE events |
| Suzuki 2006 | Chronic pain incidence (NRS ≥ 1) | Not reported (analgesics only) | 0-10 NRS (usual pain) | Pneumonia | 1) SAE from < 1 month  2) Median and IQR converted to mean (SD)  3) Severity data extracted from graph |
| Sveticic 2008 | Pain worse in the same location prior to surgery | Not reported | 0-10cm VAS (average) | Respiratory depression | Not applicable |
| Tena 2014 | Neuropathic pain (NPSI > 0) | Not reported | 0-10cm VAS (rest) and NPSI (used to define incidence of pain) | Death | Unclear which group death occurred in |
| ***Ketamine and Glucocorticoids (KETGLU)*** | | | | | |
| Galinski 2007 | Chronic pain incidence (rest) | Not reported | 0-10cm VAS (rest) | Respiratory depression | 1) SAE not reported  2) Median and IQR converted to mean (SD)  3) % used to calculate N in each group |
| Shanthanna 2020 | Chronic pain incidence (NRS > 3) | Yes (> 3 days/week) | 0-10 NRS, BPI and DN4 (used for incidence of neuropathic pain) | Death | 1) No data presented for each group (*author contacted, no reply*)  2) No SAE events |
| Pavelescu 2020 (*unpublished*) | Chronic pain incidence | Not reported | Not reported | Not reported | Unclear what data represents in graph (*author contacted, no reply)* |
| ***Ketamine and Nefopam (KETNEF)*** | | | | | |
| Aveline 2014 | Chronic pain incidence (VAS ≥ 40mm on movement at 12 months) and neuropathic pain incidence (DN4 ≥ 4 at 6 months) | Not reported (analgesics only) | 0-100mm VAS (rest) and DN4 (for neuropathic pain incidence) | Not reported | 1) Median and IQR converted to mean (SD)  2) Different outcomes for incidence due to reporting in manuscript 3) Methods from original study |
| ***Lidocaine (LID)*** | | | | | |
| Beaussier 2018 | Chronic pain incidence (at wound site) | Not reported | DN4 (used for incidence of neuropathic pain) | Local anaesthetic toxicity | Not applicable |
| Choi 2017 | Chronic pain incidence (area of surgery) | Not reported | SF-MPQ (no inferential statistics reported) | Local anaesthetic toxicity | No SAE events |
| Dai 2020 | Chronic pain incidence (surgery related pain) | Not reported (analgesics only although unclear time-point) | 0-10 NRS (average of rest and movement) | Arrhythmia | 1) No SAE events reported  2) Data extracted from graphs |
| Galos 2020 | Not reported | Not reported | MPQ (no difference between groups at one year: p=0.66) | Death | Not applicable |
| Ghimire 2020 | Chronic pain incidence (area of surgery) | Not reported | Not reported | Local anaesthetic toxicity | No SAE events |
| Grigoras 2012 | Chronic pain incidence (in last week) | Not reported (analgesics only although unclear time-point) | 0-100mm VAS as part of SF-MPQ (lower total pain index in lidocaine group: p=0.04) | Local anaesthetic toxicity | No SAE events |
| Ibrahim 2018 | Not reported | Not reported | 0-10cm VAS | Seizure | Not applicable |
| Kendall 2018 | Chronic pain incidence (area of surgery) | Not reported | BPI (no difference: p=0.10), SF-MPQ (no difference: p=0.45-0.91) and LANSS (no difference: p=0.75) | Not reported | 1) No SAE events described (unclear what reaction)  2) 6 month data used as much higher follow up rate to reduce attrition bias |
| Martin 2008 | Not reported | Not reported | 0-100mm VAS (rest) | Local anaesthetic toxicity | No SAE events |
| Terkawi 2015 | Chronic pain incidence | Yes (oral opioids) | 0-10 NRS (reported on ordinal scale) | Not reported | Not applicable |
| Toner 2021 | Chronic pain incidence (any pain at surgical wound) | Not reported | BPI and NPQ (not reported per group) | SAE (seizure, arrhythmia, medical emergency team activation, myocardial infarction, cerebrovascular accident, pulmonary embolism, deep vein thrombosis or unplanned re-operation) | Not applicable |
| Vasilev 2019 | Chronic pain incidence | Not reported | MPQ (unclear results for descriptive review) | Local anaesthetic toxicity | 1) Translated from Russian  2) No SAE events  3) Data entered assuming equal drop outs in each group using % |
| ***Lidocaine and Alpha-2 agonists (LIDALP)*** | | | | | |
| Rekatsina 2019 (*unpublished*) | Neuropathic pain (DN4) | Not reported | 0-10 NRS | Not reported | Conference poster, no data can be used (*author contacted and replied, awaiting publication of study)* |
| ***Lidocaine and Pregabalin/Gabapentin (LIDGAB)*** | | | | | |
| Khan 2019 | Neuropathic pain incidence (DN4) | Yes (mg of morphine consumed) | 0-10 NRS, SF-MPQ, DN4 and BPI | Local anaesthetic toxicity and respiratory depression | 1) Unable to use pain data as not reported in separate subgroups (*author contacted*)  2) No LA toxicity events, SAE is respiratory depression |
| ***Lidocaine and Ketamine (LIDKET)*** | | | | | |
| Dhir 2020 | Chronic pain incidence | Not reported | 0-10 NRS (rest) | Local anaesthetic toxicity (lidocaine) | Unable to include SAE as single symptoms |
| Jendoubi 2017 | Neuropathic pain incidence (DN4 ≥ 4) | Not reported | Not reported | Local anaesthetic toxicity (lidocaine) | No SAE events |
| ***Lidocaine and Magnesium (LIDMAG)*** | | | | | |
| Kim 2017 | Chronic pain incidence | Not reported | SF-MPQ (significant reduction in lidocaine versus control group: p=0.05) | Local anaesthetic toxicity (lidocaine) and prolonged muscle paralysis (magnesium) | No SAE events |
| ***Nefopam (NEF)*** | | | | | |
| Kim 2018 | Not reported (sensory disturbance only) | Not reported | 0-100 NRS (chest) | Not specified | 1) No SAE events in nefopam group  2) Median and IQR converted to mean (SD) |
| Na 2016 | Chronic pain incidence | Not reported | 0-10 NRS | Not reported | Not applicable |
| ***NSAIDs/COX-2 inhibitors (NSA)*** | | | | | |
| Comez 2015 | Chronic pain incidence (NRS ≥ 3) | Not reported | 0-10cm VAS | Not reported | Not applicable |
| Haddad 2019 | Chronic pain incidence (NRS ≥ 3) | Not reported (analgesics only) | Not reported | Cardiovascular events (chest pain) | Translated from French language |
| Fransen 2006 | Not reported | Not reported (analgesics only) | WOMAC 0-10 (included as scale changed to approximate to an NRS) | Serious adverse events (as per ICH definition) | Median follow up approximately 8 months so included in >6 months outcome |
| Ge 2021 (*unpublished*) | Chronic pain incidence (IASP definition) | Not reported | 0-10cm VAS (average pain) and SF-MPQ (no difference in affective: p=0.08 or sensory subscales: p=0.66) | Delirium | 1) Only those with pain included in pain severity  2) Median and IQR converted to mean (SD) |
| Lakdja 1997 | Chronic pain incidence (probably neuropathic) | Not reported | Not reported | Not reported | Translated from French language |
| Ling 2016 | Chronic pain incidence (incision) | Not reported | Not reported | Renal dysfunction | Table states chronic pain from 3 months so included as outcome |
| Sun 2013 | Chronic pain incidence | Not reported (analgesics only) | 0-10 NRS | Respiratory depression | 1) No SAE events  2) Median and IQR converted to mean (SD) |
| van Helmond 2016 | Chronic pain incidence (VAS > 30mm) | Not reported | 0-100mm VAS (rest) | Haematoma | 1) Chronic pain incidence not reported for each group so not included  2) Pain severity extracted from graph and 95% CI converted to SD |
| ***NSAIDs/COX-2 inhibitors and Glucocorticoids (NSAGLU)*** | | | | | |
| Romundstad 2006 | Chronic pain incidence (any pain at rest) | Not reported (analgesics only) | 0-10 NRS (reported on ordinal scale) | Not reported | 1) Methods from acute pain paper  2) Pain scores not reported as continuous data |
| ***Paracetamol (PAR)*** | | | | | |
| Koyuncu 2018 | Chronic pain incidence (incision) | Not reported (analgesics only) | 0-10cm VAS and DN4 (used for neuropathic pain incidence, significantly lower in paracetamol group: p=0.002) | Respiratory depression | 1) SAE not reported as dichotomous  2) Pain severity re-calculated from reported pain score categories |
| Turan 2017 | Chronic pain incidence (incision) | Not reported | 0-10 NRS, NPQ (used for incidence, only one event in placebo group) and BPI | Not reported | Not applicable |
| ***Paracetamol and Nefopam (PARNEF)*** | | | | | |
| Lee 2017 (*unpublished*) | Neuropathic pain | Not reported | 0-10 NRS (wound and posterior neck, reported as 'no significant difference') | Not reported | Unable to obtain author contact information |
| ***Pregabalin/Gabapentin (GAB)*** | | | | | |
| Acin 2009 | Not reported | Not reported | Not reported | Not reported | 1) Article in Spanish  2) Could not include pain as expressed as good to poor pain control |
| Ahiskalioglu 2016 | Chronic pain incidence (NRS > 3, not reported fully) | Not reported | 0-10 NRS | Not reported | *Author contacted for chronic pain data, no reply* |
| Amr 2010 | Neuropathic pain (stabbing/prickling pain) | Yes | 0-100mm VAS (movement) | Not reported | Pain intensity used from movement as only one reported |
| Bouzia 2017 | Chronic pain incidence | Not reported (analgesics only) | 0-10 NRS | Death, renal failure and delayed extubation | 1) Pregabalin groups combined  2) Chronic pain incidence not reported  3) Range converted to SD (divide by 4) |
| Buvanendran 2010 | Neuropathic pain (LANSS ≥ 12) | Yes | LANSS (used for incidence) | Clinically significant adverse events (not specified) | No SAE events |
| Brogly 2008 | Neuropathic pain (DN2 ≥ 3) | Not reported | DN2 (significantly lower in gabapentin group: p=0.03) | Not reported | Not applicable |
| Brulotte 2015 | Persistent post-thoracotomy pain (incision) | Not reported (analgesics only) | 0-10 NRS (average), BPI (only sleep significantly lower with pregabalin: p=0.03) and LANSS (used for incidence of neuropathic pain) | Infarction, renal failure, re-operation, stroke and pulmonary embolism | Median and IQR converted to mean (SD) |
| Burke 2010 | Not reported | Not reported | 0-100mm VAS (rest) and SF-MPQ (no difference at 3 months: p=0.08) | Not reported | Not applicable |
| Clarke 2009 | Chronic pain incidence | Not reported | 0-10 NRS (usual pain), hip arthroplasty pain questionnaire and NPS (inferential statistics not reported) | Not reported | 1) Pain intensity from only participants with pain  2) Gabapentin groups combined |
| Clarke 2014 | Not reported | Not reported | 0-10 NRS (during 6MWT) and WOMAC (0-20 for pain, no significant difference in pain: p=0.13) | Not reported | Not applicable |
| Clarke 2015 | Not reported | Not reported | 0-10 NRS (during 6MWT) and WOMAC (0-20 for pain, median of 1 in both groups) | Femoral fracture | 1) Pain during walking used in analysis  2) Median and IQR converted to mean (SD) |
| Fassoulaki 2002 | Chronic pain incidence | Not reported (analgesics only) | 0-100mm VAS | Not reported | Not applicable |
| Fassoulaki 2012 | Chronic pain incidence | Not reported (analgesics only) | 0-100mm VAS | Not reported | Not applicable |
| Fawzi 2014 | Neuropathic pain (LANSS ≥ 12) | Not reported | LANSS (used to derive incidence) | Not reported | Not applicable |
| Gaber 2019 | Neuropathic pain (LANSS ≥ 12) | Not reported | LANSS (used to derive incidence) | Respiratory depression | No SAE events |
| Gianesello 2012 | Chronic pain incidence (moderate or extreme pain) | Not reported | Not reported | Respiratory depression | Not applicable |
| Grosen 2014 | Persistent post-thoracotomy pain (any pain) | Not reported | 0-10 NRS (average), 0-10cm VAS, BPI and MPQ (no significant differences in any measure for BPI: p=0.23-0.65 or MPQ: p=0.17-0.54) | Death | Other SAEs reported, most severe included |
| Hah 2018 | Chronic pain incidence | Yes | BPI (not reported) | SAE | Trial terminated early due to futility in primary outcome |
| Homma 2019 | Neuropathic pain (LANSS) | Not reported | 0-10cm VAS and LANSS (used to derive incidence) | Delirium | VAS scores SD estimated from other studies and extracted from graph |
| Joshi 2013 | Not reported | Not reported | 0-10cm VAS (deep breath) | Respiratory depression | 1) No SAE events  2) Pain scores extracted from graph and deep breathing used as no pain at rest |
| Khurana 2014 | Not reported | Not reported | 0-100mm VAS (static) and ODI (significant difference between pregabalin and placebo: p<0.05) | Respiratory depression | 1) Data extracted from graphs  2) SD estimated from other studies  3) No SAE events |
| Kim 2010 | Chronic pain incidence (any pain) | Not reported | Not reported | Not reported | Not applicable |
| Kinney 2012 | Chronic pain incidence | Not reported | Not reported | Respiratory depression | SAE taken from number needing naloxone in PACU |
| Konstantatos 2016 | Pain at incision | Not reported | 0-10cm VAS (rest) and SF-MPQ (no significant difference at 9 months: p=0.27) | Death | Pain scores extracted from graph with median and IQR converted to mean (SD) |
| Koşucu 2014 | Neuropathic pain (LANSS > 12) | Not reported | Not reported | Respiratory depression | SAE not reported |
| Macheridou 2012 (*unpublished*) | Neuropathic pain | Not reported | DN4 (used for incidence of neuropathic pain) | Not reported | Data taken from conference abstract |
| Moore 2011 | Chronic pain incidence (any pain) | Not reported (analgesics only) | 0-10 NRS | Not reported | 1) Median and range (divide by 4) converted to mean (SD)  2) Pain severity from those with pain |
| Myhre 2017 | Chronic pain incidence (wound pain at rest < 24 hours) | Not reported | Not reported | Not reported | Not applicable |
| Nikolajsen 2006 | Phantom pain | Yes (oral morphine mg/day) | 0-10 scale (phantom pain) and MPQ (states no significant difference) | Death | 1) SAE not included as occurred at 3-6 months 2) Data extracted from graphs  3) Median and IQR converted to mean (SD) 4) Data for pain severity taken from 6 months as unclear median for 3 months on graph |
| Pesonen 2011 | Chronic pain incidence (VRS ≥ 1 at rest) | Yes (tramadol use) | 0-4 VRS (used to derive incidence) | Delirium (requiring treatment on ICU) | Not applicable |
| Petersen 2018 | Chronic pain incidence (VAS > 30mm) | Not reported | 0-100mm VAS (rest) | Not reported | 1) Data extracted from graphs  2) Incidence of pain not reported fully but no significant difference: p=0.41  3) Gabapentin groups combined |
| Quail 2017 | Not reported | Not reported | 0-100mm VAS | Not reported | Not applicable |
| Reyad 2019 | Neuropathic pain | Not reported | 0-100mm VAS | Not reported | Not applicable |
| Sadatsune 2016 | Chronic pain incidence (NRS ≥ 4) | Yes (codeine use) | 0-10 NRS (used for incidence) and DN4 (used for incidence of neuropathic pain) | Not reported | Pain severity not reported as continuous data |
| Sen 2009a | Not reported | Not reported | 0-10 NRS | Not reported | Data extracted from graph |
| Shimony 2016 | Not reported | Not reported (analgesics only) | 0-10 NRS | Prolonged ventilation | Not applicable |
| Short 2012 | Pain or abnormal sensations at wound | Not reported (analgesics only) | 0-10 VRS | Not reported | Pain incidence and intensity not reported so not included (*author contacted, no reply)* |
| Sidiropoulou 2016 | Chronic pain incidence (any pain on VRS) | Not reported (analgesics only) | 0-3 VRS and DN4 (for neuropathic pain incidence, lower in pregabalin group: p<0.001) | Not reported | Excluded group that also received wound LA infusion |
| Singla 2015a | Chronic pain incidence (pain at surgical site) | Not reported (1 week only) | NPSI (reported as similar between groups, no p value) | Not reported (adverse events only) | 1) Data used from 6 months as 3 months not fully reported  2) Intention to treat for efficacy used for participant numbers |
| Singla 2015b | Chronic pain incidence (pain at surgical site) | Not reported (1 week only) | Not reported | Not reported (adverse events only) | Only data for one pregabalin group reported |
| Singla 2015c | Chronic pain incidence (pain at surgical site) | Not reported (1 week only) | Not reported | Not reported (adverse events only) | Only data for one pregabalin group reported |
| Ucak 2011 | Chronic pain incidence | Not reported | 0-10 NRS (cough) | Death | 1) No SAE events  2) Pain on cough used as only value that could be extracted from graph |
| Vig 2019 | Chronic pain incidence (any pain) | Not reported | 0-10 NRS (used to derive incidence of severe pain) | Not reported | Not applicable |
| YaDeau 2015 | Neuropathic pain (LANSS ≥ 12) | Opioid use (mg) | 0-10 NRS (flexion) and LANSS (no difference between groups: p=0.94) | Delirium | 1) Data extracted from graphs  2) 95% CI converted to SD using primary outcome number of participants  2) Pregabalin groups combined  3) Unable to use opioid data as unclear what opioid mg represented or incidence of pain as not reported separately for each group (*author contacted, no reply)* |
| Zarei 2016 | Not reported | Not reported | 0-10 NRS (back pain) and ODI (no significant difference between groups: p=0.1) | Not reported | Pregabalin groups combined |
| ***Pregabalin/Gabapentin and Glucocorticoids (GABGLU)*** | | | | | |
| Choi 2013 | Not reported | Not reported | 0-100mm VAS (back pain at rest) | Not reported | Median and IQR converted to mean (SD) |
| Momon 2019 | Chronic pain incidence (back pain) | Yes (regular opioid intake) | 0-10 NRS (only those with pain) and DN4 (for neuropathic pain incidence, no difference: p=0.57) | Respiratory disturbance (unclear if depression) | 1) Unclear how many in each group so % used to calculate N  2) Median and IQR converted to mean (SD) 3) Only those with pain included in pain intensity |
| ***Pregabalin/Gabapentin and Ketamine (GABKET)*** | | | | | |
| Anwar 2019 | Chronic pain incidence (NRS > 3 after coughing) | Not reported (analgesics only) | 0-10 NRS and LANSS (used to derive incidence of neuropathic pain, significantly lower in treatment groups: p=0.003-0.055) | Death, prolonged ventilation, re-intubation and renal failure | Not applicable |
| Çelebi 2013 | Chronic pain incidence (at incision) | Not reported | Not reported | Not reported | Translated from Turkish |
| Martinez 2014 | Not reported | Not reported | Not reported | Not reported | Included as chronic pain data in another review (*author contacted*) |
| Sen 2009b | Chronic pain incidence (not reported) | Not reported | 0-10 VRS | Not reported | 1) Data extracted from graphs  2) SD estimated from other studies as unclear on graph *(author contacted)* |
| ***Pregabalin/Gabapentin and NSAIDs (GABNSA)*** | | | | | |
| Matsutani 2015 | Neuropathic pain (LANSS > 11) but only until 8 weeks | Not reported (analgesics only at one week) | 0-10 NRS | Not reported | 1) Unclear SAEs  2) Data extracted from graphs |
| ***Pregabalin/Gabapentin and Paracetamol (GABPAR)*** | | | | | |
| Pavelescu 2009 (*unpublished*) | Chronic pain incidence | Not reported | Not reported | Not reported | Conference abstract with no data that can be used (*author contacted*) |

**Table 4:** Included outcomes from each study with definitions and details of SAEs. Abbreviations: AKI: acute kidney injury, BPI: brief pain inventory, CI: confidence interval, COMI: core outcomes measurement index, DN4: douleur neuropathique 4, FUPQ: follow-up pain questionnaire, IASP: International Association for the Study of Pain, ICH: International Conference of Harmonization, IQR: inter-quartile range, LANSS: Leeds assessment of neuropathic symptoms and signs, m: modified, MI: myocardial infarction, MPQ: McGill pain questionnaire, NPQ: neuropathic pain questionnaire, NRS: numeric rating scale, NPSI: neuropathic pain symptom inventory, ODI: Oswestry disability index, PACU: post-anaesthesia care unit, QEDN: le questionnaire d’evaluation de la douleur neurologique, SAE: serious adverse event, SD: standard deviation, SF: short form, SF-36: 36-item short form survey, VAS: visual analogue scale, VRS: verbal rating scale, WOMAC: Western Ontario and McMaster universities arthritis index.

# **Risk of bias in included studies**

| **Author and year** | **Randomisation** | **Allocation concealment** | **Blinding (participants and personnel)** | **Blinding (outcomes)** | **Attrition** | **Reporting** | **Other bias** |
| --- | --- | --- | --- | --- | --- | --- | --- |
| ***Alpha-2 agonists (ALP)*** | | | | | | | |
| Alzeftawy 2015 | Low (computer) | Low (third party) | Low (identical placebo) | Low (blinded) | Low (3 participants lost to follow up) | High (chronic pain severity not fully reported) | Low (no other issues) |
| Bielka 2018 | Low (computer) | Low (blinded) | Low (identical placebo) | Low (likely blinded from previous information) | Low (all participants analysed) | Low (chronic pain pre-specified in NCT03211871) | Low (no other issues) |
| Han 2019a | Low (computer) | Unclear (no mention) | Low (identical placebo) | Unclear (unclear if chronic pain blinded) | High (high attrition rate: 14%) | Unclear (no protocol) | Low (no other issues) |
| Jain 2012 | Low (computer) | Unclear (no mention) | Low (identical placebo) | Unclear (no mention) | High (high attrition rate: 20%) | Unclear (no protocol) | Low (no other issues) |
| Mao 2020 | Low (computer) | Low (sealed, opaque envelopes) | Low (identical placebo) | Low (blinded) | Low (low attrition rate: <10%) | Low (chronic pain pre-specified in ChiCTR1800015054) | Low (no other issues) |
| ***Alpha-2 agonists and NSAIDs (ALPNSA)*** | | | | | | | |
| Sessler 2020 | Low (computer) | Low (blinded) | Low (placebo) | Low (blinded) | Low (0.5% attrition) | Low (chronic pain pre-specified as per methods) | Unclear (cannot identify as not reported in subgroups) |
| ***Glucocorticoids (GLU)*** | | | | | | | |
| Bergeron 2009 | Unclear (no mention) | Unclear (no mention) | Low (identical placebo, prepared by research member not involved in data collection) | Low (blinded) | High (19 participants lost to follow up) | Unclear (no protocol) | High (dexamethasone group had lower pre-operative pain scores) |
| Chan 2020 | Low (computer) | Unclear ('sealed envelopes') | Low (identical placebo) | Low (blinded) | Low (one participant lost to follow up) | High (chronic pain not pre-specified in NCT02767882) | Low (no other issues) |
| Jeyamohan 2015 | Unclear (no mention) | Unclear (no mention) | Low (identical placebo) | Low (blinded) | High (high attrition rate at 12 months) | High (chronic pain not pre-specified in NCT01065961) | High (8 participants in placebo group received steroids for airway problems) |
| Nielsen 2015 | Low (computer) | Low (pharmacy and sealed, opaque envelopes) | Low (identical placebo) | Low (likely blinded from previous information) | High (response rate only 73% at 3 months) | High (chronic pain not pre-specified in NCT01953978) | Low (no other issues) |
| Nielsen 2016 | Low (computer) | Low (pharmacy and sealed, opaque envelopes) | Low (identical placebo) | Low (likely blinded from previous information) | Unclear (only 65% in follow up but no difference on multiple imputation) | High (chronic pain not pre-specified in NCT01953978) | Low (no other issues) |
| Turan 2015 | Low (computer) | Low (web and phone service) | Low (identical placebo) | Low (blinded) | Low (low drop out rate and similar results on worst case imputation) | High (chronic pain not pre-specified in NCT00427388) | Low (no other issues) |
| Weis 2006 | Low (computer) | Unclear (no mention) | Low (identical placebo) | Unclear (no mention) | High (high attrition rate of 22%) | Unclear (no protocol) | Low (no other issues) |
| ***Ketamine (KET)*** | | | | | | | |
| Bakr 2014 | Unclear (no mention) | Unclear (no mention) | Low (identical placebo) | Unclear (no mention) | Low (all participants analysed) | High (SAE not reported in results) | Low (no other issues) |
| Bilgen 2012 | Low (computer) | Low (third party) | Low (identical placebo) | Low (blinded) | Low (all participants analysed) | Unclear (no protocol) | Low (no other issues) |
| Borys 2020 | Low (computer) | Low (third party and concealed in sealed envelopes) | High (no placebo used) | Low (likely blinded as acute pain was) | High (high attrition rate at 3 months) | Low (chronic pain pre-specified in ACTRN12616000900415) | High (more females in control group) |
| Brinck 2020 | Low (computer) | Low (sealed, opaque envelopes) | Low (identical placebo) | Low (blinded) | High (high attrition rate at 24 months: 23%) | Unclear (unable to locate protocol ISRCTN44502772) | High (more females in control group) |
| Cameron 2020 | Low (computer) | Low (third party) | Low (identical placebo) | Low (blinded) | Low (low drop out rate) | High (chronic pain not pre-specified in NCT02925858) | Low (no other issues) |
| Chaparro 2010 | Low (block randomisation) | Low (pharmacy) | Low (placebo) | Low (blinded) | High (high attrition rate of around 50%) | Unclear (no protocol) | Low (no other issues) |
| Chumbley 2019 | Low (computer) | Low (pharmacy) | Low (identical placebo) | Low (blinded) | High (high attrition rate at 12 months: 27%) | High (chronic pain after 6 weeks not pre-specified in NCT01296347) | High (more females in ketamine group and small differences in analgesic regimens) |
| Crousier 2008 | Unclear (no mention) | Unclear (no mention) | Low (placebo) | Low (blinded) | High (high attrition in ketamine group: 33%) | Low (chronic pain pre-specified in NCT00129597) | High (older age in ketamine group) |
| Czarnetzki 2020 | Low (computer) | Low (pharmacy) | Low (identical placebo) | Low (blinded) | High (high attrition rate at 12 months in placebo group: 16%) | Low (chronic pain pre-specified in NCT00618423) | Low (small difference in strong opioids pre-operatively, unlikely significance) |
| De Kock 2001 | Low (computer) | Unclear (no mention) | Low (identical placebo) | Low (blinded) | Low (low drop out rate) | Unclear (no protocol) | Low (no other issues) |
| Dualé 2009 | Unclear (no details) | Unclear ('sealed envelope') | Low (identical placebo) | Low (blinded) | High (high attrition rate at 4 months: 20%) | Low (chronic pain pre-specified in NCT00313378) | High (more females in placebo group) |
| Dullenkopf 2009 | Low (computer) | Unclear (no details) | Low (identical placebo) | Low (blinded) | High (high attrition rate at 3 months: 27%) | Unclear (no protocol) | High (more females in ketamine group) |
| Han 2019b (*unpublished*) | Low (computer) | Low ('sequentially numbered opaque envelopes') | Low (identical placebo) | Low (blinded) | High (high attrition rate at 3 months: 14%) | Low (chronic pain pre-specified in NCT03676114) | Low (no other issues) |
| Hayes 2004 | Low (random number generator) | Unclear (no mention) | Low (identical placebo) | Low (blinded) | High (high attrition rate at 6 months: 29%) | Unclear (no protocol) | High (more females in ketamine group) |
| Hu 2014 | Unclear (no mention or explanation for different N in each group) | Unclear (no mention) | Low (identical placebo) | Unclear (no mention) | Low (low drop out rate) | Unclear (no protocol) | Low (no other issues) |
| Joseph 2012 | Low (computer) | Unclear (no mention) | Low (identical placebo) | Unclear (no mention) | High (high attrition rate of 38%) | Low (chronic pain pre-specified in NCT00726258) | High (duration of surgery longer in ketamine group) |
| Kang 2020 | Low (computer) | Low (pharmacy) | Low (identical placebo) | Low (blinded) | Low (low drop out rate) | Low (chronic pain pre-specified in KCT0000962) | Low (no other issues) |
| Katz 2004 | Low (computer) | Low (pharmacy and sealed opaque envelopes) | Low (identical placebo) | Low (blinded) | High (high attrition rate at 6 months) | Unclear (no protocol) | Low (no other issues) |
| Lee 2018 | Low (computer) | Low (third party and opaque envelopes) | Low (identical placebo) | Low (blinded for chronic pain assessment) | High (high attrition rate at 3 months: 23%) | Low (chronic pain pre-specified in NCT01997801) | Low (no other issues) |
| Lou 2017 | Unclear (no mention or explanation for different N in each group) | Unclear (no mention or explanation for different N in each group) | Low (identical placebo) | Unclear (no mention) | Low (low drop out rate: 5%) | Unclear (no protocol) | Unclear (not enough information) |
| Malek 2006 | Unclear (no mention or explanation for different N in each group) | Unclear (no mention or explanation for different N in each group) | Low (identical placebo) | Unclear (no mention) | High (high attrition rate: 14%) | Unclear (no protocol) | Low (no other issues) |
| Mendola 2012 | Low (computer) | Unclear (no mention) | Low (identical placebo) | Low (blinded) | High (high attrition rate at 3 months) | Unclear (no protocol) | Low (no other issues) |
| Nielsen 2017 | Low (computer) | Low (pharmacy and sealed, opaque envelopes) | Low (identical placebo) | Low (blinded) | High (high attrition rate of 37% at 6 months) | Low (chronic pain pre-specified in NCT02085577) | Low (no other issues) |
| Nielsen 2019 | Low (computer) | Low (pharmacy and sealed, opaque envelopes) | Low (identical placebo) | Low (blinded) | High (high attrition rate at 12 months, response rate 67%) | Low (chronic pain pre-specified in NCT02085577) | Low (no other issues) |
| Perrin 2009 | Unclear (no details) | Low (offsite) | Low (identical placebo) | Unclear (no mention) | High (terminated early and high attrition) | Unclear (no protocol) | Low (high pain excluded but only one participant) |
| Pehlivan 2019 | Unclear ('sealed envelope method') | Unclear ('sealed envelope method') | Low (identical placebo) | Unclear (no mention) | Low (all participants analysed) | Unclear (no protocol) | High (more females in placebo group) |
| Peyton 2017 | Unclear (sealed envelopes) | Unclear (sealed envelopes) | Low (identical placebo) | Low (blinded) | Low (intention to treat on all participants) | Unclear (unable to locate protocol ANZCTRN12614000247673) | High (more females in placebo group and variable analgesia regimens) |
| Remerand 2009 | Low (computer) | Low (third party) | Low (identical placebo) | Low (blinded) | Low (low drop out rate) | Unclear (no protocol) | Low (no other issues) |
| Spreng 2010 | Low (permuted block randomisation) | Low (pharmacy and sealed envelopes) | Low (identical placebo) | Low (blinded) | Low (low drop out rate) | High (chronic pain not pre-specified in NCT00354029) | Low (no other issues) |
| Suppa 2012 | Low (computer) | Unclear (no mention) | Low (identical placebo) | Unclear (no mention) | High (high attrition rate at 36 months: >50%) | High (did not report 6 month data) | Low (no other issues) |
| Suzuki 2006 | Low (computer) | Unclear (no details) | Low (identical placebo) | Low (blinded) | Low (low drop out rate) | Unclear (no protocol) | Low (no other issues) |
| Sveticic 2008 | Low (drawing lots) | Unclear (no details) | Low (identical placebo) | Low (blinded) | High (most participants did not return chronic pain questionnaires) | Unclear (no protocol) | Low (no other issues, although different anaesthetics, these were equal between groups) |
| Tena 2014 | Low (computer) | Unclear (no details) | Low (identical placebo) | Low (blinded) | High (high attrition although unclear which groups) | Low (chronic pain pre-specified in NCT01243801) | Low (no other issues) |
| ***Ketamine and Glucocorticoids (KETGLU)*** | | | | | | | |
| Galinski 2007 | Unclear ('sealed envelope') | Unclear ('sealed envelope') | Low (double-dummy placebo) | Unclear (no mention) | High (high attrition rate: 17%) | High (SAE not reported) | Low (no other issues) |
| Shanthanna 2020 | Low (computer) | Low (website randomisation) | Low (placebo) | Low (blinded) | Low (intention to treat on all participants) | Low (chronic pain pre-specified in NCT02950233) | Low (no other issues) |
| Pavelescu 2020 (*unpublished*) | Unclear (no mention) | Unclear (no mention) | Unclear (no mention) | Unclear (no mention) | Unclear (no mention) | Unclear (no mention) | Unclear (no mention) |
| ***Ketamine and Nefopam (KETNEF)*** | | | | | | | |
| Aveline 2014 | Low (computer) | Low (sealed, opaque envelopes) | Low (placebo) | Low (blinded) | Low (response rate: 92%) | Unclear (no protocol, although month 6 incidence not reported as per month 12, this seems to be deliberate) | Low (no other issues) |
| ***Lidocaine (LID)*** | | | | | | | |
| Beaussier 2018 | Low (block randomisation) | Low (performed centrally) | Low (placebo for both IV infusion and wound infusion) | Low (likely blinded from previous information) | High (high attrition rate despite stating intention to treat: 27%) | Low (chronic pain pre-specified in NCT01077752) | High (more females in lidocaine group) |
| Choi 2017 | Low (website randomisation) | Low (website randomisation) | Low (placebo) | Low (blinded) | Low (low drop out rate) | Low (chronic pain stated with likely error in time-point in NCT01907997) | Low (no other issues) |
| Dai 2020 | Low (random number table) | Unclear (no details) | Low (placebo) | Low (blinded) | Low (low drop out rate) | High (SAE not reported) | Low (no other issues) |
| Galos 2020 | Low (computer) | Unclear (no mention) | High (identical placebo but clinicians not blinded intra-operatively) | Low (blinded) | High (high attrition rate: 20%) | High (chronic pain by MPQ not stated in NCT02839668) | Low (no other issues) |
| Ghimire 2020 | Low (computer) | Low (sealed, opaque envelopes) | Low (placebo) | Low (blinded) | Low (low drop out rate and intention to treat) | Low (chronic pain pre-specified in NCT02601651) | Low (no other issues) |
| Grigoras 2012 | Low (computer) | Low (opaque envelopes and opened by third party) | Low (placebo) | Low (blinded) | Low (all participants analysed) | Unclear (no protocol) | High (some participants underwent further surgery, more in control group) |
| Ibrahim 2018 | Unclear ('closed envelopes') | Unclear ('closed envelopes') | Low (placebo) | Unclear (no mention) | Low (low drop out rate) | Low (chronic pain pre-specified in NCT03030560) | Low (no other issues) |
| Kendall 2018 | Low (using R) | Low (sealed, opaque envelopes) | Low (identical placebo) | Low (blinded) | High (high attrition rate: 19% at 6 months) | Low (chronic pain pre-specified in NCT01619852) | Low (no other issues) |
| Martin 2008 | Unclear ('randomization list') | Unclear ('sealed envelopes') | Low (placebo) | Low (blinded) | Low (low drop out rate) | Unclear (no protocol) | High (two excluded from lidocaine group for extreme pain) |
| Terkawi 2015 | Low (website randomisation) | Low (website and sealed, opaque envelopes) | Low (placebo) | Low (blinded) | High (high attrition rate: 24%) | Low (chronic pain pre-specified in NCT01204242) | High (analgesia not standardised and more radical mastectomies in lidocaine group) |
| Toner 2021 | Low (computer) | Low (pharmacy had only access and sealed, opaque envelopes) | Low (placebo) | Low (likely blinded from previous information) | Low (low drop out rate) | Low (chronic pain pre-specified in ACTRN12618000463279) | Low (no other issues) |
| Vasilev 2019 | Unclear (no details) | Unclear (no details) | Low (placebo) | Unclear (no mention) | Low (low drop out rate at 3 months, 12 month data could not be included) | High (did not fully report 12 month data) | Low (no other issues) |
| ***Lidocaine and Alpha-2 agonists (LIDALP)*** | | | | | | | |
| Rekatsina 2019 (*unpublished*) | Unclear (no mention) | Unclear (no mention) | Low (identical placebo) | Unclear (no mention) | High (high attrition rate: 19%) | High (chronic pain not pre-specified in NCT03363425) | Unclear (no mention) |
| ***Lidocaine and Pregabalin/Gabapentin (LIDGAB)*** | | | | | | | |
| Khan 2019 | Low (computer) | Low (centralised system) | Low (identical placebo) | Low (blinded) | Low (all participants analysed) | High (time-point changed to 3 months from NCT02240199) | High (not presented in subgroups for single interventions and some differences in analgesia given) |
| ***Lidocaine and Ketamine (LIDKET)*** | | | | | | | |
| Dhir 2020 | Low (website randomisation) | Low (website randomisation and opaque envelopes with third party) | Low (placebo) | Low (blinded) | Low (low drop out rate: <10%) | Unclear (retrospective registration) | High (less females in ketamine group) |
| Jendoubi 2017 | Unclear ('sealed envelopes') | Unclear ('sealed envelopes') | Low (placebo) | Low (blinded) | Low (low drop out rate) | Low (chronic pain pre-specified in NCT02653651) | Low (no other issues) |
| ***Lidocaine and Magnesium (LIDMAG)*** | | | | | | | |
| Kim 2017 | Low (website randomisation) | Low (website randomisation and sealed envelopes) | Low (placebo) | Low (blinded) | Low (low drop out rate) | High (chronic pain not pre-specified in NCT02185859) | High (differences in extent of surgery between groups) |
| ***Nefopam (NEF)*** | | | | | | | |
| Kim 2018 | Low (computer) | Unclear (performed by anaesthesiologist, unclear role) | Low (identical placebo) | Low (blinded) | Low (all participants analysed) | Low (chronic pain pre-specified in KCT0001950) | Low (no other issues) |
| Na 2016 | Low (computer) | Unclear (performed by anaesthesiologist, unclear role) | Low (identical placebo) | Unclear (no mention) | High (high attrition rate: 12%) | Low (chronic pain pre-specified in NCT02561468) | Low (no other issues) |
| ***NSAIDs/COX-2 inhibitors (NSA)*** | | | | | | | |
| Comez 2015 | Unclear (no details) | Unclear (no mention) | High (no intravenous placebo used although states double-blind) | Unclear (no mention) | Low (all participants analysed) | Unclear (no protocol) | High (difference in sex and surgical time between groups) |
| Haddad 2019 | Low (random number table) | Low (third party) | Low (saline placebo) | Low (likely blinded from previous information) | High (high attrition rate as only 79% analysed) | Unclear (no protocol) | High (difference in sex between groups) |
| Fransen 2006 | Low (computer) | Low (telephone service and states blinded) | Low (identical placebo) | Low (blinded) | Low (low attrition rate and intention to treat) | Low (chronic pain pre-specified in NCT00145730) | Low (no other issues) |
| Ge 2021 (*unpublished*) | Low (computer) | Unclear (no details) | Low (identical placebo) | Low (blinded) | Low (low attrition rate and intention to treat) | Unclear (chronic pain pre-specified in ChiCTR-2000035198 but retrospectively registered) | Low (higher depression in parecoxib group but not clinically significant) |
| Lakdja 1997 | Unclear (no details) | Unclear (no details) | Low (placebo) | Unclear (no details) | Low (low attrition rate) | Unclear (no protocol) | Low (no other issues) |
| Ling 2016 | Unclear (no details) | Unclear (no details) | Low (placebo) | Unclear (no mention) | High (high attrition rate: 88% analysed at one year follow up) | Unclear (no protocol) | High (less females in placebo group) |
| Sun 2013 | Low (computer) | High (anaesthetist performed randomisation and anaesthesia) | Unclear (unclear if intralipid placebo appeared different) | Low (blinded) | Low (all participants analysed) | High (did not report analgesic use) | Low (no other issues) |
| van Helmond 2016 | Low (computer) | Low (pharmacy) | Low (blinded packages and placebo) | Low (likely blinded from previous information) | High (high attrition rate as only 68% analysed) | High (did not report pain incidence fully for each group) | Unclear (grant from Pfizer but not involved) |
| ***NSAIDs/COX-2 inhibitors and Glucocorticoids (NSAGLU)*** | | | | | | | |
| Romundstad 2006 | Low (block randomisation) | Low (third party) | Low (identical placebo) | Low (blinded) | High (high attrition rate at 12 months: 20%) | Unclear (no protocol) | Low (no other issues) |
| ***Paracetamol (PAR)*** | | | | | | | |
| Koyuncu 2018 | Low (website randomisation) | Low (website randomisation) | Low (placebo) | Low (blinded) | Low (all participants analysed despite 4 exclusions) | Low (chronic pain pre-specified in NCT02086747. Although SAE not reported, it was as a surrogate of respiratory rate) | Low (no other issues) |
| Turan 2017 | Low (computer) | Low (website randomisation close to anaesthetic induction) | Low (placebo) | Low (blinded) | High (high attrition rate: 30%) | High (chronic pain not pre-specified in NCT01822821) | Low (no other issues) |
| ***Paracetamol and Nefopam (PARNEF)*** | | | | | | | |
| Lee 2017 (*unpublished*) | Unclear (no mention) | Unclear (no mention) | Unclear (no mention) | Unclear (no mention) | Unclear (no mention) | High (unpublished, chronic pain not fully reported in abstract) | Unclear (no mention) |
| ***Pregabalin/Gabapentin (GAB)*** | | | | | | | |
| Acin 2009 | Unclear (no mention) | Unclear (no mention) | High (no placebo mentioned) | Unclear (no mention) | Low (all participants analysed) | Unclear (no protocol) | Low (no other issues) |
| Ahiskalioglu 2016 | Low (computer) | Unclear (no mention) | Low (identical placebo) | Unclear (no mention) | Unclear (no mention) | High (ACTRN1265000435583 but cannot locate protocol, chronic pain not reported fully) | Low (no other issues) |
| Amr 2010 | Unclear ('sealed envelope') | Low ('blindly randomized') | Low (identical placebo) | Unclear (no mention) | Low (all participants analysed) | High (chronic pain score not reported at rest) | Low (no other issues) |
| Bouzia 2017 | Low (computer) | Unclear (no mention) | Low (placebo) | Low (blinded) | Low (low drop out rate: <10%) | High (chronic pain pre-specified in NCT01701921  but incidence not reported in results, as specified in methods) | Low (no other issues) |
| Buvanendran 2010 | Low (computer) | Low (pharmacy) | Low (identical placebo) | Low (blinded) | Low (intention to treat analysis with low attrition) | Unclear (no protocol) | Unclear (received grant from Pfizer but methods state no involvement) |
| Brogly 2008 | Low (randomisation table) | Unclear (no mention) | Low (placebo) | Unclear (no mention) | Low (low drop out rate) | Unclear (no protocol) | Low (no other issues) |
| Brulotte 2015 | Low (computer) | Low (pharmacy) | Low (identical placebo) | Low (likely blinded from previous information) | High (high attrition rate of 13%) | Low (chronic pain pre-specified in NCT00967135) | High (more females in pregabalin group) |
| Burke 2010 | Unclear (no details) | Low (sealed, opaque envelopes) | Low (placebo) | Unclear (no mention) | Low (low drop out rate) | Unclear (no protocol) | High (received grant from Pfizer and more females in placebo group) |
| Clarke 2009 | Low (computer) | Low (pharmacy and investigators had no access) | Low (identical placebo) | Low (likely blinded from previous information) | High (high attrition rate at 6 months: 35%) | Unclear (no protocol) | Low (no other issues) |
| Clarke 2014 | Low (computer) | Low (pharmacy and investigators had no access) | Low (identical placebo) | Low (blinded) | High (high attrition rate if calculated from those consented: 27%) | Unclear (no protocol) | High (more females in gabapentin group) |
| Clarke 2015 | Low (computer) | Low (pharmacy and investigators had no access) | Low (identical placebo) | Low (blinded) | High (high attrition rate at 3 months: 29%) | Unclear (no protocol) | Low (no other issues) |
| Fassoulaki 2002 | Unclear (no details) | Unclear (sealed envelopes but description unclear to judge) | Low (identical placebo) | Low (blinded) | Low (low drop out rate) | Unclear (no protocol) | Low (no other issues) |
| Fassoulaki 2012 | Low (computer) | Unclear (sealed envelopes) | Low (identical placebo) | Unclear (no mention) | High (high attrition rate at 3 months: 22%) | Unclear (no protocol) | Low (no other issues) |
| Fawzi 2014 | Low (computer) | Unclear (opaque envelope) | High (tablets have different letters on, Lyrica vs Ferrex 50) | Low (blinded) | Low (all participants analysed) | Unclear (no protocol) | Low (no other issues) |
| Gaber 2019 | Unclear (no mention) | Unclear (no mention) | Unclear (multi-vitamin, unclear what appearance) | Unclear (no mention) | Low (all participants analysed) | Unclear (no protocol) | High (more pneumo-nectomies in placebo group) |
| Gianesello 2012 | Low (computer) | Unclear (no mention) | Low (identical placebo) | Low (blinded) | Low (all participants analysed) | Unclear (no protocol) | High (more females in pregabalin group) |
| Grosen 2014 | Low (computer) | Low (pharmacy and sealed, opaque envelopes) | Low (identical placebo, prepared by nurse not involved in study) | Low (likely blinded from previous information) | High (high attrition rate at 3 months: 64% response rate) | High (chronic pain pre-specified at 3 months although protocol states up to 12 months in NCT01116583) | High (more females in gabapentin group) |
| Hah 2018 | Low (computer) | Low (pharmacy and states blinded allocation) | Low (identical, active placebo where participants couldn't tell they were receiving gabapentin: p=0.3) | Low (likely blinded from previous information) | Low (low drop out rate with modified intention to treat) | Low (chronic pain and opioid use pre-specified in NCT01067144) | Low (similar groups including analgesia) |
| Homma 2019 | Low (computer) | Unclear (no mention) | High (no placebo mentioned) | Low (blinded) | Low (low drop out rate) | High (chronic neuropathic pain incidence not pre-specified in UMIN 000007757) | High (more males in pregabalin group and difference in procedures) |
| Joshi 2013 | Unclear ('closed envelope method') | Unclear ('closed envelope method') | Low (similar placebo) | Low (not part of postoperative management) | Low (all participants analysed) | Unclear (no protocol) | Low (no other issues) |
| Khurana 2014 | Low (computer) | Low (sealed, opaque envelopes) | Low (identical placebo) | Low (blinded) | Low (all participants analysed) | Unclear (no protocol) | Low (no other issues) |
| Kim 2010 | Unclear (no details) | Unclear (no details) | Low (identical placebo) | Low (blinded) | Low (low drop out rate: 95% response rate) | Low (hypoesthesia pre-specified in NCT00905580) | Low (no other issues) |
| Kinney 2012 | Low (block randomisation) | Unclear (no details) | Low (identical active placebo) | Low (blinded) | High (high attrition rate: 18%) | Low (chronic pain pre-specified in NCT00588159) | Low (no other issues) |
| Konstantatos 2016 | Low (computer) | Low (pharmacy and opaque envelopes) | Low (identical placebo) | Low (likely blinded from previous information) | Low (intention to treat analysis) | Unclear (no protocol) | Low (no other issues) |
| Koşucu 2014 | Unclear ('sealed envelope') | Unclear ('sealed envelope') | Low (identical placebo) | Unclear (unclear for chronic pain) | Low (low drop out rate) | High (no protocol but SAE not reported) | Low (no other issues) |
| Macheridou 2012 (*unpublished*) | Unclear (no details) | Unclear (no mention) | Low (placebo) | Unclear (no mention) | Low (low drop out rate at 3 months) | High (unpublished abstract) | Low (quoted as similar groups) |
| Moore 2011 | Low (computer) | Low (pharmacy) | Low (identical placebo) | Low (likely blinded from previous information) | High (high attrition rate at 3 months: 78% response rate) | Low (chronic pain pre-specified in NCT00573664) | Low (no other issues) |
| Myhre 2017 | Low (computer) | Low (third party and opaque, sealed envelopes) | Low (identical placebo) | Low (likely blinded from previous information) | Low (low drop out rate, even at 12 months) | High (chronic pain not pre-specified in NCT01059331) | Low (no other issues) |
| Nikolajsen 2006 | Low (computer) | Low (pharmacy) | Low (identical placebo) | Low (likely blinded from previous information) | High (high attrition rate: 26%) | Unclear (no protocol) | Unclear (Pfizer supplied drugs but unclear role) |
| Pesonen 2011 | Low (computer) | Low (pharmacy) | Low (identical placebo) | Low (blinded) | High (high attrition rate: 19%) | Unclear (no protocol) | High (more females in placebo group) |
| Petersen 2018 | Low (computer) | Low (pharmacy) | Low (identical placebo) | Low (likely blinded from previous information) | High (high attrition rate at 3-4 years: 28%) | High (chronic pain not specified in NCT01507363) | Low (no other issues) |
| Quail 2017 | Low (random number table) | Low (pharmacy) | Low (identical placebo) | Low (likely blinded from previous information) | High (high attrition rate at 3 and 12 months) | Low (chronic pain pre-specified in NCT02419443) | Low (no other issues) |
| Reyad 2019 | Low (computer) | Low (states allocation concealed) | Low (placebo) | Low (blinded) | Low (19 lost to follow up but intention to treat analysis) | Unclear (unable to locate protocol: PACTR201705002266194) | Low (no other issues) |
| Sadatsune 2016 | Low (computer) | Low (sealed, opaque envelopes) | Low (identical placebo) | Low (blinded) | Low (low drop out rate) | Low (chronic pain pre-specified in NCT01632215) | Low (no other issues) |
| Sen 2009a | Low (computer) | Unclear (no details) | Low (identical placebo) | Low (blinded) | Low (low drop out rate of one patient) | Unclear (no protocol) | Low (no other issues) |
| Shimony 2016 | Low (computer) | Low (pharmacy) | Low (identical placebo) | Low (likely blinded from previous information) | High (high attrition rate of around 50%) | High (chronic pain not pre-specified in NCT01612832) | Unclear (high attrition so difficult to assess baseline characteristics) |
| Short 2012 | Low (computer) | Unclear (no details on envelope safeguards) | Low (identical placebo) | Low (blinded for included outcomes) | High (high attrition rate of around 15%) | High (chronic pain pre-specified in NCT01094925 but not fully reported) | Low (no other issues) |
| Sidiropoulou 2016 | Low (computer) | Low (third party and sealed envelopes) | Low (identical placebo) | Low (likely blinded from previous information) | Low (all participants analysed with intention to treat analysis) | Low (chronic pain pre-specified in NCT01726205) | High (more females in placebo group and pneumo-nectomies in pregabalin group) |
| Singla 2015a | Low (computer) | Low (phone and internet system) | Low (identical placebo) | Low (likely blinded from previous information) | Low (low drop out rate in modified intention to treat) | High (chronic pain pre-specified in NCT00551135 but not fully reported) | High (some authors are Pfizer employees) |
| Singla 2015b | Low (computer) | Low (phone and internet system) | Low (identical placebo) | Low (likely blinded from previous information) | High (trial terminated early) | High (chronic pain pre-specified in NCT00442546 but not fully reported) | High (some authors are Pfizer employees) |
| Singla 2015c | Low (computer) | Low (phone and internet system) | Low (identical placebo) | Low (likely blinded from previous information) | Low (low drop out rate in modified intention to treat) | High (chronic pain pre-specified in NCT00468845 but not fully reported) | High (some authors are Pfizer employees) |
| Ucak 2011 | Low (computer) | Unclear (no details) | Low (placebo, prepared by nurse not involved in study) | Low (blinded) | Low (all participants analysed) | Unclear (no protocol) | Low (no other issues) |
| Vig 2019 | Low (computer) | Unclear (opaque envelope) | Low (placebo) | Unclear (unclear for chronic pain) | High (high attrition rate: 11%) | Low (chronic pain pre-specified in CTRI/2017/11/010390) | Low (no other issues) |
| YaDeau 2015 | Low (computer) | Low (third party) | Low (identical placebo) | Low (likely blinded from previous information) | High (high attrition rate at 3 months of 22% when looking at neuropathic pain incidence total) | High (chronic pain pre-specified in NCT01333956 but incidence not fully reported) | High (more females in pregabalin groups) |
| Zarei 2016 | Low (computer) | Unclear (no details) | Low (placebo) | Low (blinded) | Low (all participants analysed) | Unclear (no protocol) | Low (no other issues) |
| ***Pregabalin/Gabapentin and Glucocorticoids (GABGLU)*** | | | | | | | |
| Choi 2013 | Low (computer) | Unclear (no details) | Low (identical placebo) | Low (blinded) | Low (low drop out rate: 90% response rate) | High (chronic pain not pre-specified in NCT01168531) | High (more females in placebo group and higher pain in combined group) |
| Momon 2019 | Unclear ('sealed envelope') | High (seen by anaesthetic team) | Low (double-dummy placebo, similar in appearance) | Low (blinded) | High (high attrition rate when using % to calculate N for pain incidence: 21%) | Low (chronic pain pre-specified in NCT01811251) | High (more females and less opioid patients in placebo group) |
| ***Pregabalin/Gabapentin and Ketamine (GABKET)*** | | | | | | | |
| Anwar 2019 | Low (computer) | Low (pharmacy) | Low (identical placebo) | Low (blinded) | Low (low drop out rate, two missing with imputed values of no pain. Worst case scenario analysis produced similar results) | Low (chronic pain pre-specified in NCT01480765) | Low (no other issues) |
| Çelebi 2013 | Unclear ('closed envelope method') | Unclear ('closed envelope method') | High (no placebo tablets described) | Low (blinded) | Low (all participants analysed) | Unclear (no protocol) | High (gabapentin group had higher number of personal history of chronic pain) |
| Martinez 2014 | Low (computer) | Low (sealed, opaque envelopes) | Low (identical placebo) | Low (blinded) | Low (intention to treat) | Unclear (no protocol) | Low (no other issues) |
| Sen 2009b | Low (computer) | Unclear (no details) | Low (double-dummy placebo, similar appearance) | Low (probably blinded) | Low (all participants analysed) | High (chronic pain incidence not fully reported) | Unclear (limited data) |
| ***Pregabalin/Gabapentin and NSAIDs (GABNSA)*** | | | | | | | |
| Matsutani 2015 | Low (computer) | Unclear (no mention) | High (no placebo tablets mentioned and different dosing schedules) | Unclear (no mention) | Low (all participants analysed) | Unclear (no protocol) | Low (no other issues and no role from funders) |
| ***Pregabalin/Gabapentin and Paracetamol (GABPAR)*** | | | | | | | |
| Pavelescu 2009 (*unpublished*) | Unclear (no mention) | Unclear (no mention) | Unclear (no mention) | Unclear (no mention) | Unclear (no mention) | Unclear (no mention) | Unclear (no mention) |

**Table 5:** Risk of bias for included studies separated by intervention. Green indicates low risk of bias, orange unclear risk of bias and red high risk of bias. Reasons for each judgement are presented in parentheses.

# **Incidence of CPSP ≤ 6 months (single agents)**

**Network meta-regression results**

**Figure 2:** Network plot for the incidence of CPSP ≤ 6 months (single agents). Node size (black) is proportional to the number of studies evaluating that intervention and the grey lines the number of comparisons between each treatment.

| **Number of Interventions** | 10 |
| --- | --- |
| **Number of Studies** | 88 |
| **Total Number of Patients in Network** | 8673 |
| **Total Possible Pairwise Comparisons** | 45 |
| **Total Number of Pairwise Comparisons With Direct Data** | 15 |
| **Is the network connected?** | Yes |
| **Number of Two-arm Studies** | 82 |
| **Number of Multi-Arms Studies** | 6 |
| **Total Number of Events in Network** | 2323 |
| **Number of Studies With No Zero Events** | 83 |
| **Number of Studies With At Least One Zero Event** | 5 |
| **Number of Studies with All Zero Events** | 0 |

| **Treatment** | **Studies** | **Events** | **Participants** | **Average proportion with CPSP** |
| --- | --- | --- | --- | --- |
| **ALP** | 5 | 22 | 164 | 0.13 |
| **GAB** | 33 | 403 | 1806 | 0.22 |
| **GLU** | 4 | 142 | 632 | 0.22 |
| **KET** | 31 | 324 | 1148 | 0.28 |
| **LID** | 12 | 61 | 465 | 0.13 |
| **MAG** | 1 | 8 | 38 | 0.21 |
| **NEF** | 2 | 17 | 63 | 0.27 |
| **NSA** | 5 | 43 | 155 | 0.27 |
| **PAR** | 2 | 28 | 120 | 0.23 |
| **PLA** | 87 | 1275 | 4082 | 0.31 |

**Table 6:** Descriptive statistics of network and treatment characteristics for the incidence of CPSP ≤ 6 months (single agents).

**Figure 3:** Regression plot for the incidence of CPSP ≤ 6 months (single agents) demonstrating the effect of baseline risk (X axis, proportion of participants in the placebo group with pain) compared with the effect estimate (Y axis, log odds). Each colour represents a different intervention. Demonstrates that as baseline risk increases, the reduction in the incidence of CPSP with the intervention increases (more effective).

**Figure 4:** Covariate plot for the incidence of CPSP ≤ 6 months (single agents) showing the distribution of baseline risk (Y axis) in each study (X-axis) grouped by treatment (abbreviation above plot). Red dashed line is the mean. Demonstrates that for some interventions (LID and ALP), baseline risk is lower compared to other interventions (KET and GAB). Such imbalance may violate transitivity assumptions if a standard network meta-analysis was performed.

**Figure 5:** SUCRA plot for ranking of treatments at a covariate value of 0.35 (baseline risk) for the incidence of CPSP ≤ 6 months (single agents). Treatments with lines towards the top left have a higher probability of ranking higher (LID) compared to those at the bottom right (PLA).

| **Intervention** | **SUCRA** |
| --- | --- |
| **Lidocaine (LID)** | 82.7% |
| Alpha-2 agonists (ALP) | 72.6% |
| Magnesium (MAG) | 61% |
| Nefopam (NEF) | 60.4% |
| Paracetamol (PAR) | 55.5% |
| NSAIDs (NSA) | 41.8% |
| **Ketamine (KET)** | 41.6% |
| **Gabapentinoids (GAB)** | 38.6% |
| Glucocorticoids (GLU) | 37.2% |
| Placebo (PLA) | 8.6% |

**Table 7:** SUCRA scores for each intervention studied for the incidence of CPSP ≤ 6 months (single agents). The higher the SUCRA %, the higher the probability that the intervention is top ranked. Note that SUCRA does not take account of risk of bias in included studies or the uncertainty of estimates. Only lidocaine, ketamine and gabapentinoids (highlighted in bold green) demonstrated estimates consistent with a reduction in CPSP despite lower rankings.

**Figure 6:** League heat plot demonstrating relative effects of treatment (X-axis) compared with comparator (Y axis) at a fixed covariate value of 0.35 for the incidence of CPSP ≤ 6 months (single agents). To identify a comparison, the intervention on the X axis is read first then the comparator (Y axis). Green cells are those consistent with a reduction in pain with the intervention (darker colour means stronger effect) whilst red interventions are consistent with an increase in pain (darker colour means stronger effect). Those highlighted with a ****** have credible intervals consistent with an effect (GAB, KET and LID versus PLA for example).

**Figure 7:** Forest plot showing the posterior median odds ratio with 95% CrIs at a fixed covariate value of 0.35 for the incidence of CPSP ≤ 6 months (single agents). Demonstrates possible reductions with ketamine, gabapentinoids and lidocaine. Effects are relative to placebo.

**Diagnostics**

Residual deviance of 193.8 compared with 182 data points.

- The above suggests some problems with model fit
- All PSRF values **<1.0001** (trace and density plots below)


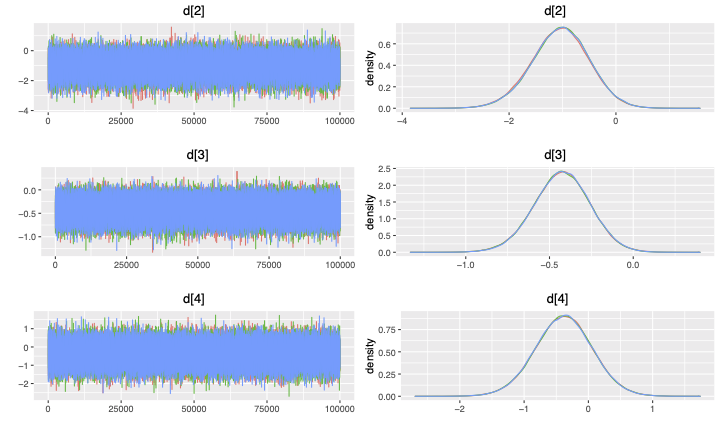


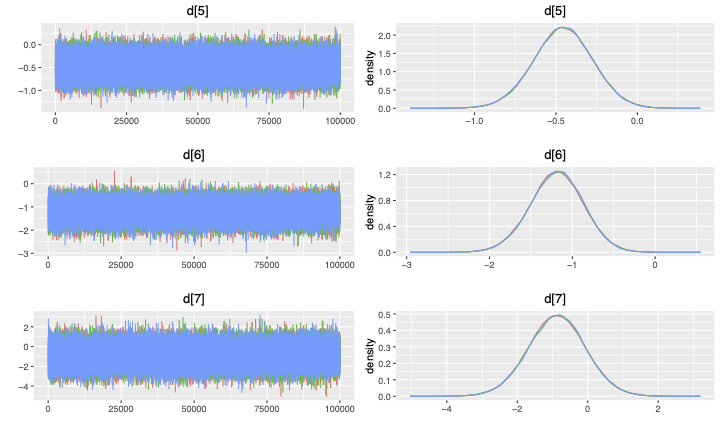


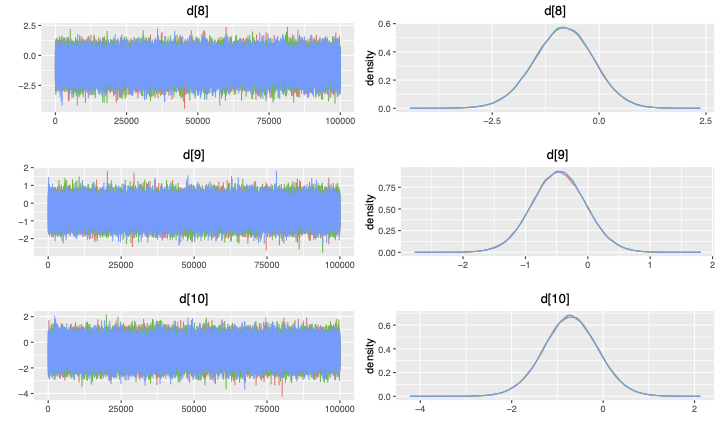


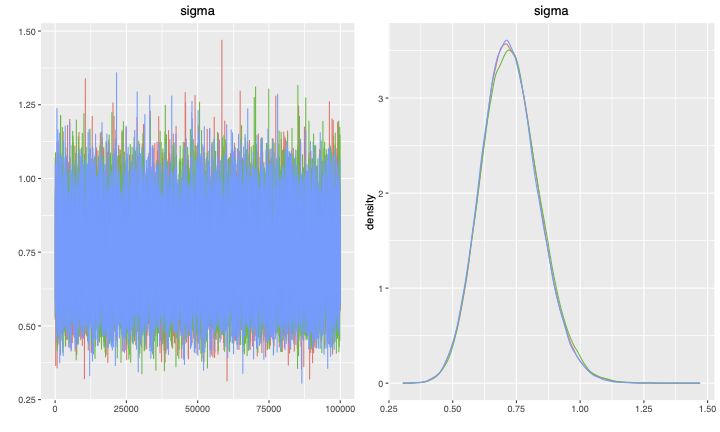


**Inconsistency assessment (no baseline risk adjustment)**

| **Comparison** | **P value** |
| --- | --- |
| GAB-GLU | 0.28 |
| GAB-KET | 0.64 |
| GLU-KET | 0.55 |
| GLU-PLA | 0.48 |
| KET-LID | 0.94 |
| KET-NEF | 0.72 |
| KET-PLA | 0.55 |

**Table 8:** Node-splitting method to assess inconsistency. No evidence of inconsistency although direct estimates were imprecise.

# **Incidence of CPSP ≤ 6 months (combination agents)**

**Network meta-regression results**

**Figure 8:** Network plot for the incidence of CPSP ≤ 6 months (combination agents). Node size (black) is proportional to the number of studies evaluating that intervention and the grey lines the number of comparisons between each treatment.

| **Number of Interventions** | 14 |
| --- | --- |
| **Number of Studies** | 88 |
| **Total Number of Patients in Network** | 8801 |
| **Total Possible Pairwise Comparisons** | 91 |
| **Total Number of Pairwise Comparisons With Direct Data** | 25 |
| **Is the network connected?** | Yes |
| **Number of Two-arm Studies** | 80 |
| **Number of Multi-Arms Studies** | 8 |
| **Total Number of Events in Network** | 2338 |
| **Number of Studies With No Zero Events** | 83 |
| **Number of Studies With At Least One Zero Event** | 5 |
| **Number of Studies with All Zero Events** | 0 |

| **Treatment** | **Studies** | **Events** | **Participants** | **Average proportion with CPSP** |
| --- | --- | --- | --- | --- |
| **ALP** | 5 | 22 | 164 | 0.13 |
| **GAB** | 33 | 403 | 1806 | 0.22 |
| **GABGLU** | 1 | 11 | 33 | 0.33 |
| **GABKET** | 1 | 1 | 50 | 0.02 |
| **GLU** | 4 | 142 | 632 | 0.22 |
| **KET** | 31 | 324 | 1148 | 0.28 |
| **KETGLU** | 1 | 2 | 16 | 0.13 |
| **LID** | 12 | 61 | 465 | 0.13 |
| **LIDKET** | 1 | 1 | 29 | 0.03 |
| **MAG** | 1 | 8 | 38 | 0.21 |
| **NEF** | 2 | 17 | 63 | 0.27 |
| **NSA** | 5 | 43 | 155 | 0.27 |
| **PAR** | 2 | 28 | 120 | 0.23 |
| **PLA** | 87 | 1275 | 4082 | 0.31 |

**Table 9:** Descriptive statistics of network and treatment characteristics for the incidence of CPSP ≤ 6 months (combination agents).

**Figure 9:** Regression plot for the incidence of CPSP ≤ 6 months (combination agents) demonstrating the effect of baseline risk (X axis, proportion of participants in the placebo group with pain) compared with the effect estimate (Y axis, log odds). Each colour represents a different intervention. Demonstrates that as baseline risk increases, the reduction in the incidence of CPSP with the intervention increases (more effective).

**Figure 10:** Covariate plot for the incidence of CPSP ≤ 6 months (combination agents) showing the distribution of baseline risk (Y axis) in each study (X-axis) grouped by treatment (abbreviation above plot). Red dashed line is the mean. Demonstrates that for some combination interventions (LIDKET and KETGLU for example), baseline risk is lower compared to other interventions (KET and GAB). Such imbalance may violate transitivity assumptions and negatively affect the relative efficacy of combination agents if standard network meta-analysis had been performed.

**Figure 11:** SUCRA plot for ranking of treatments at a covariate value of 0.35 (baseline risk) for the incidence of CPSP ≤ 6 months (combination agents). Treatments with lines towards the top left have a higher probability of ranking higher (GABKET) compared to those at the bottom right (PLA).

| **Intervention** | **SUCRA** |
| --- | --- |
| **Gabapentinoids and Ketamine (GABKET)** | **94.8%** |
| **Lidocaine (LID)** | **73.6%** |
| Alpha-2 agonists (ALP) | 65.4% |
| Magnesium (MAG) | 56.2% |
| Nefopam (NEF) | 55.4% |
| Paracetamol (PAR) | 51.3% |
| Gabapentinoids and Glucocorticoids (GABGLU) | 51.2% |
| Lidocaine and Ketamine (LIDKET) | 50.4% |
| NSAIDs (NSA) | 39.6% |
| **Ketamine (KET)** | **39.5%** |
| **Gabapentinoids (GAB)** | **37.1%** |
| Glucocorticoids (GLU) | 35.9% |
| Placebo (PLA) | 13.3% |

**Table 10:** SUCRA scores for each intervention studied for the incidence of CPSP ≤ 6 months (combination agents). The higher the SUCRA %, the higher the probability that the intervention is top ranked. Note that SUCRA does not take account of risk of bias in included studies or the uncertainty of estimates. Only some interventions (highlighted in bold green) demonstrated estimates consistent with a reduction in CPSP despite lower rankings.

**Figure 12:** League heat plot demonstrating relative effects of treatment (X-axis) compared with comparator (Y-axis) at a fixed covariate value of 0.35 for the incidence of CPSP ≤ 6 months (combination agents). Green cells are those consistent with a reduction in pain (darker colour means stronger effect) whilst red interventions are consistent with an increase in pain (darker colour means stronger effect). Those highlighted with a ****** have credible intervals consistent with an effect.

**Figure 13:** Forest plot showing the posterior median odds ratio with 95% CrIs at a fixed covariate value of 0.35 for the incidence of CPSP ≤ 6 months (combination agents). Demonstrates possible reductions with ketamine, gabapentinoids and ketamine, gabapentinoids and lidocaine. X-axis on log scale to aid visualisation due to extreme values. Effects are relative to placebo.

**Diagnostics**

Residual deviance of 198.3 compared with 186 data points.

- The above suggests some problems with model fit
- All PSRF values **<1.0001** (trace and density plots below)

**
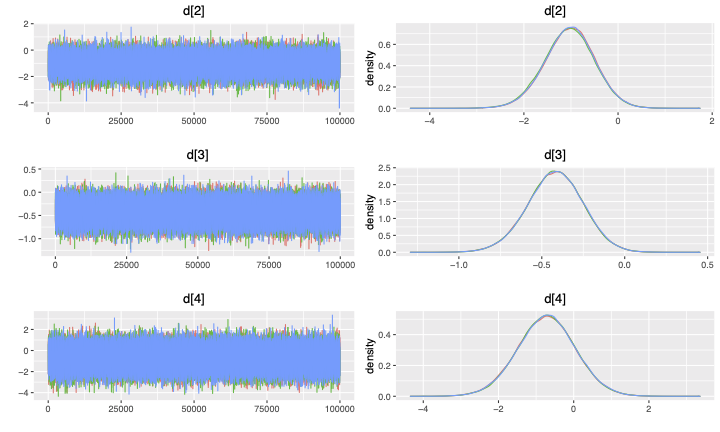
**

**
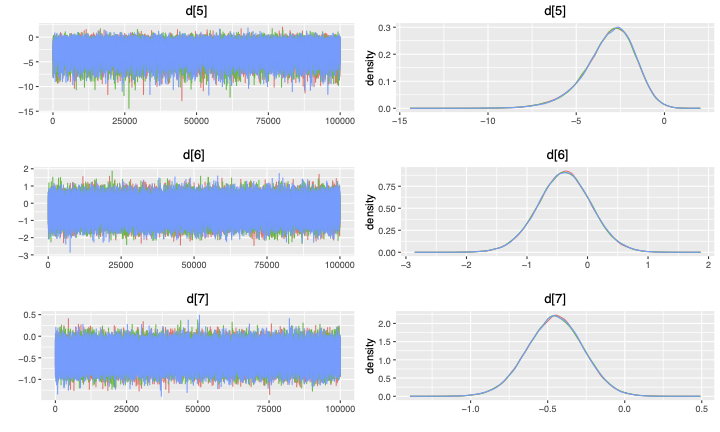
**

**
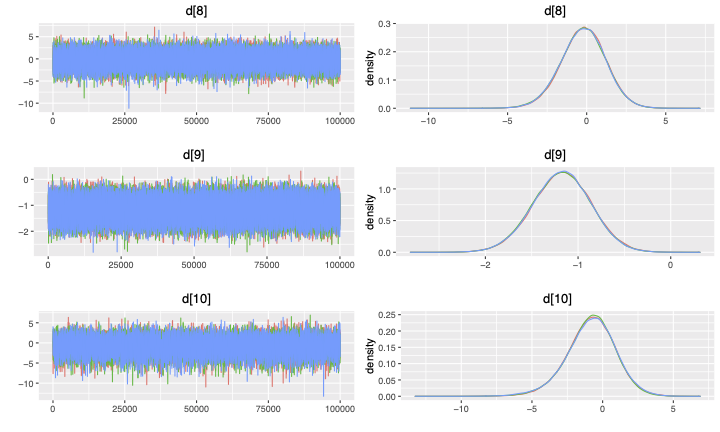
**


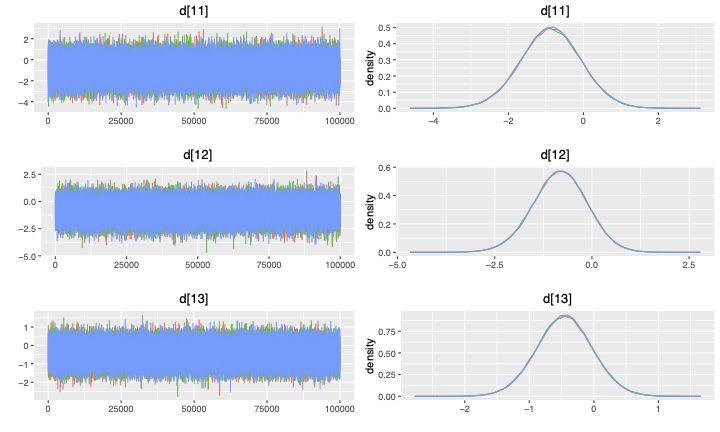


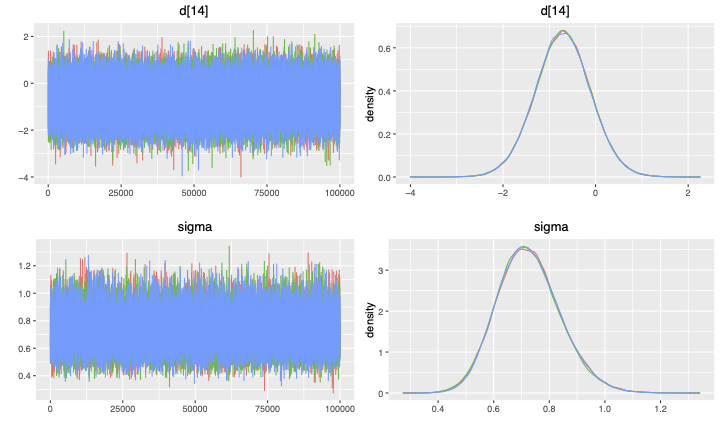


**Inconsistency assessment (no baseline risk adjustment)**

| **Comparison** | **P value** |
| --- | --- |
| GLU-GAB | 0.28 |
| KET-GAB | 0.65 |
| KET-GLU | 0.56 |
| PLA-GLU | 0.48 |
| LID-KET | 0.94 |
| NEF-KET | 0.72 |
| PLA-KET | 0.57 |

**Table 11:** Node-splitting method to assess inconsistency. No evidence of inconsistency although direct estimates were imprecise.

**Sensitivity analysis for incidence of CPSP ≤ 6 months**

**Neuropathic pain only for incidence of CPSP ≤ 6 months**

**
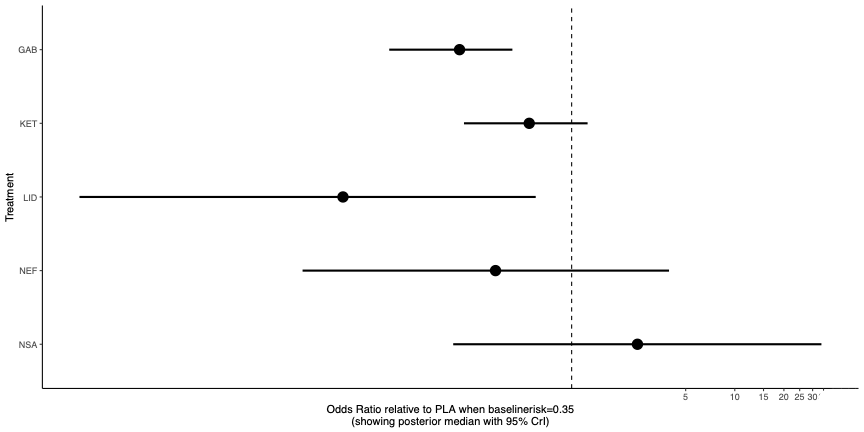
**

**Figure 14:** Forest plot showing the posterior median odds ratio with 95% CrIs at a fixed covariate value of 0.35. Demonstrates possible reductions in the incidence of neuropathic pain at ≤ 6 months with gabapentinoids (OR 0.21; 95% CrI 0.08 to 0.43) and lidocaine (OR 0.04; 95% CrI 0.00 to 0.60). X-axis on log scale to aid visualisation due to extreme values. Effects are relative to placebo.

**Low risk of bias only for incidence of CPSP ≤ 6 months**

**Figure 15:** Forest plot showing the posterior median odds ratio with 95% CrIs at a fixed covariate value of 0.35 in low risk of bias trials only. As only 16 studies included which reduces power, only GABKET showed a reduction in chronic pain (OR 0.04; 95% CrI 0.00 to 0.99). X-axis on log scale to aid visualisation due to extreme values. Effects are relative to placebo.

# **CINeMA assessment for incidence of CPSP ≤ 6 months**

**
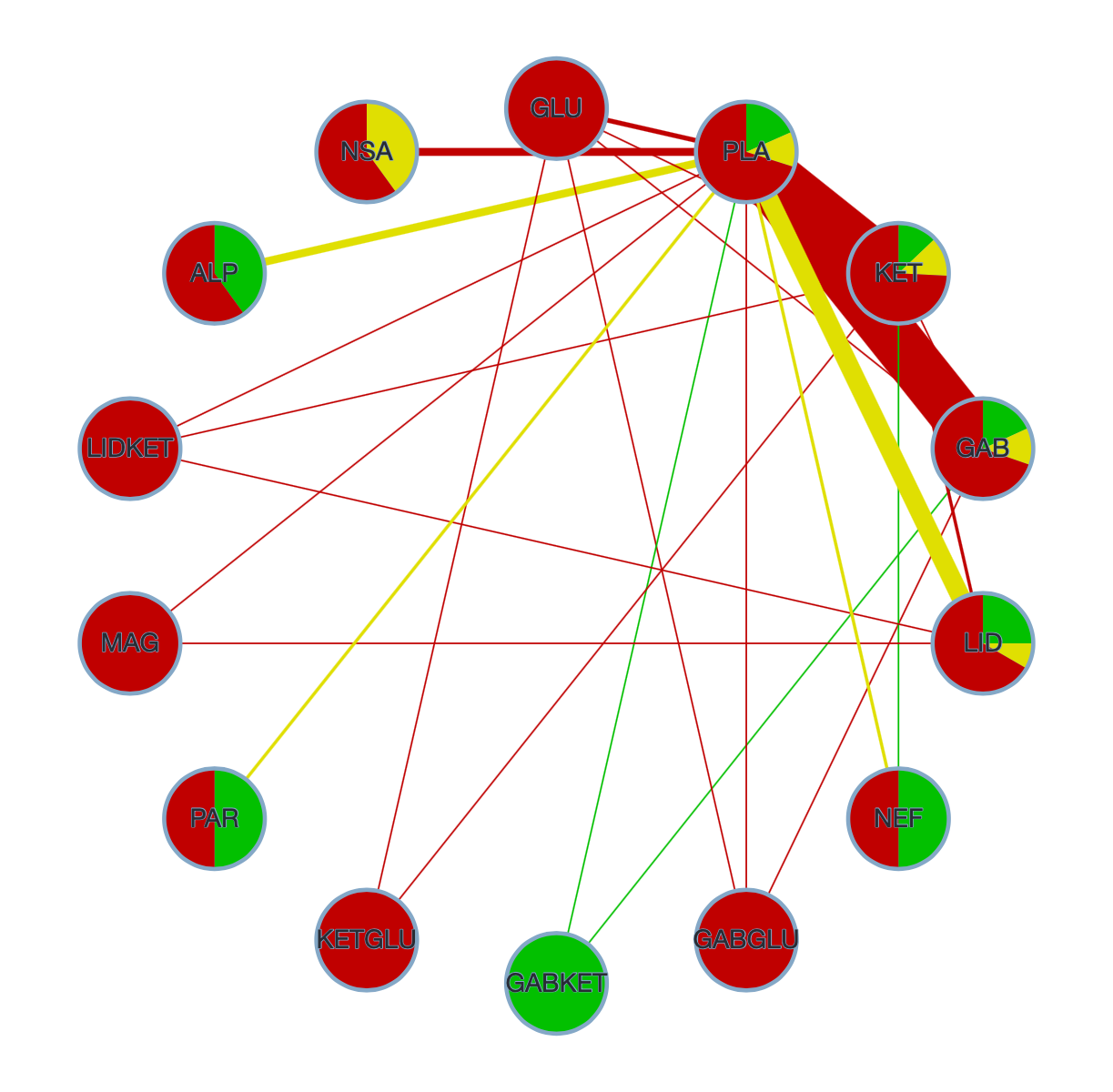
**

**Figure 16:** Network plot from CINeMA for the incidence of CPSP ≤ 6 months. Nodes are equal sizes to aid visualisation. Node colour shows overall risk of bias for each intervention. The colour of edges shows the average risk of bias between comparisons with the thickness being proportional to the number of studies in the comparison. Red is high risk, yellow is unclear risk and green is low risk of bias.

**
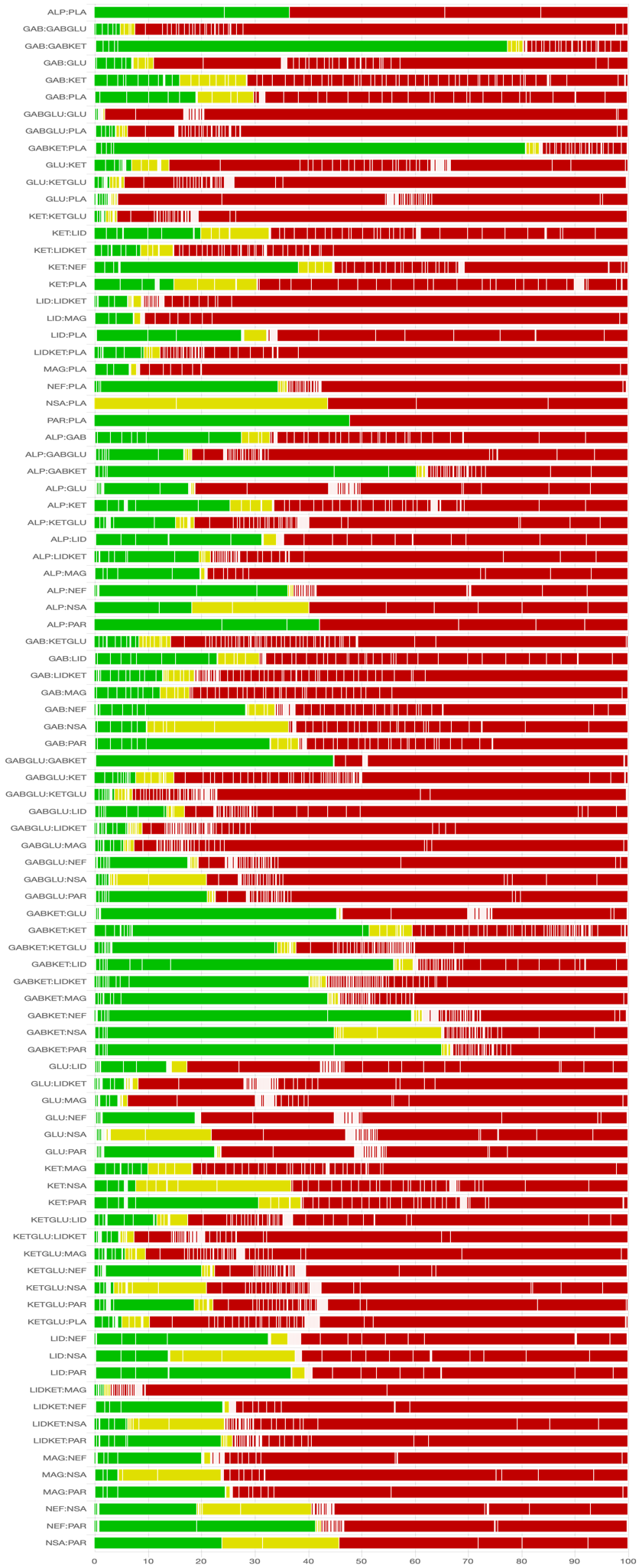
**

**Figure 17:** Contribution matrix for low, unclear and high risk of bias studies per comparison.

| **Comparison** | **N** | **Within-study bias** | **Reporting bias** | **Indirectness** | **Imprecision** | **Heterogeneity** | **Incoherence** | **Confidence** |
| --- | --- | --- | --- | --- | --- | --- | --- | --- |
| ALP-PLA | 5 | Some concerns | Low risk | No concerns | Major concerns | No concerns | No concerns | Very low |
| GAB-GABGLU | 1 | Major concerns | Low risk | No concerns | Major concerns | No concerns | No concerns | Very low |
| GAB-GABKET | 1 | No concerns | Low risk | No concerns | Some concerns | Some concerns | Some concerns | Very low |
| GAB-GLU | 1 | Major concerns | Low risk | No concerns | Major concerns | No concerns | No concerns | Very low |
| GAB-KET | 1 | Major concerns | Low risk | No concerns | Major concerns | No concerns | No concerns | Very low |
| GAB-PLA | 33 | Major concerns | Low risk | No concerns | No concerns | Some concerns | No concerns | Low |
| GABGLU-GLU | 1 | Major concerns | Low risk | No concerns | Major concerns | No concerns | No concerns | Very low |
| GABGLU-PLA | 1 | Major concerns | Low risk | No concerns | Major concerns | No concerns | No concerns | Very low |
| GABKET-PLA | 1 | No concerns | Low risk | No concerns | No concerns | No concerns | Some concerns | Moderate |
| GLU-KET | 1 | Major concerns | Low risk | No concerns | Major concerns | No concerns | No concerns | Very low |
| GLU-KETGLU | 1 | Major concerns | Low risk | No concerns | Major concerns | No concerns | No concerns | Very low |
| GLU-PLA | 3 | Major concerns | Low risk | No concerns | Major concerns | No concerns | No concerns | Very low |
| KET-KETGLU | 1 | Major concerns | Low risk | No concerns | Major concerns | No concerns | No concerns | Very low |
| KET-LID | 2 | Some concerns | Low risk | No concerns | Some concerns | Some concerns | No concerns | Very low |
| KET-LIDKET | 1 | Major concerns | Low risk | No concerns | Major concerns | No concerns | No concerns | Very low |
| KET-NEF | 1 | Some concerns | Low risk | No concerns | Major concerns | No concerns | No concerns | Very low |
| KET-PLA | 30 | Major concerns | Low risk | No concerns | No concerns | Some concerns | No concerns | Low |
| LID-LIDKET | 1 | Major concerns | Low risk | No concerns | Major concerns | No concerns | Some concerns | Very low |
| LID-MAG | 1 | Major concerns | Low risk | No concerns | Major concerns | No concerns | No concerns | Very low |
| LID-PLA | 12 | Some concerns | Low risk | No concerns | No concerns | Some concerns | No concerns | Low |
| LIDKET-PLA | 1 | Major concerns | Low risk | No concerns | Major concerns | No concerns | No concerns | Very low |
| MAG-PLA | 1 | Major concerns | Low risk | No concerns | Major concerns | No concerns | No concerns | Very low |
| NEF-PLA | 2 | Some concerns | Low risk | No concerns | Major concerns | No concerns | No concerns | Very low |
| NSA-PLA | 5 | Major concerns | Low risk | No concerns | Major concerns | No concerns | No concerns | Very low |
| PAR-PLA | 2 | Some concerns | Low risk | No concerns | Major concerns | No concerns | No concerns | Very low |
| ALP-GAB | IND | Some concerns | Low risk | No concerns | Major concerns | No concerns | No concerns | Very low |
| ALP-GABGLU | IND | Major concerns | Low risk | No concerns | Major concerns | No concerns | No concerns | Very low |
| ALP-GABKET | IND | Some concerns | Low risk | No concerns | Major concerns | No concerns | No concerns | Very low |
| ALP-GLU | IND | Major concerns | Low risk | No concerns | Major concerns | No concerns | No concerns | Very low |
| ALP-KET | IND | Some concerns | Low risk | No concerns | Major concerns | No concerns | No concerns | Very low |
| ALP-KETGLU | IND | Major concerns | Low risk | No concerns | Major concerns | No concerns | No concerns | Very low |
| ALP-LID | IND | Some concerns | Low risk | No concerns | Major concerns | No concerns | No concerns | Very low |
| ALP-LIDKET | IND | Major concerns | Low risk | No concerns | Major concerns | No concerns | No concerns | Very low |
| ALP-MAG | IND | Major concerns | Low risk | No concerns | Major concerns | No concerns | No concerns | Very low |
| ALP-NEF | IND | Some concerns | Low risk | No concerns | Major concerns | No concerns | No concerns | Very low |
| ALP-NSA | IND | Some concerns | Low risk | No concerns | Major concerns | No concerns | No concerns | Very low |
| ALP-PAR | IND | Some concerns | Low risk | No concerns | Major concerns | No concerns | No concerns | Very low |
| GAB-KETGLU | IND | Major concerns | Low risk | No concerns | Major concerns | No concerns | No concerns | Very low |
| GAB-LID | IND | Some concerns | Low risk | No concerns | Some concerns | Some concerns | No concerns | Very low |
| GAB-LIDKET | IND | Major concerns | Low risk | No concerns | Major concerns | No concerns | No concerns | Very low |
| GAB-MAG | IND | Major concerns | Low risk | No concerns | Major concerns | No concerns | No concerns | Very low |
| GAB-NEF | IND | Some concerns | Low risk | No concerns | Major concerns | No concerns | No concerns | Very low |
| GAB-NSA | IND | Major concerns | Low risk | No concerns | Major concerns | No concerns | No concerns | Very low |
| GAB-PAR | IND | Some concerns | Low risk | No concerns | Major concerns | No concerns | No concerns | Very low |
| GABGLU-GABKET | IND | Some concerns | Low risk | No concerns | Major concerns | No concerns | No concerns | Very low |
| GABGLU-KET | IND | Major concerns | Low risk | No concerns | Major concerns | No concerns | No concerns | Very low |
| GABGLU-KETGLU | IND | Major concerns | Low risk | No concerns | Major concerns | No concerns | No concerns | Very low |
| GABGLU-LID | IND | Major concerns | Low risk | No concerns | Major concerns | No concerns | No concerns | Very low |
| GABGLU-LIDKET | IND | Major concerns | Low risk | No concerns | Major concerns | No concerns | No concerns | Very low |
| GABGLU-MAG | IND | Major concerns | Low risk | No concerns | Major concerns | No concerns | No concerns | Very low |
| GABGLU-NEF | IND | Major concerns | Low risk | No concerns | Major concerns | No concerns | No concerns | Very low |
| GABGLU-NSA | IND | Major concerns | Low risk | No concerns | Major concerns | No concerns | No concerns | Very low |
| GABGLU-PAR | IND | Major concerns | Low risk | No concerns | Major concerns | No concerns | No concerns | Very low |
| GABKET-GLU | IND | Some concerns | Low risk | No concerns | Some concerns | Some concerns | No concerns | Very low |
| GABKET-KET | IND | Some concerns | Low risk | No concerns | Some concerns | Some concerns | No concerns | Very low |
| GABKET-KETGLU | IND | Some concerns | Low risk | No concerns | Major concerns | No concerns | No concerns | Very low |
| GABKET-LID | IND | Some concerns | Low risk | No concerns | Major concerns | No concerns | No concerns | Very low |
| GABKET-LIDKET | IND | Some concerns | Low risk | No concerns | Major concerns | No concerns | No concerns | Very low |
| GABKET-MAG | IND | Some concerns | Low risk | No concerns | Major concerns | No concerns | No concerns | Very low |
| GABKET-NEF | IND | Some concerns | Low risk | No concerns | Major concerns | No concerns | No concerns | Very low |
| GABKET-NSA | IND | Some concerns | Low risk | No concerns | Major concerns | No concerns | No concerns | Very low |
| GABKET-PAR | IND | Some concerns | Low risk | No concerns | Major concerns | No concerns | No concerns | Very low |
| GLU-LID | IND | Major concerns | Low risk | No concerns | Major concerns | No concerns | No concerns | Very low |
| GLU-LIDKET | IND | Major concerns | Low risk | No concerns | Major concerns | No concerns | No concerns | Very low |
| GLU-MAG | IND | Major concerns | Low risk | No concerns | Major concerns | No concerns | No concerns | Very low |
| GLU-NEF | IND | Major concerns | Low risk | No concerns | Major concerns | No concerns | No concerns | Very low |
| GLU-NSA | IND | Major concerns | Low risk | No concerns | Major concerns | No concerns | No concerns | Very low |
| GLU-PAR | IND | Major concerns | Low risk | No concerns | Major concerns | No concerns | No concerns | Very low |
| KET-MAG | IND | Major concerns | Low risk | No concerns | Major concerns | No concerns | No concerns | Very low |
| KET-NSA | IND | Major concerns | Low risk | No concerns | Major concerns | No concerns | No concerns | Very low |
| KET-PAR | IND | Some concerns | Low risk | No concerns | Major concerns | No concerns | No concerns | Very low |
| KETGLU-LID | IND | Major concerns | Low risk | No concerns | Major concerns | No concerns | No concerns | Very low |
| KETGLU-LIDKET | IND | Major concerns | Low risk | No concerns | Major concerns | No concerns | No concerns | Very low |
| KETGLU-MAG | IND | Major concerns | Low risk | No concerns | Major concerns | No concerns | No concerns | Very low |
| KETGLU-NEF | IND | Major concerns | Low risk | No concerns | Major concerns | No concerns | No concerns | Very low |
| KETGLU-NSA | IND | Major concerns | Low risk | No concerns | Major concerns | No concerns | No concerns | Very low |
| KETGLU-PAR | IND | Major concerns | Low risk | No concerns | Major concerns | No concerns | No concerns | Very low |
| KETGLU-PLA | IND | Major concerns | Low risk | No concerns | Major concerns | No concerns | No concerns | Very low |
| LID-NEF | IND | Some concerns | Low risk | No concerns | Major concerns | No concerns | No concerns | Very low |
| LID-NSA | IND | Some concerns | Low risk | No concerns | Major concerns | No concerns | No concerns | Very low |
| LID-PAR | IND | Some concerns | Low risk | No concerns | Major concerns | No concerns | No concerns | Very low |
| LIDKET-MAG | IND | Major concerns | Low risk | No concerns | Major concerns | No concerns | No concerns | Very low |
| LIDKET-NEF | IND | Major concerns | Low risk | No concerns | Major concerns | No concerns | No concerns | Very low |
| LIDKET-NSA | IND | Major concerns | Low risk | No concerns | Major concerns | No concerns | No concerns | Very low |
| LIDKET-PAR | IND | Major concerns | Low risk | No concerns | Major concerns | No concerns | No concerns | Very low |
| MAG-NEF | IND | Major concerns | Low risk | No concerns | Major concerns | No concerns | No concerns | Very low |
| MAG-NSA | IND | Major concerns | Low risk | No concerns | Major concerns | No concerns | No concerns | Very low |
| MAG-PAR | IND | Some concerns | Low risk | No concerns | Major concerns | No concerns | No concerns | Very low |
| NEF-NSA | IND | Some concerns | Low risk | No concerns | Major concerns | No concerns | No concerns | Very low |
| NEF-PAR | IND | Some concerns | Low risk | No concerns | Major concerns | No concerns | No concerns | Very low |
| NSA-PAR | IND | Some concerns | Low risk | No concerns | Major concerns | No concerns | No concerns | Very low |

**Table 12:** Overall assessment of comparisons from CINeMA for the incidence of CPSP ≤ 6 months. All evidence low to very low due to issues with risk of bias and imprecision. IND: indirect evidence.

# **Incidence of CPSP > 6 months (single agents)**

**Network meta-regression results**

**Figure 18:** Network plot for the incidence of CPSP > 6 months (single agents). Node size (black) is proportional to the number of studies evaluating that intervention and the grey lines the number of comparisons between each treatment.

| **Number of Interventions** | 7 |
| --- | --- |
| **Number of Studies** | 16 |
| **Total Number of Patients in Network** | 1613 |
| **Total Possible Pairwise Comparisons** | 21 |
| **Total Number of Pairwise Comparisons With Direct Data** | 8 |
| **Is the network connected?** | Yes |
| **Number of Two-arm Studies** | 14 |
| **Number of Multi-Arms Studies** | 2 |
| **Total Number of Events in Network** | 201 |
| **Number of Studies With No Zero Events** | 13 |
| **Number of Studies With At Least One Zero Event** | 3 |
| **Number of Studies with All Zero Events** | 0 |

| **Treatment** | **Studies** | **Events** | **Participants** | **Average proportion with CPSP** |
| --- | --- | --- | --- | --- |
| **ALP** | 1 | 1 | 36 | 0.03 |
| **GAB** | 5 | 35 | 355 | 0.1 |
| **GLU** | 1 | 9 | 56 | 0.16 |
| **KET** | 6 | 28 | 279 | 0.1 |
| **NEF** | 1 | 3 | 22 | 0.14 |
| **NSA** | 4 | 11 | 149 | 0.07 |
| **PLA** | 16 | 114 | 716 | 0.16 |

**Table 13:** Descriptive statistics of network and treatment characteristics for the incidence of CPSP > 6 months (single agents).

**Figure 19:** Regression plot for the incidence of CPSP > 6 months (single agents) demonstrating the effect of baseline risk (X axis, proportion of participants in the placebo group with pain) compared with the effect estimate (Y axis, log odds). Each colour represents a different intervention. Demonstrates that as baseline risk increases, the reduction in the incidence of CPSP with the intervention increases (more effective).

**Figure 20:** Covariate plot for the incidence of CPSP > 6 months (single agents) showing the distribution of baseline risk (Y axis) in each study (X-axis) grouped by treatment (abbreviation above plot). Red dashed line is the mean. As NSA and NEF have some studies with relatively higher baseline risk, such imbalance may violate transitivity assumptions if standard network meta-analysis was performed.

**Figure 21:** SUCRA plot for ranking of treatments at a covariate value of 0.2 (baseline risk) for the incidence of CPSP > 6 months (single agents). Treatments with lines towards the top left have a higher probability of ranking higher (ALP) compared to those at the bottom right (PLA).

| **Intervention** | **SUCRA** |
| --- | --- |
| Alpha-2 agonists | 90.1% |
| **NSAIDs** | **75.4%** |
| Gabapentinoids | 50.1% |
| Nefopam | 44.3% |
| Ketamine | 41.2% |
| Glucocorticoids | 32.4% |
| Placebo | 15.7% |

**Table 14:** SUCRA scores for each intervention studied. The higher the SUCRA %, the higher the probability that the intervention is top ranked. Note that SUCRA does not take account of risk of bias in included studies or uncertainty of estimates. Only one intervention (highlighted in bold green) demonstrated estimates consistent with a reduction in CPSP.

**Figure 22:** League heat plot for the incidence of CPSP > 6 months (single agents) demonstrating relative effects of treatment (X-axis) compared with comparator (Y-axis) at a fixed covariate value of 0.2. Green cells are those consistent with a reduction in pain (darker colour means stronger effect) whilst red interventions are consistent with an increase in pain (darker colour means stronger effect). Those highlighted with a ****** have credible intervals consistent with an effect (NSA).

**
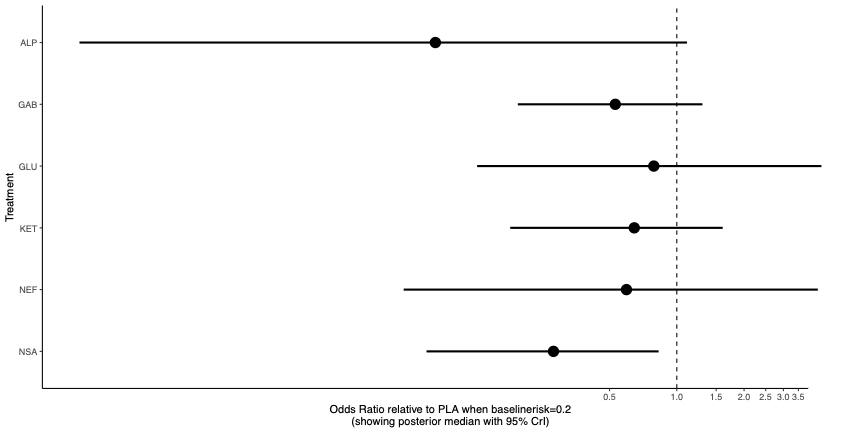
**

**Figure 23:** Forest plot showing the posterior median odds ratio with 95% CrIs at a fixed covariate value of 0.2 for the incidence of CPSP > 6 months (single agents). Demonstrates possible reductions in the incidence of pain with NSAIDs. X-axis is on a log scale to aid visualisation due to extreme values. Effects are relative to placebo.

**Diagnostics**

Residual deviance of 37 compared with 34 data points.

- All PSRF values **<1.0001** (trace and density plots below)

**
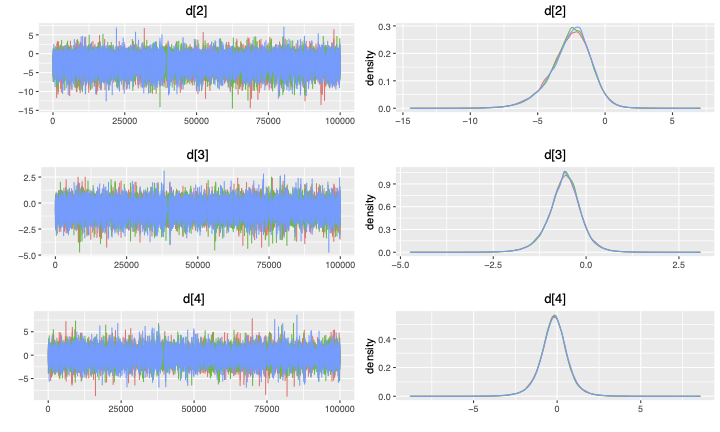
**

**
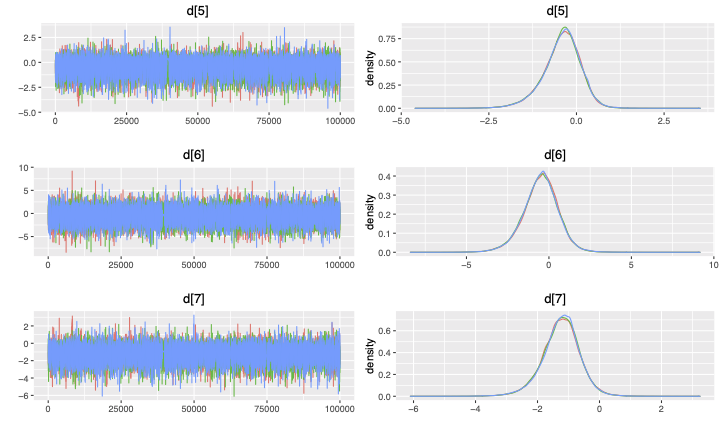
**

**
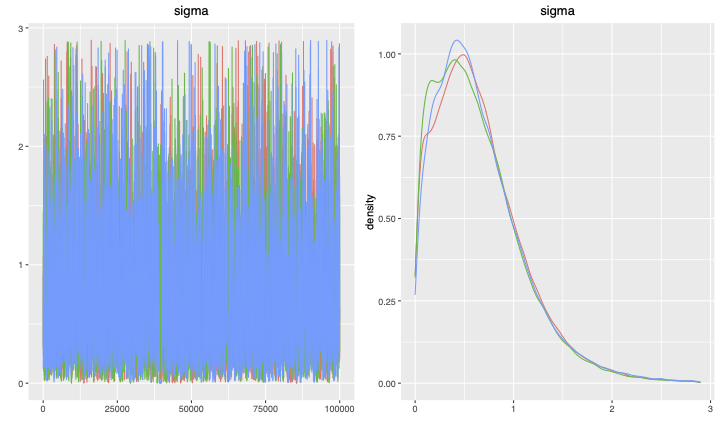
**

**Inconsistency assessment (no baseline risk adjustment)**

Cannot be performed for this outcome due to network characteristics.

**Sensitivity analysis**

**Neuropathic pain for incidence of CPSP > 6 months**

**Figure 24:** Forest plot showing the posterior median odds ratio with 95% CrIs at a fixed covariate value of 0.2 in trials evaluating neuropathic pain outcomes (2 studies) for the incidence of CPSP > 6 months (single agents). No intervention demonstrates any benefit although estimates were imprecise. X-axis is on a log scale to aid visualisation due to extreme values. Effects are relative to placebo.

**Low risk of bias only for incidence of CPSP > 6 months**

**Figure 25:** Forest plot showing the posterior median odds ratio with 95% CrIs at a fixed covariate value of 0.2 in low risk of bias trials only for the incidence of CPSP > 6 months (single agents). No intervention shows any benefit. Effects are relative to placebo.

# **CINeMA assessment for incidence of CPSP > 6 months**

**
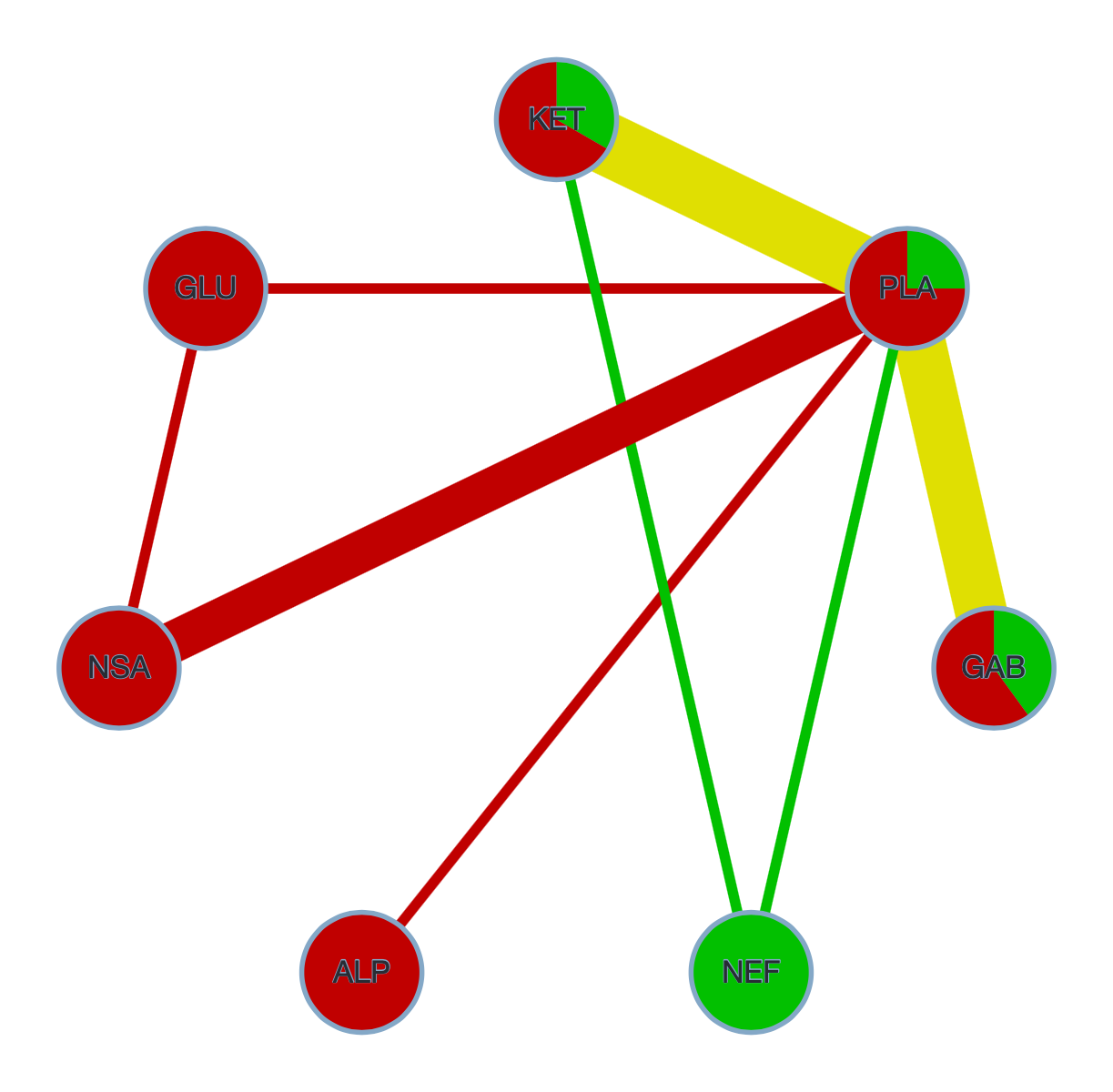
**

**Figure 26:** Network plot from CINeMA for the incidence of CPSP > 6 months (single agents). Nodes are equal sizes to aid visualisation. Node colour shows overall risk of bias for each intervention. The colour of edges shows the average risk of bias between comparisons with thickness being the number of studies in the comparison. Red is high risk, yellow is unclear risk and green is low risk.

**
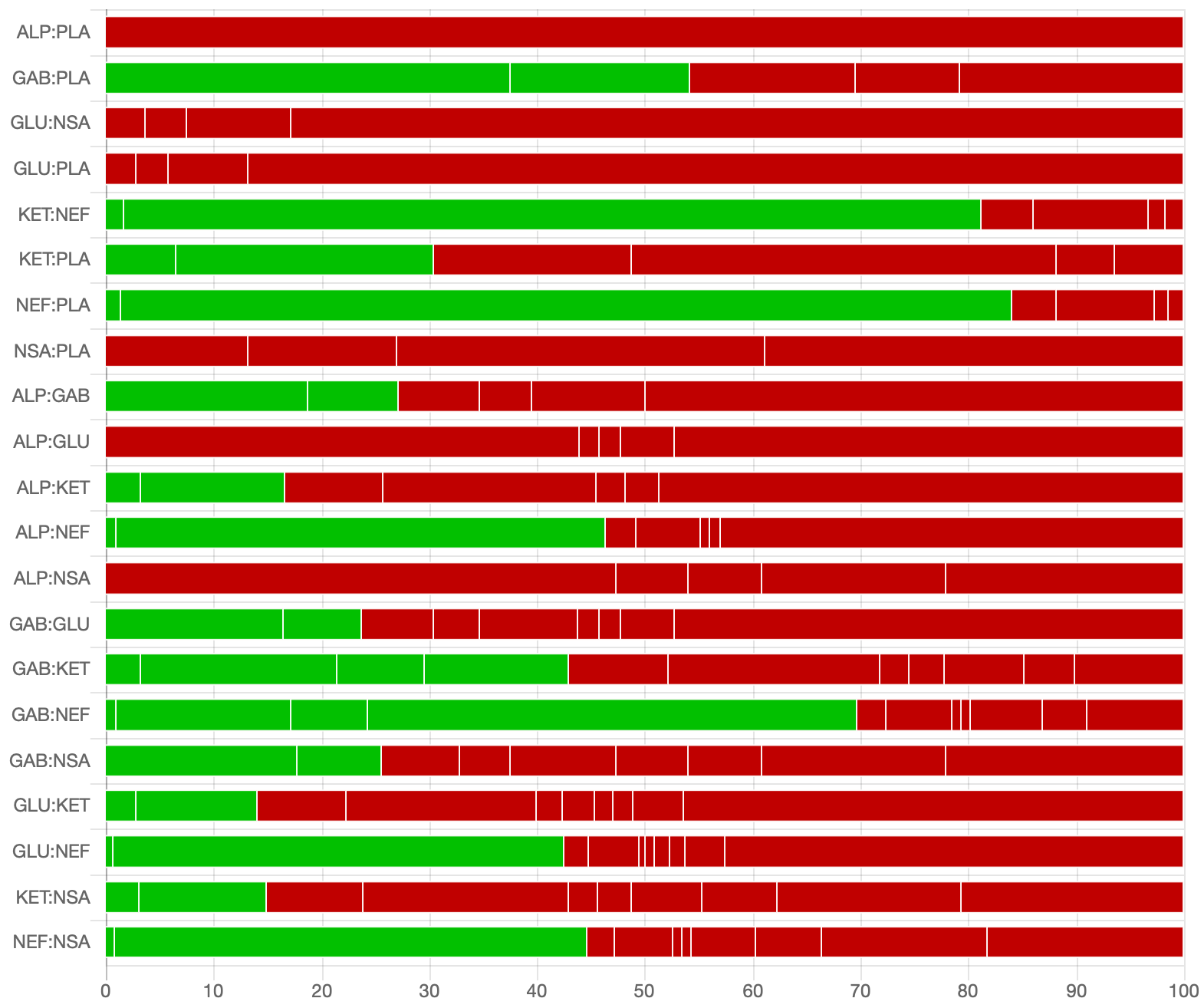
**

**Figure 27:** Contribution matrix for low, unclear and high risk of bias studies per comparison.

| **Comparison** | **N** | **Within-study bias** | **Reporting bias** | **Indirectness** | **Imprecision** | **Heterogeneity** | **Incoherence** | **Confidence** |
| --- | --- | --- | --- | --- | --- | --- | --- | --- |
| ALP-PLA | 1 | Major concerns | Low risk | No concerns | Major concerns | No concerns | No concerns | Very low |
| GAB-PLA | 5 | Some concerns | Low risk | No concerns | Major concerns | No concerns | No concerns | Very low |
| GLU-NSA | 1 | Major concerns | Low risk | No concerns | Some concerns | Some concerns | No concerns | Very low |
| GLU-PLA | 1 | Major concerns | Low risk | No concerns | Major concerns | No concerns | No concerns | Very low |
| KET-NEF | 1 | No concerns | Low risk | No concerns | Major concerns | No concerns | No concerns | Low |
| KET-PLA | 6 | Some concerns | Low risk | No concerns | Major concerns | No concerns | No concerns | Very low |
| NEF-PLA | 1 | No concerns | Low risk | No concerns | Major concerns | No concerns | No concerns | Low |
| NSA-PLA | 4 | Major concerns | Low risk | No concerns | No concerns | Some concerns | No concerns | Low |
| ALP-GAB | IND | Some concerns | Low risk | No concerns | Major concerns | No concerns | No concerns | Very low |
| ALP-GLU | IND | Major concerns | Low risk | No concerns | Major concerns | No concerns | No concerns | Very low |
| ALP-KET | IND | Major concerns | Low risk | No concerns | Major concerns | No concerns | No concerns | Very low |
| ALP-NEF | IND | Some concerns | Low risk | No concerns | Major concerns | No concerns | No concerns | Very low |
| ALP-NSA | IND | Major concerns | Low risk | No concerns | Major concerns | No concerns | No concerns | Very low |
| GAB-GLU | IND | Major concerns | Low risk | No concerns | Major concerns | No concerns | No concerns | Very low |
| GAB-KET | IND | Some concerns | Low risk | No concerns | Major concerns | No concerns | No concerns | Very low |
| GAB-NEF | IND | Some concerns | Low risk | No concerns | Major concerns | No concerns | No concerns | Very low |
| GAB-NSA | IND | Some concerns | Low risk | No concerns | Some concerns | Some concerns | No concerns | Very low |
| GLU-KET | IND | Major concerns | Low risk | No concerns | Major concerns | No concerns | No concerns | Very low |
| GLU-NEF | IND | Some concerns | Low risk | No concerns | Major concerns | No concerns | No concerns | Very low |
| KET-NSA | IND | Major concerns | Low risk | No concerns | Some concerns | Some concerns | No concerns | Very low |
| NEF-NSA | IND | Some concerns | Low risk | No concerns | Major concerns | No concerns | No concerns | Very low |

**Table 15:** Overall assessment of comparisons from CINeMA for the incidence of CPSP > 6 months (single agents). All evidence low to very low due to issues with risk of bias and imprecision. IND: indirect evidence.

# **Severity of CPSP ≤ 6 months (single agents)**

**Network meta-regression results**

**Figure 28:** Network plot for the severity of CPSP ≤ 6 months (single agents). Node size (black) is proportional to the number of studies evaluating that intervention and the grey lines the number of comparisons between each treatment.

| **Number of Interventions** | 9 |
| --- | --- |
| **Number of Studies** | 65 |
| **Total Number of Patients in Network** | 4996 |
| **Total Possible Pairwise Comparisons** | 36 |
| **Total Number of Pairwise Comparisons With Direct Data** | 14 |
| **Is the network connected?** | Yes |
| **Number of Two-arm Studies** | 61 |
| **Number of Multi-Arms Studies** | 4 |
| **Average outcome** | 1.39 |

| **Treatment** | **Studies** | **Participants** | **Average pain score** |
| --- | --- | --- | --- |
| **ALP** | 1 | 34 | 2.50 |
| **GAB** | 27 | 1063 | 1.16 |
| **GLU** | 6 | 306 | 1.84 |
| **KET** | 22 | 809 | 1.52 |
| **LID** | 5 | 150 | 0.46 |
| **NEF** | 3 | 92 | 0.77 |
| **NSA** | 5 | 150 | 1.26 |
| **PAR** | 2 | 120 | 0.58 |
| **PLA** | 63 | 2272 | 1.52 |

**Table 16:** Descriptive statistics of network and treatment characteristics for the severity of CPSP ≤ 6 months (single agents).

**Figure 29:** Regression plot for the severity of CPSP ≤ 6 months (single agents) demonstrating the effect of baseline risk (X axis, mean control group pain score) compared with the effect estimate (Y axis, mean difference). Each colour represents a different intervention. Demonstrates that as baseline risk increases, the reduction in the severity of CPSP with the intervention increases (more effective).

**Figure 30:** Covariate plot for the severity of CPSP ≤ 6 months (single agents) showing the distribution of baseline risk (Y-axis) in each study (X-axis) grouped by treatment (abbreviation above plot). Red dashed line is the mean. LID and PAR contain low baseline risk trials which could violate transitivity if standard network meta-analysis was performed.

**Figure 31:** SUCRA plot for ranking of treatments at a covariate value of 2 (baseline risk) for the severity of CPSP ≤ 6 months (single agents). Treatments with lines towards the top left have a higher probability of ranking higher (LID) compared to those at the bottom right (PLA and GLU).

| **Intervention** | **SUCRA** |
| --- | --- |
| **Lidocaine (LID)** | **83.8%** |
| **Gabapentinoids (GAB)** | **76.5%** |
| Nefopam (NEF) | 54.4% |
| **Ketamine (KET)** | **53.3%** |
| Alpha-2 agonists (ALP) | 53% |
| Paracetamol (PAR) | 52.3% |
| NSAIDs (NSA) | 50.7% |
| Placebo (PLA) | 20.3% |
| Glucocorticoids (GLU) | 5.8% |

**Table 17:** SUCRA scores for each intervention for the severity of CPSP ≤ 6 months (single agents). The higher the SUCRA %, the higher the probability that the intervention is top ranked. Note that SUCRA does not take account of risk of bias in included studies or uncertainty of estimates. Interventions are highlighted in bold green if estimates are consistent with a reduction in the severity of CPSP.

**Figure 32:** League heat plot demonstrating the effects of treatment (X-axis) compared with comparator (Y-axis) at a fixed covariate value of 2 for the severity of CPSP ≤ 6 months (single agents). Green cells are those consistent with a reduction in pain (darker colour means stronger effect) whilst red interventions are consistent with an increase in pain (darker colour means stronger effect). Those highlighted with a ****** have credible intervals consistent with an effect.

**Figure 33:** Forest plot showing the posterior median mean difference with 95% CrIs at a fixed covariate value of 2 for the severity of CPSP ≤ 6 months (single agents). X-axis is on a linear scale. GAB, KET and LID demonstrate estimates consistent with a reduction in a severity of pain although lidocaine estimates were imprecise. Effects are relative to placebo.

**Diagnostics**

Residual deviance of 136 compared with 134 data points.

- All PSRF values **<1.002** (trace and density plots below)

**
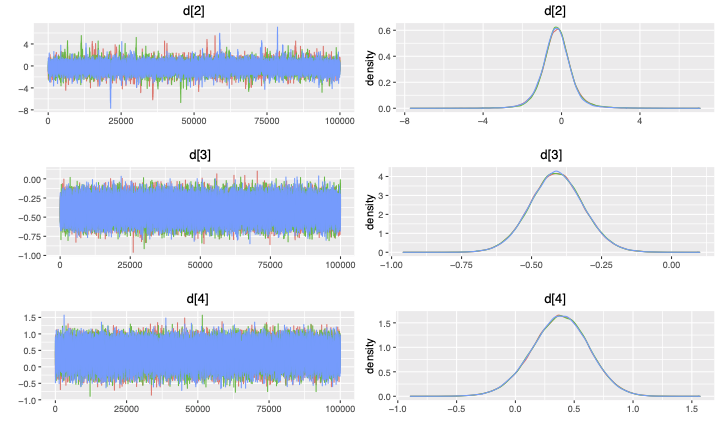
**

**
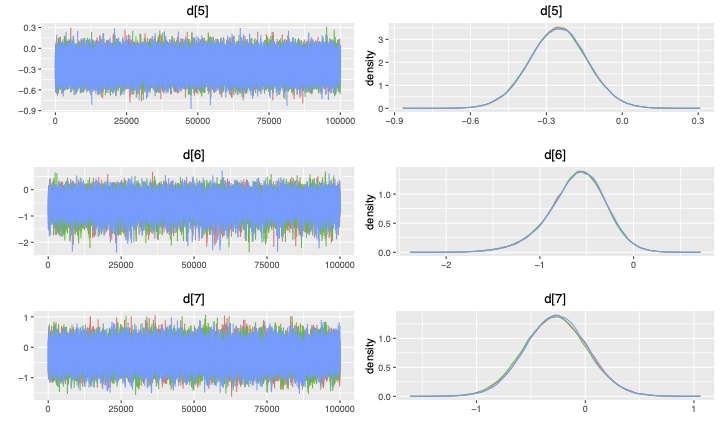
**

**
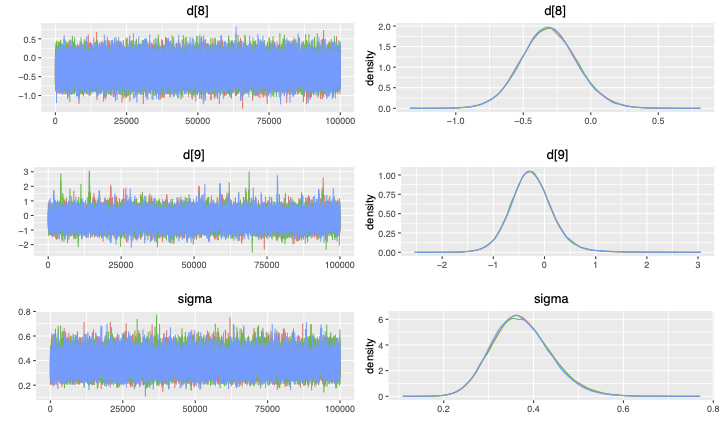
**

**Inconsistency assessment (no baseline risk adjustment)**

| **Comparison** | **P value** |
| --- | --- |
| GAB-GLU | 0.5 |
| GAB-KET | 0.12 |
| GAB-NSA | **<0.001** |
| GAB-PLA | **<0.001** |
| GLU-KET | **0.006** |
| GLU-PLA | **0.009** |
| KET-LID | 0.36 |
| KET-NEF | 0.99 |
| KET-PLA | **0.006** |
| NSA-PLA | **<0.001** |

**Table 18:** Node-splitting method to assess inconsistency. Evidence of inconsistency for many comparisons. Note that the above analysis does not take account of baseline risk and therefore we expected to observe such inconsistency. This further illustrates that transitivity may be violated with standard network meta-analysis.

# **Severity of CPSP ≤ 6 months (combination agents)**

**Network meta-regression results**

**Figure 34:** Network plot for severity of CPSP ≤ 6 months (combination agents). Node size (black) is proportional to the number of studies evaluating that intervention and the grey lines the number of comparisons between each treatment.

| **Number of Interventions** | 12 |
| --- | --- |
| **Number of Studies** | 65 |
| **Total Number of Patients in Network** | 5088 |
| **Total Possible Pairwise Comparisons** | 66 |
| **Total Number of Pairwise Comparisons With Direct Data** | 22 |
| **Is the network connected?** | Yes |
| **Number of Two-arm Studies** | 59 |
| **Number of Multi-Arms Studies** | 6 |
| **Average outcome** | 1.38 |

| **Treatment** | **Studies** | **Participants** | **Average pain score** |
| --- | --- | --- | --- |
| **ALP** | 1 | 34 | 2.5 |
| **GAB** | 27 | 1063 | 1.16 |
| **GABGLU** | 2 | 47 | 0 |
| **GLU** | 6 | 306 | 1.84 |
| **KET** | 22 | 809 | 1.52 |
| **KETGLU** | 1 | 16 | 1 |
| **LID** | 5 | 150 | 0.46 |
| **LIDKET** | 1 | 29 | 1 |
| **NEF** | 3 | 92 | 0.77 |
| **NSA** | 5 | 150 | 1.26 |
| **PAR** | 2 | 120 | 0.58 |
| **PLA** | 63 | 2272 | 1.52 |

**Table 19:** Descriptive statistics of network and treatment characteristics for the severity of CPSP ≤ 6 months (combination agents).

**Figure 35:** Regression plot for the severity of CPSP ≤ 6 months (combination agents) demonstrating the effect of baseline risk (X axis, mean control group pain score) compared with the effect estimate (Y axis, mean difference). Each colour represents a different intervention. Demonstrates that as baseline risk increases the reduction in the severity of pain increases (efficacy improves).

**Figure 36:** Covariate plot for the severity of CPSP ≤ 6 months (combination agents) showing the distribution of baseline risk (Y-axis) in each study (X-axis) grouped by treatment (abbreviation above plot). Red dashed line is the mean. LID and PAR contain low baseline risk trials which could violate transitivity if analysed using standard network meta-analysis.

**Figure 37:** SUCRA plot for ranking of treatments at a covariate value of 2 (baseline risk) for the severity of CPSP ≤ 6 months (combination agents). Treatments with lines towards the top left have a higher probability of ranking higher (LID and GAB) compared to those at the bottom right (PLA and GLU).

| **Intervention** | **SUCRA** |
| --- | --- |
| **Lidocaine (LID)** | **83.3%** |
| **Gabapentinoids (GAB)** | **76.4%** |
| Nefopam (NEF) | 56.2% |
| **Ketamine (KET)** | **55.7%** |
| Gabapentinoids and Glucocorticoids (GABGLU) | 55.2% |
| Alpha-2 agonists (ALP) | 55% |
| Paracetamol (PAR) | 54.9% |
| NSAIDs (NSA) | 53.1% |
| Ketamine and Glucocorticoids (KETGLU) | 49.5% |
| Lidocaine and Ketamine (LIDKET) | 25.6% |
| Placebo (PLA) | 24.8% |
| Glucocorticoids (GLU) | 10.4% |

**Table 20:** SUCRA scores for each intervention studied for the severity of CPSP ≤ 6 months (combination agents). The higher the SUCRA %, the higher the probability that the intervention is top ranked. Note that SUCRA does not take account of risk of bias in included studies or uncertainty of estimates. Interventions are highlighted in bold green if estimates are consistent with a reduction in the severity of pain (LID, GAB and KET).

**Figure 38:** League heat plot for the severity of CPSP ≤ 6 months (combination agents) demonstrating the effects of treatment (X-axis) compared with comparator (Y-axis) at a fixed covariate value of 2. Green cells are those consistent with a reduction in pain (darker colour means stronger effect) whilst red interventions are consistent with an increase in pain (darker colour means stronger effect). Those highlighted with a ****** have credible intervals consistent with an effect.

**Figure 39:** Forest plot showing the posterior median mean difference with 95% CrIs at a fixed covariate value of 2 for the severity of CPSP ≤ 6 months (combination agents). X-axis is on a linear scale. Effects are relative to placebo.

**Diagnostics**

Residual deviance of 139 compared with 138 data points.

- All PSRF values **<1.008** (trace and density plots below)

**
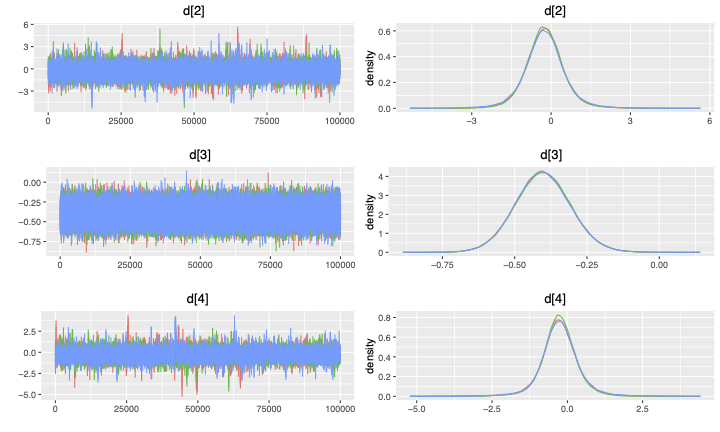
**

**
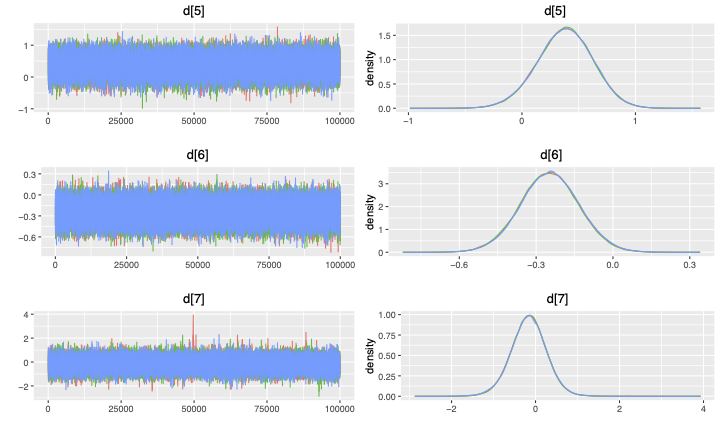
**

**
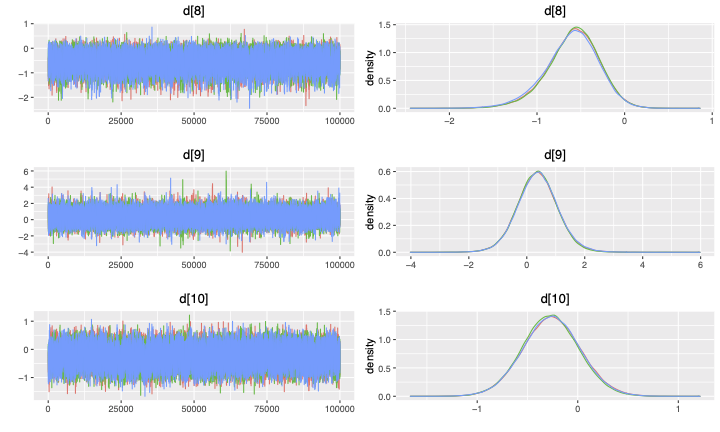
**

**
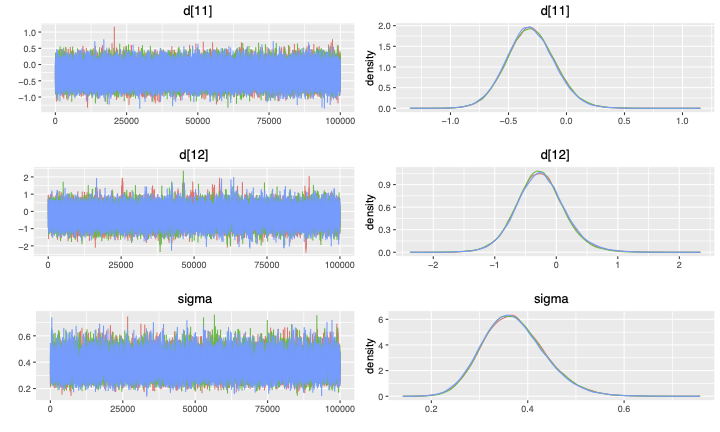
**

**Inconsistency assessment (no baseline risk adjustment)**

| **Comparison** | **P value** |
| --- | --- |
| GAB-GLU | 0.49 |
| GAB-KET | 0.12 |
| GAB-NSA | **<0.001** |
| GAB-PLA | **<0.001** |
| GABGLU-GLU | 0.75 |
| GLU-KET | **0.007** |
| GLU-PLA | **0.008** |
| KET-LID | 0.36 |
| KET-NEF | 0.99 |
| KET-PLA | **0.007** |
| NSA-PLA | **<0.001** |

**Table 21:** Node-splitting method to assess inconsistency for the severity of CPSP ≤ 6 months (combination agents). Evidence of inconsistency for many comparisons. Note that the above analysis does not take account of baseline risk and therefore we expected to observe such inconsistency. This further illustrates that transitivity may be violated with standard network meta-analysis.

**Sensitivity analysis**

**Low risk of bias only for severity of CPSP ≤ 6 months**

**Figure 40:** Forest plot showing the posterior median mean difference with 95% CrIs at a fixed covariate value of 2 in low risk of bias trials only (6 trials) for the severity of CPSP ≤ 6 months. Estimates for gabapentinoids consistent with reduction in severity of pain (MD -0.63; 95% CrI -1.26 to -0.02). X-axis is on a linear scale. Effects are relative to placebo.

# **CINeMA assessment for severity of CPSP ≤ 6 months**

**
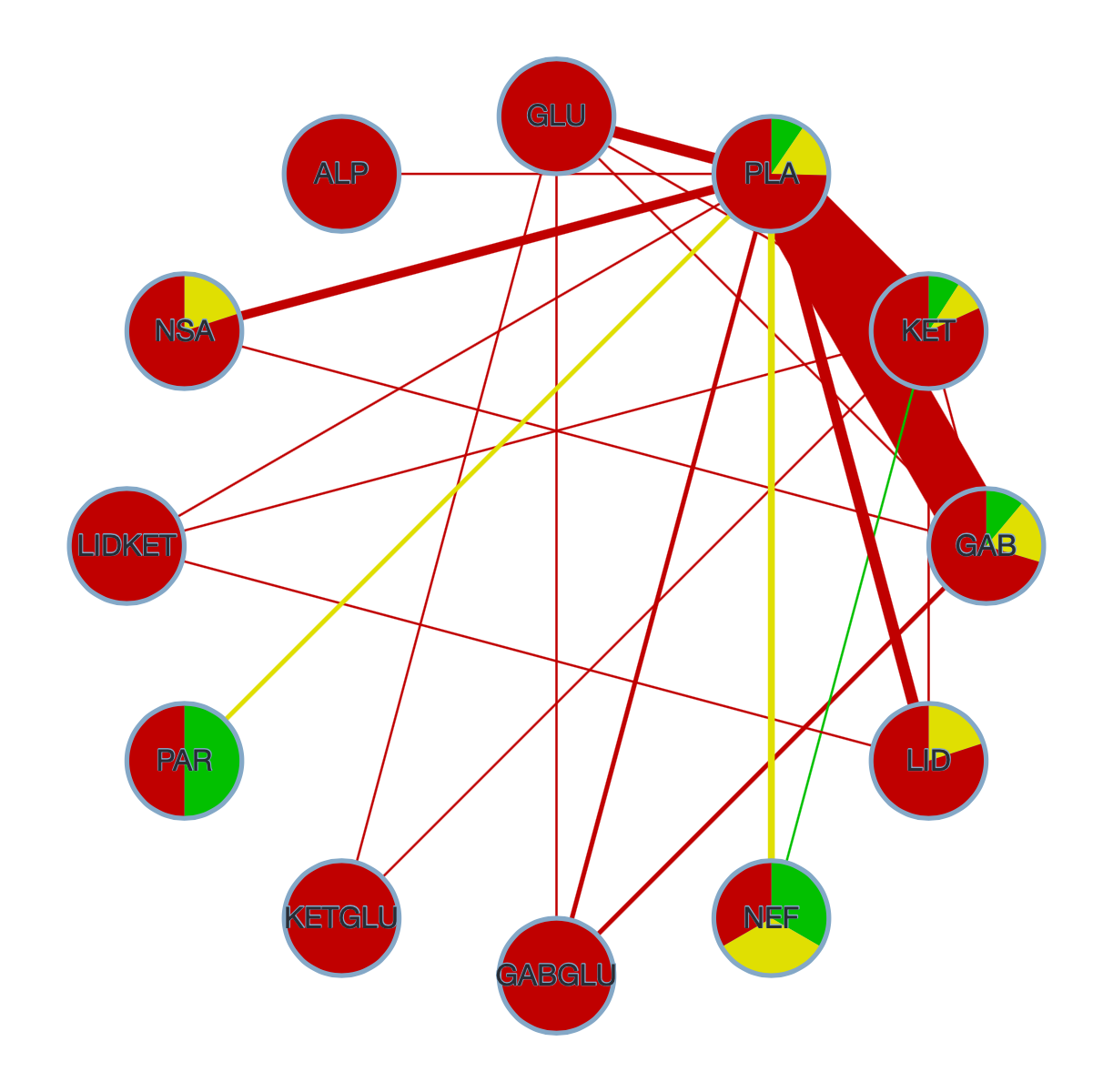
**

**Figure 41:** Network plot from CINeMA for the severity of CPSP ≤ 6 months. Nodes are equal sizes to aid visualisation. Node colour shows overall risk of bias from each intervention. The colour of edges shows the average risk of bias between comparisons with thickness being the number of studies in the comparison. Red is high risk, yellow is unclear risk and green is low risk.

**
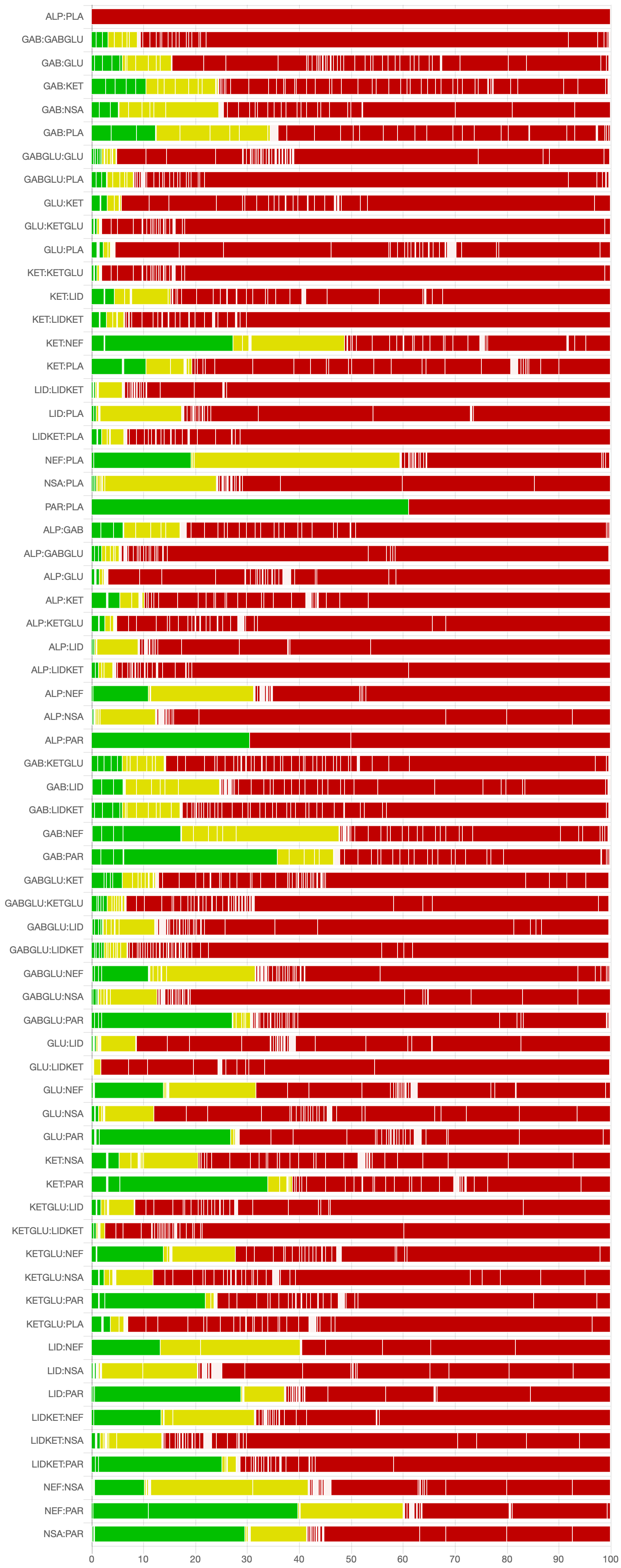
**

**Figure 42:** Contribution matrix for low, unclear and high risk of bias studies per comparison.

| **Comparison** | **N** | **Within-study bias** | **Reporting bias** | **Indirectness** | **Imprecision** | **Heterogeneity** | **Incoherence** | **Confidence** |
| --- | --- | --- | --- | --- | --- | --- | --- | --- |
| ALP-PLA | 1 | Major concerns | Low risk | No concerns | Some concerns | No concerns | Some concerns | Very low |
| GAB-GABGLU | 2 | Major concerns | Low risk | No concerns | No concerns | Some concerns | No concerns | Low |
| GAB-GLU | 1 | Major concerns | Low risk | No concerns | No concerns | Some concerns | No concerns | Low |
| GAB-KET | 1 | Major concerns | Low risk | No concerns | No concerns | Some concerns | Some concerns | Very low |
| GAB-NSA | 1 | Major concerns | Low risk | No concerns | No concerns | Some concerns | Some concerns | Very low |
| GAB-PLA | 26 | Major concerns | Low risk | No concerns | No concerns | Some concerns | Some concerns | Low* |
| GABGLU-GLU | 1 | Major concerns | Low risk | No concerns | Some concerns | No concerns | No concerns | Low |
| GABGLU-PLA | 2 | Major concerns | Low risk | No concerns | No concerns | Some concerns | No concerns | Low |
| GLU-KET | 1 | Major concerns | Low risk | No concerns | No concerns | Some concerns | Some concerns | Very low |
| GLU-KETGLU | 1 | Major concerns | Low risk | No concerns | Some concerns | No concerns | Some concerns | Very low |
| GLU-PLA | 5 | Major concerns | Low risk | No concerns | No concerns | Some concerns | Some concerns | Very low |
| KET-KETGLU | 1 | Major concerns | Low risk | No concerns | No concerns | Some concerns | Some concerns | Very low |
| KET-LID | 1 | Major concerns | Low risk | No concerns | No concerns | Some concerns | No concerns | Low |
| KET-LIDKET | 1 | Major concerns | Low risk | No concerns | Some concerns | No concerns | No concerns | Low |
| KET-NEF | 1 | Some concerns | Low risk | No concerns | No concerns | Some concerns | No concerns | Low |
| KET-PLA | 21 | Major concerns | Low risk | No concerns | No concerns | Some concerns | No concerns | Low |
| LID-LIDKET | 1 | Major concerns | Low risk | No concerns | Some concerns | No concerns | No concerns | Low |
| LID-PLA | 5 | Major concerns | Low risk | No concerns | No concerns | Some concerns | No concerns | Low |
| LIDKET-PLA | 1 | Major concerns | Low risk | No concerns | Some concerns | No concerns | No concerns | Low |
| NEF-PLA | 3 | Some concerns | Low risk | No concerns | No concerns | Some concerns | No concerns | Low |
| NSA-PLA | 4 | Major concerns | Low risk | No concerns | No concerns | Some concerns | Some concerns | Very low |
| PAR-PLA | 2 | Some concerns | Low risk | No concerns | No concerns | Some concerns | Some concerns | Very low |
| ALP-GAB | IND | Major concerns | Low risk | No concerns | Some concerns | Some concerns | Some concerns | Very low |
| ALP-GABGLU | IND | Major concerns | Low risk | No concerns | Some concerns | No concerns | Some concerns | Very low |
| ALP-GLU | IND | Major concerns | Low risk | No concerns | Some concerns | No concerns | Some concerns | Very low |
| ALP-KET | IND | Major concerns | Low risk | No concerns | Some concerns | No concerns | Some concerns | Very low |
| ALP-KETGLU | IND | Major concerns | Low risk | No concerns | Some concerns | Some concerns | Some concerns | Very low |
| ALP-LID | IND | Major concerns | Low risk | No concerns | Some concerns | Some concerns | Some concerns | Very low |
| ALP-LIDKET | IND | Major concerns | Low risk | No concerns | Some concerns | No concerns | Some concerns | Very low |
| ALP-NEF | IND | Major concerns | Low risk | No concerns | Some concerns | No concerns | Some concerns | Very low |
| ALP-NSA | IND | Major concerns | Low risk | No concerns | Some concerns | No concerns | Some concerns | Very low |
| ALP-PAR | IND | Some concerns | Low risk | No concerns | Some concerns | No concerns | Some concerns | Very low |
| GAB-KETGLU | IND | Major concerns | Low risk | No concerns | No concerns | Some concerns | Some concerns | Very low |
| GAB-LID | IND | Major concerns | Low risk | No concerns | No concerns | No concerns | Some concerns | Low |
| GAB-LIDKET | IND | Major concerns | Low risk | No concerns | Some concerns | No concerns | Some concerns | Very low |
| GAB-NEF | IND | Some concerns | Low risk | No concerns | No concerns | Some concerns | Some concerns | Very low |
| GAB-PAR | IND | Some concerns | Low risk | No concerns | No concerns | Some concerns | Some concerns | Very low |
| GABGLU-KET | IND | Major concerns | Low risk | No concerns | No concerns | Some concerns | Some concerns | Very low |
| GABGLU-KETGLU | IND | Major concerns | Low risk | No concerns | Major concerns | No concerns | Some concerns | Very low |
| GABGLU-LID | IND | Major concerns | Low risk | No concerns | Some concerns | No concerns | Some concerns | Very low |
| GABGLU-LIDKET | IND | Major concerns | Low risk | No concerns | Some concerns | No concerns | Some concerns | Very low |
| GABGLU-NEF | IND | Major concerns | Low risk | No concerns | No concerns | Some concerns | Some concerns | Very low |
| GABGLU-NSA | IND | Major concerns | Low risk | No concerns | No concerns | Some concerns | Some concerns | Very low |
| GABGLU-PAR | IND | Some concerns | Low risk | No concerns | No concerns | Some concerns | Some concerns | Very low |
| GLU-LID | IND | Major concerns | Low risk | No concerns | No concerns | Some concerns | Some concerns | Very low |
| GLU-LIDKET | IND | Major concerns | Low risk | No concerns | Major concerns | No concerns | Some concerns | Very low |
| GLU-NEF | IND | Major concerns | Low risk | No concerns | Some concerns | No concerns | Some concerns | Very low |
| GLU-NSA | IND | Major concerns | Low risk | No concerns | Some concerns | No concerns | Some concerns | Very low |
| GLU-PAR | IND | Some concerns | Low risk | No concerns | Some concerns | No concerns | Some concerns | Very low |
| KET-NSA | IND | Major concerns | Low risk | No concerns | No concerns | No concerns | Some concerns | Low |
| KET-PAR | IND | Some concerns | Low risk | No concerns | No concerns | Some concerns | Some concerns | Very low |
| KETGLU-LID | IND | Major concerns | Low risk | No concerns | Some concerns | Some concerns | Some concerns | Very low |
| KETGLU-LIDKET | IND | Major concerns | Low risk | No concerns | Some concerns | No concerns | Some concerns | Very low |
| KETGLU-NEF | IND | Major concerns | Low risk | No concerns | No concerns | Some concerns | Some concerns | Very low |
| KETGLU-NSA | IND | Major concerns | Low risk | No concerns | No concerns | Some concerns | Some concerns | Very low |
| KETGLU-PAR | IND | Major concerns | Low risk | No concerns | Some concerns | Some concerns | Some concerns | Very low |
| KETGLU-PLA | IND | Major concerns | Low risk | No concerns | No concerns | Some concerns | Some concerns | Very low |
| LID-NEF | IND | Some concerns | Low risk | No concerns | No concerns | Some concerns | Some concerns | Very low |
| LID-NSA | IND | Major concerns | Low risk | No concerns | No concerns | Some concerns | Some concerns | Very low |
| LID-PAR | IND | Some concerns | Low risk | No concerns | No concerns | Some concerns | Some concerns | Very low |
| LIDKET-NEF | IND | Major concerns | Low risk | No concerns | Some concerns | No concerns | Some concerns | Very low |
| LIDKET-NSA | IND | Major concerns | Low risk | No concerns | Some concerns | No concerns | Some concerns | Very low |
| LIDKET-PAR | IND | Some concerns | Low risk | No concerns | Some concerns | No concerns | Some concerns | Very low |
| NEF-NSA | IND | Some concerns | Low risk | No concerns | No concerns | Some concerns | Some concerns | Very low |
| NEF-PAR | IND | Some concerns | Low risk | No concerns | No concerns | Some concerns | Some concerns | Very low |
| NSA-PAR | IND | Some concerns | Low risk | No concerns | No concerns | Some concerns | Some concerns | Very low |

**Table 22:** Overall assessment of comparisons from CINeMA for the severity of CPSP ≤ 6 months. All evidence low to very low due to issues with risk of bias, inconsistency and imprecision. IND: indirect evidence. *: changed to low confidence due to sensitivity analysis of low risk of bias trials demonstrating possible benefit with gabapentinoids.

# **Severity of CPSP > 6 months (single agents)**

**Network meta-regression results**

**Figure 43:** Network plot for severity of CPSP > 6 months (single agents). Node size (black) is proportional to the number of studies evaluating that intervention and the grey lines the number of comparisons between each treatment.

| **Number of Interventions** | 6 |
| --- | --- |
| **Number of Studies** | 14 |
| **Total Number of Patients in Network** | 2273 |
| **Total Possible Pairwise Comparisons** | 15 |
| **Total Number of Pairwise Comparisons With Direct Data** | 6 |
| **Is the network connected?** | Yes |
| **Number of Two-arm Studies** | 13 |
| **Number of Multi-Arms Studies** | 1 |
| **Average outcome** | 1.36 |

| **Treatment** | **Studies** | **Participants** | **Average pain score** |
| --- | --- | --- | --- |
| **GAB** | 4 | 284 | 0.46 |
| **GLU** | 3 | 182 | 2.04 |
| **KET** | 4 | 238 | 2.06 |
| **NEF** | 1 | 22 | 0.90 |
| **NSA** | 3 | 527 | 1.23 |
| **PLA** | 14 | 1020 | 1.40 |

**Table 23:** Descriptive statistics of network and treatment characteristics for the severity of CPSP > 6 months (single agents).

**Figure 44:** Regression plot for the severity of CPSP > 6 months (single agents) demonstrating the effect of baseline risk (X-axis, mean control group pain score) compared with the effect estimate (Y-axis, mean difference). Each colour represents a different intervention. Baseline risk only associated with reductions for KET although other studies only had a few data points.

**Figure 45:** Covariate plot for the severity of CPSP > 6 months (single agents) showing the distribution of baseline risk (Y-axis) in each study (X-axis) grouped by treatment (abbreviation above plot). Red dashed line is the mean. GAB contains low baseline risk trials when compared to KET which could violate transitivity if standard network meta-analysis was performed.

**Figure 46:** SUCRA plot for ranking of treatments at a covariate value of 2 (baseline risk) for the severity of CPSP > 6 months (single agents). Treatments with lines towards the top left have a higher probability of ranking higher (KET) compared to those at the bottom right (NSA).

| **Intervention** | **SUCRA** |
| --- | --- |
| Ketamine (KET) | 82.8% |
| Nefopam (NEF) | 59.5% |
| Placebo (PLA) | 47.2% |
| Glucocorticoids (GLU) | 44.3% |
| Gabapentinoids (GAB) | 38.7% |
| NSAIDs (NSA) | 27.5% |

**Table 24:** SUCRA scores for each intervention studied for the severity of CPSP > 6 months (single agents). The higher the SUCRA %, the higher the probability that the intervention is top ranked. Note that SUCRA does not take account of risk of bias in included studies or uncertainty of estimates. Interventions are highlighted in bold green if estimates are consistent with a reduction in the severity of pain (no interventions).

**Figure 47:** League heat plot for the severity of CPSP > 6 months (single agents) demonstrating effects of treatment (X-axis) compared with comparator (Y-axis) at a fixed covariate value of 2. Green cells are those consistent with a reduction in pain (darker colour means stronger effect) whilst red interventions are consistent with an increase in pain (darker colour means stronger effect). Those highlighted with a ****** have credible intervals consistent with an effect (no interventions).

**Figure 48:** Forest plot showing the posterior median mean difference with 95% CrIs at a fixed covariate value of 2 for the severity of CPSP > 6 months (single agents). No estimates consistent with a reduction in the severity of pain. X-axis is on a linear scale. Effects are relative to placebo.

**Diagnostics**

Residual deviance of 28.5 compared with 29 data points.

- For initial model settings, some PSRF values approached unacceptable levels so MCMC iterations increased in steps to get convergence, which ended on 20,000 adaptations, 40,000 burn-in and 200,000 for inference iterations. Following this, improvement in trace plots and all PSRF values **<1.009** (trace and density plots below)

**
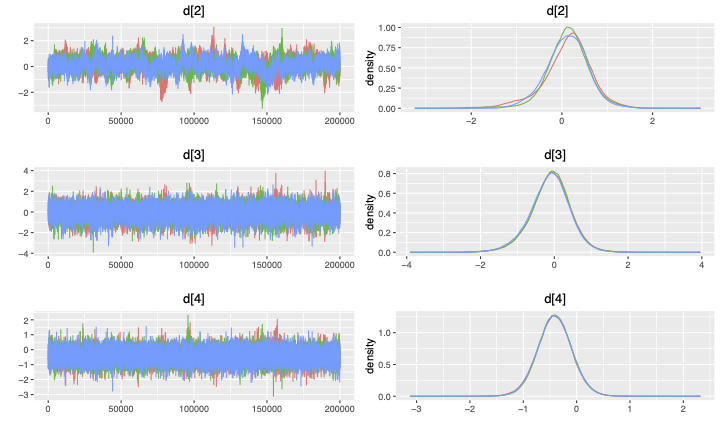
**

**
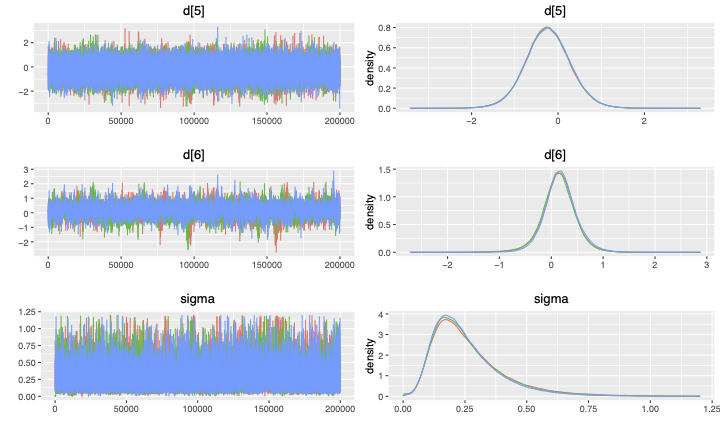
**

**Inconsistency assessment (no baseline risk adjustment)**

Unable to perform for this analysis.

**Sensitivity analysis**

**Low risk of bias only for severity of CPSP > 6 months**

**Figure 49:** Forest plot showing the posterior median mean difference with 95% CrIs at a fixed covariate value of 2 in low risk of bias trials only for the severity of CPSP > 6 months (single agents). No estimates consistent with a reduction in the severity of pain at > 6 months. X-axis is on a linear scale. Effects are relative to placebo.

# **CINeMA assessment for severity of CPSP > 6 months**

**
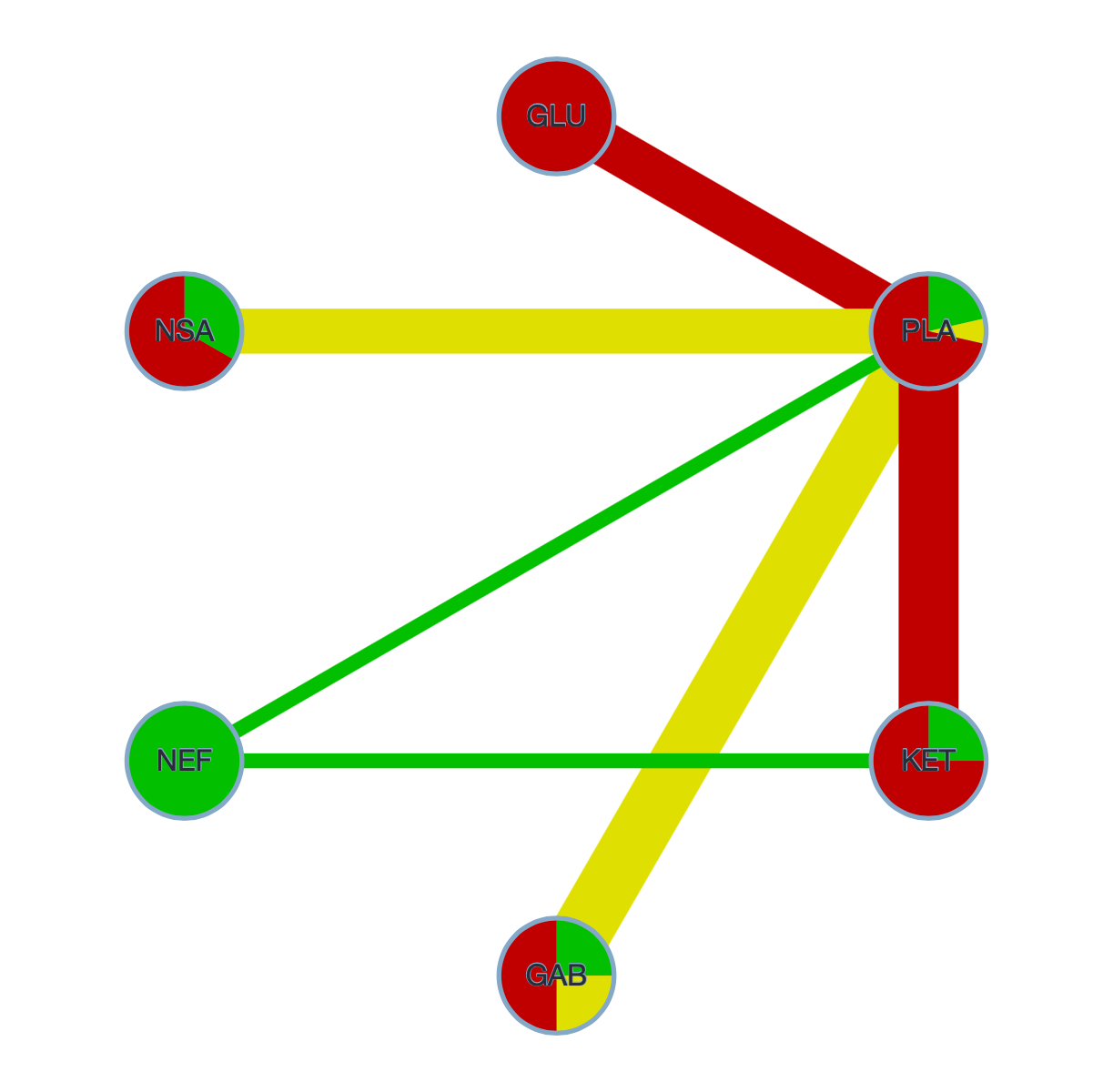
**

**Figure 50:** Network plot from CINeMA for the severity of CPSP > 6 months. Nodes are equal sizes to aid visualisation. Node colour shows overall risk of bias for each intervention. The colour of edges shows the average risk of bias between comparisons with thickness being the number of studies in the comparison. Red is high risk, yellow is unclear risk and green is low risk.

**
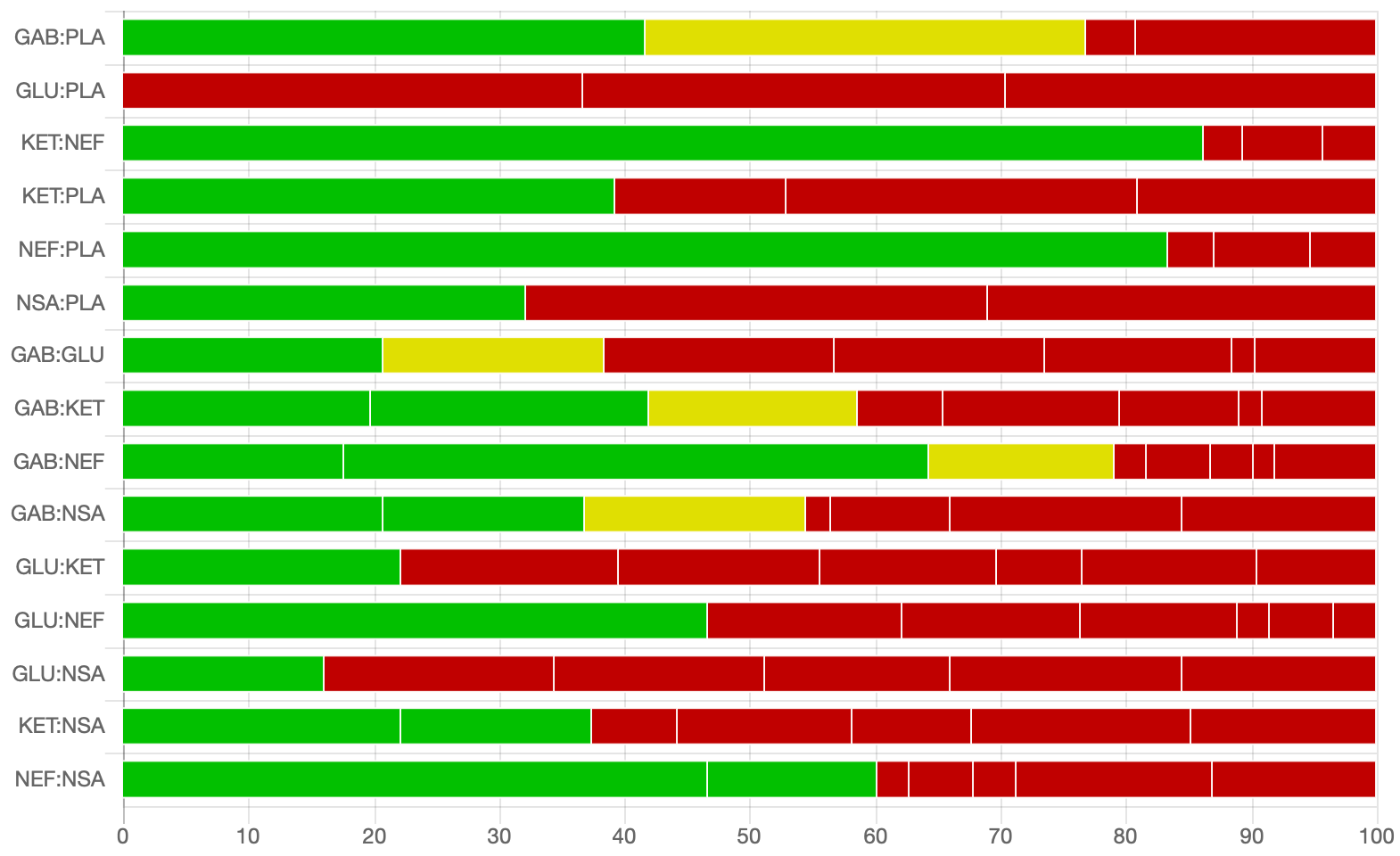
**

**Figure 51:** Contribution matrix for low, unclear and high risk of bias studies per comparison.

| **Comparison** | **N** | **Within-study bias** | **Reporting bias** | **Indirectness** | **Imprecision** | **Heterogeneity** | **Incoherence** | **Confidence** |
| --- | --- | --- | --- | --- | --- | --- | --- | --- |
| GAB-PLA | 4 | Some concerns | Low risk | No concerns | No concerns | No concerns | No concerns | Moderate |
| GLU-PLA | 3 | Major concerns | Low risk | No concerns | No concerns | No concerns | No concerns | Moderate |
| KET-NEF | 1 | No concerns | Low risk | No concerns | No concerns | Some concerns | No concerns | Moderate |
| KET-PLA | 4 | Some concerns | Low risk | No concerns | No concerns | Some concerns | No concerns | Low |
| NEF-PLA | 1 | No concerns | Low risk | No concerns | Some concerns | No concerns | No concerns | Moderate |
| NSA-PLA | 3 | Some concerns | Low risk | No concerns | No concerns | No concerns | No concerns | Moderate |
| GAB-GLU | IND | Some concerns | Low risk | No concerns | No concerns | No concerns | No concerns | Moderate |
| GAB-KET | IND | Some concerns | Low risk | No concerns | Some concerns | No concerns | No concerns | Low |
| GAB-NEF | IND | Some concerns | Low risk | No concerns | Some concerns | No concerns | No concerns | Low |
| GAB-NSA | IND | Some concerns | Low risk | No concerns | No concerns | No concerns | No concerns | Moderate |
| GLU-KET | IND | Major concerns | Low risk | No concerns | Some concerns | No concerns | No concerns | Low |
| GLU-NEF | IND | Some concerns | Low risk | No concerns | Some concerns | No concerns | No concerns | Low |
| GLU-NSA | IND | Major concerns | Low risk | No concerns | No concerns | Some concerns | No concerns | Low |
| KET-NSA | IND | Some concerns | Low risk | No concerns | No concerns | Some concerns | No concerns | Low |
| NEF-NSA | IND | Some concerns | Low risk | No concerns | Some concerns | No concerns | No concerns | Low |

**Table 25:** Overall assessment of comparisons from CINeMA for the severity of CPSP > 6 months. All evidence moderate to low mainly due to issues with risk of bias. IND: indirect evidence.

# **Serious adverse events (single agents)**

**Network meta-analysis**

This analysis was performed for single agents only due to problems running the models including the low number of events for combination agents.

**Figure 52:** Network plot for serious adverse events. Node size (black) is proportional to the number of studies evaluating that intervention and the grey lines the number of comparisons between each treatment.

| **Number of Interventions** | 7 |
| --- | --- |
| **Number of Studies** | 38 |
| **Total Number of Patients in Network** | 12367 |
| **Total Possible Pairwise Comparisons** | 21 |
| **Total Number of Pairwise Comparisons With Direct Data** | 7 |
| **Is the network connected?** | Yes |
| **Number of Two-arm Studies** | 37 |
| **Number of Multi-Arms Studies** | 1 |
| **Total Number of Events in Network** | 2112 |
| **Number of Studies With No Zero Events** | 22 |
| **Number of Studies With At Least One Zero Event** | 16 |
| **Number of Studies with All Zero Events** | 0 |

| **Treatment** | **Studies** | **Events** | **Participants** |
| --- | --- | --- | --- |
| **ALP** | 1 | 2 | 31 |
| **GAB** | 14 | 30 | 900 |
| **GLU** | 4 | 925 | 3965 |
| **KET** | 10 | 43 | 591 |
| **LID** | 5 | 4 | 211 |
| **NSA** | 5 | 74 | 619 |
| **PLA** | 38 | 1034 | 6050 |

**Table 26:** Descriptive statistics of network and treatment characteristics for serious adverse events.

**Figure 53:** SUCRA plot for serious adverse events. Treatments with lines towards the top left have a higher probability of ranking higher (less SAEs) compared to those at the bottom right (ALP).

| **Intervention** | **SUCRA** |
| --- | --- |
| Gabapentinoids (GAB) | 61.7% |
| Glucocorticoids (GLU) | 61.5% |
| Ketamine (KET) | 59.6% |
| Placebo (PLA) | 52.8% |
| Lidocaine (LID) | 51.1% |
| NSAIDs (NSA) | 35.5% |
| Alpha-2 agonists (ALP) | 27.9% |

**Table 27:** SUCRA scores for each intervention for serious adverse events. The higher the SUCRA %, the higher the probability that the intervention is top ranked (lower SAE events). Note that SUCRA does not take account of risk of bias in included studies or uncertainty of estimates. Interventions are highlighted in bold green if estimates are consistent with a reduction in SAEs (no interventions).

**Figure 54:** League heat plot demonstrating effects of treatment (X-axis) compared with comparator (Y-axis) for serious adverse events. Green cells are those consistent with a reduction in SAEs (darker colour means stronger effect) whilst red interventions are consistent with an increase in SAEs (darker colour means stronger effect). Those highlighted with a ****** have credible intervals consistent with an effect (no interventions).

**Figure 55:** Forest plot showing the posterior median odds ratio with 95% CrIs for serious adverse events. No estimates consistent with a difference in SAEs although many estimates were imprecise. X-axis is on a log scale to aid visualisation due to extreme values. Effects are relative to placebo.

**Diagnostics**

Residual deviance of 90.6 compared with 77 data points.

- The above suggests some problems with model fit
- All PSRF values **<1.002** (trace and density plots)

**
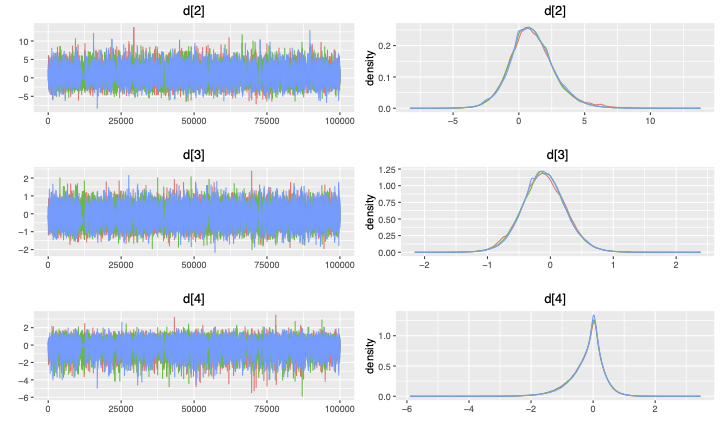
**

**
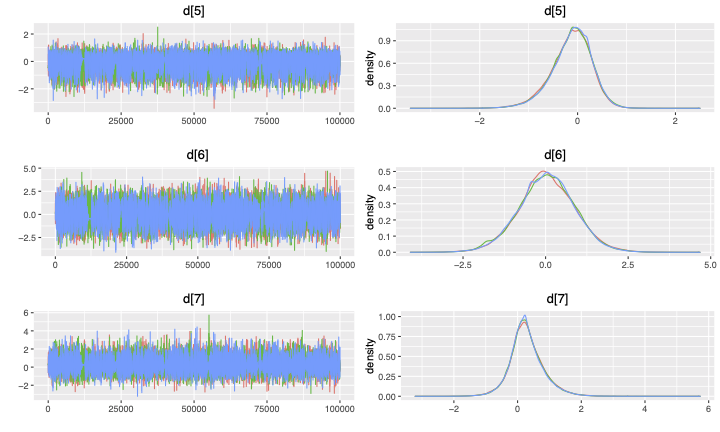
**

**
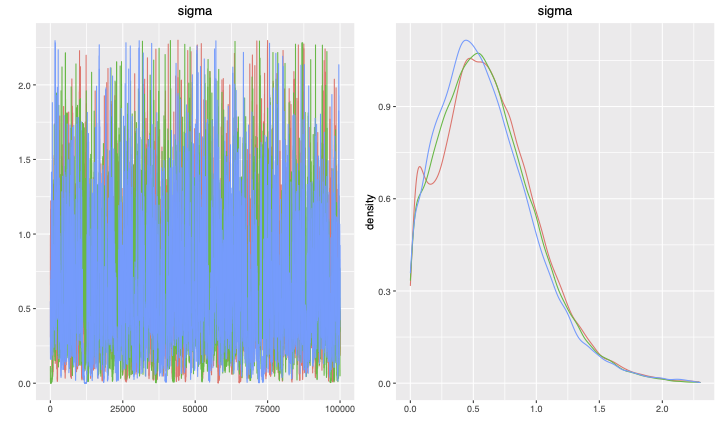
**

**Inconsistency assessment (no baseline risk adjustment)**

| **Comparison** | **P value** |
| --- | --- |
| GAB-LID | 0.55 |

**Table 28:** Node-splitting method to assess inconsistency for serious adverse events. No evidence of inconsistency although a limited number of nodes and imprecision in direct estimates.

**Sensitivity analysis**

**Low risk of bias only for SAEs**

**Figure 56:** Forest plot showing the posterior median odds ratio with 95% CrIs in low risk of bias trials only (6 trials) for serious adverse events. No estimates consistent with a difference is SAEs. X-axis is on a log scale to aid visualisation due to extreme values. Effects are relative to placebo.

# **CINeMA assessment for serious adverse events**

**
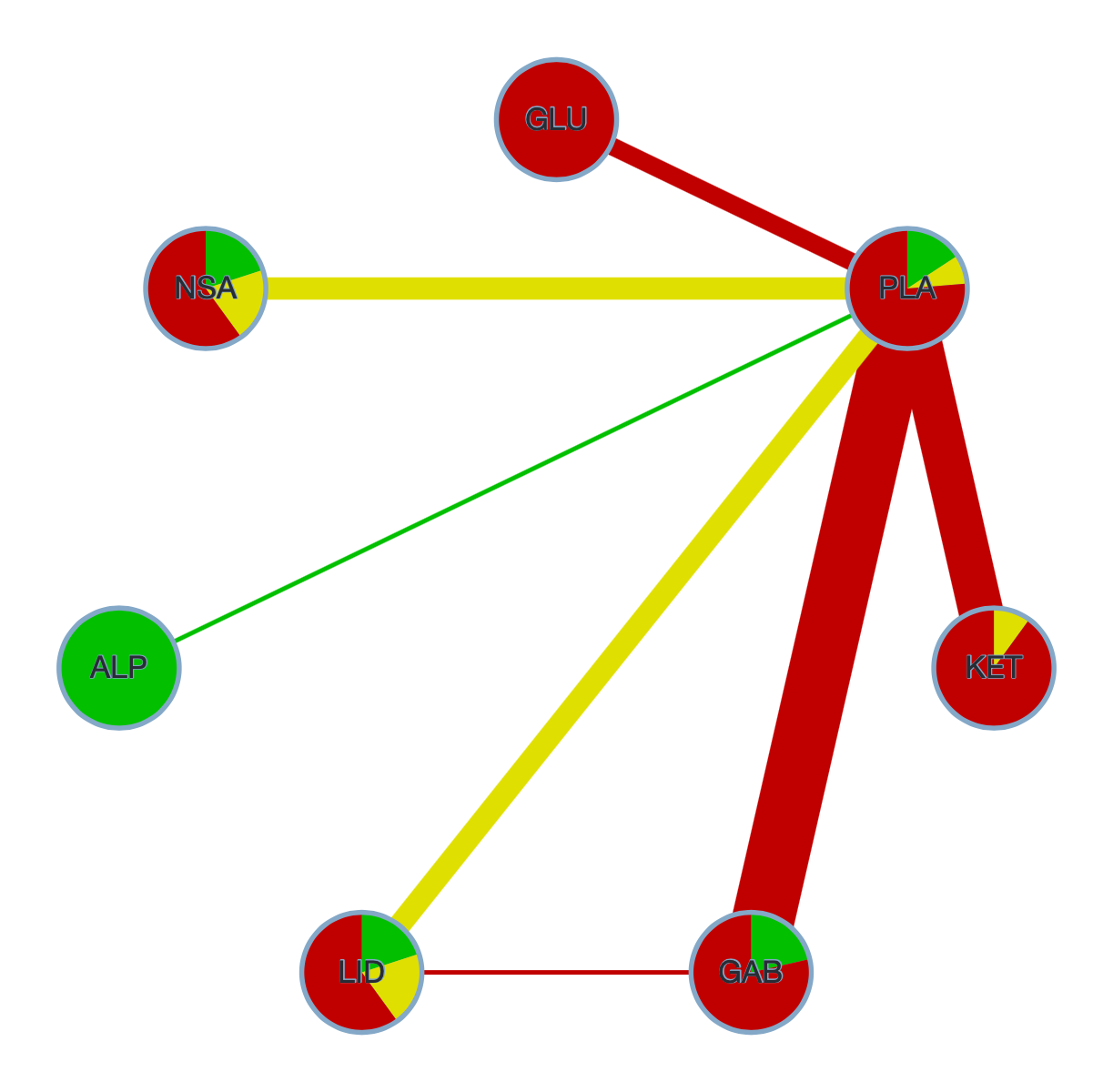
**

**Figure 57:** Network plot from CINeMA for serious adverse events. Nodes are equal sizes to aid visualisation. Node colour shows overall risk of bias for each intervention. The colour of edges shows the average risk of bias between comparisons with thickness being the number of studies in the comparison. Red is high risk, yellow is unclear risk and green is low risk.

**
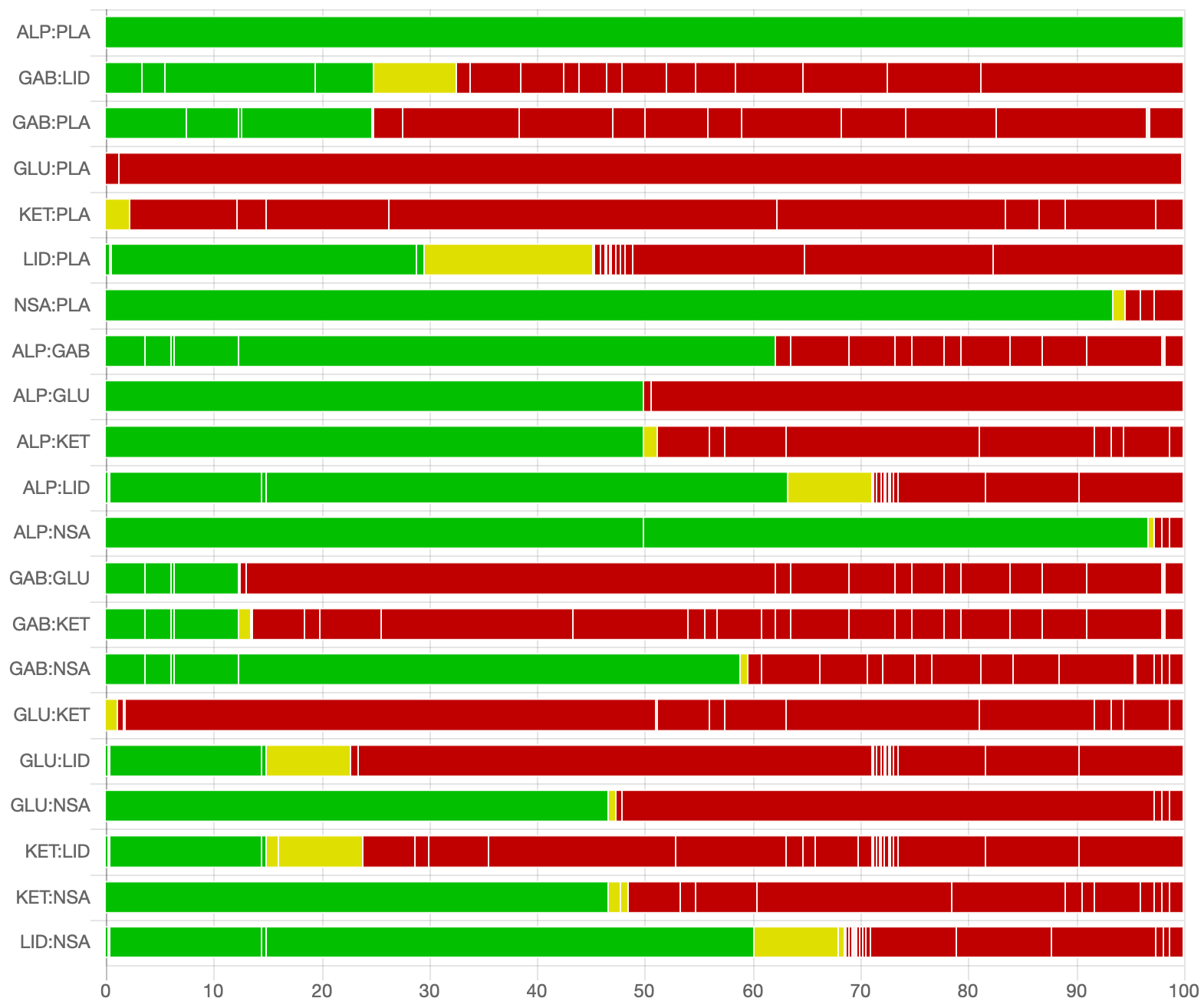
**

**Figure 58:** Contribution matrix for low, unclear and high risk of bias studies per comparison.

| **Comparison** | **N** | **Within-study bias** | **Reporting bias** | **Indirectness** | **Imprecision** | **Heterogeneity** | **Incoherence** | **Confidence** |
| --- | --- | --- | --- | --- | --- | --- | --- | --- |
| ALP-PLA | 1 | No concerns | Low risk | No concerns | Major concerns | No concerns | No concerns | Moderate |
| GAB-LID | 1 | Some concerns | Low risk | No concerns | Major concerns | No concerns | No concerns | Low |
| GAB-PLA | 14 | Major concerns | Low risk | No concerns | Major concerns | No concerns | No concerns | Low |
| GLU-PLA | 4 | Major concerns | Low risk | No concerns | Major concerns | No concerns | No concerns | Low |
| KET-PLA | 10 | Major concerns | Low risk | No concerns | Major concerns | No concerns | No concerns | Low |
| LID-PLA | 5 | Some concerns | Low risk | No concerns | Major concerns | No concerns | No concerns | Low |
| NSA-PLA | 5 | No concerns | Low risk | No concerns | Major concerns | No concerns | No concerns | Moderate |
| ALP-GAB | IND | Some concerns | Low risk | No concerns | Major concerns | No concerns | No concerns | Low |
| ALP-GLU | IND | Some concerns | Low risk | No concerns | Major concerns | No concerns | No concerns | Low |
| ALP-KET | IND | Some concerns | Low risk | No concerns | Major concerns | No concerns | No concerns | Low |
| ALP-LID | IND | Some concerns | Low risk | No concerns | Major concerns | No concerns | No concerns | Low |
| ALP-NSA | IND | No concerns | Low risk | No concerns | Major concerns | No concerns | No concerns | Moderate |
| GAB-GLU | IND | Major concerns | Low risk | No concerns | Major concerns | No concerns | No concerns | Low |
| GAB-KET | IND | Major concerns | Low risk | No concerns | Major concerns | No concerns | No concerns | Low |
| GAB-NSA | IND | Some concerns | Low risk | No concerns | Major concerns | No concerns | No concerns | Low |
| GLU-KET | IND | Major concerns | Low risk | No concerns | Major concerns | No concerns | No concerns | Low |
| GLU-LID | IND | Major concerns | Low risk | No concerns | Major concerns | No concerns | No concerns | Low |
| GLU-NSA | IND | Some concerns | Low risk | No concerns | Major concerns | No concerns | No concerns | Low |
| KET-LID | IND | Major concerns | Low risk | No concerns | Major concerns | No concerns | No concerns | Low |
| KET-NSA | IND | Some concerns | Low risk | No concerns | Major concerns | No concerns | No concerns | Low |
| LID-NSA | IND | Some concerns | Low risk | No concerns | Major concerns | No concerns | No concerns | Low |

**Table 29:** Overall assessment of comparisons from CINeMA for serious adverse events. All evidence moderate to low mainly due to issues with risk of bias. IND: indirect evidence.

# **Incidence of opioid use ≤ 6 months (combination agents)**

**Network meta-analysis results**

One study including lidocaine had to be removed due to problems with MCMC convergence (OR 0.08; 95% confidence interval 0.01 to 1.47). Analysis performed for combination agents only as they contributed only one event.

**Figure 59:** Network plot for the incidence of opioid use ≤ 6 months (combination agents). Node size (black) is proportional to the number of studies evaluating that intervention and the grey lines the number of comparisons between each treatment.

| **Number of Interventions** | 5 |
| --- | --- |
| **Number of Studies** | 9 |
| **Total Number of Patients in Network** | 1284 |
| **Total Possible Pairwise Comparisons** | 10 |
| **Total Number of Pairwise Comparisons With Direct Data** | 7 |
| **Is the network connected?** | Yes |
| **Number of Two-arm Studies** | 8 |
| **Number of Multi-Arms Studies** | 1 |
| **Total Number of Events in Network** | 135 |
| **Number of Studies With No Zero Events** | 6 |
| **Number of Studies With At Least One Zero Event** | 3 |
| **Number of Studies with All Zero Events** | 0 |

| **Treatment** | **Studies** | **Events** | **Participants** |
| --- | --- | --- | --- |
| **GAB** | 6 | 39 | 459 |
| **GABGLU** | 1 | 1 | 38 |
| **GLU** | 1 | 3 | 36 |
| **KET** | 3 | 22 | 147 |
| **PLA** | 9 | 70 | 604 |

**Table 30:** Descriptive statistics of network and treatment characteristics for the incidence of opioid use ≤ 6 months (combination agents).

**Figure 60:** SUCRA plot for incidence of opioid use ≤ 6 months (combination agents). Treatments with lines towards the top left have a higher probability of ranking higher (KET) compared to those at the bottom right (GLU).

| **Intervention** | **SUCRA** |
| --- | --- |
| Ketamine (KET) | 85.1% |
| Gabapentinoids and Glucocorticoids (GABGLU) | 58.3% |
| Placebo (PLA) | 53.8% |
| Gabapentinoids (GAB) | 41.8% |
| Glucocorticoids (GLU) | 11% |

**Table 31:** SUCRA scores for each intervention studied for the incidence of opioid use ≤ 6 months (combination agents). The higher the SUCRA %, the higher the probability that the intervention is top ranked. Note that SUCRA does not take account of risk of bias in included studies or uncertainty of estimates. Interventions are highlighted in bold green if estimates are consistent with a reduction in the incidence of opioid use (no interventions).

**Figure 61:** League heat plot for the incidence of opioid use ≤ 6 months (combination agents) demonstrating effects of treatment (X-axis) compared with comparator (Y-axis). Green cells are those consistent with a reduction in opioid use (darker colour means stronger effect) whilst red interventions are consistent with an increase in opioid use (darker colour means stronger effect). Those highlighted with a ****** have credible intervals consistent with an effect (no interventions).

**Figure 62:** Forest plot showing the posterior median odds ratio with 95% CrIs for incidence of opioid use ≤ 6 months. No estimates consistent with a difference. X-axis on log scale to improve visualisation due to extreme values. Effects are presented relative to placebo.

**Inconsistency assessment (no baseline risk adjustment)**

Unable to perform for this outcome.

**Sensitivity analysis**

**Low risk of bias only for incidence of opioid use ≤ 6 months**

Only 3 studies remained comparing gabapentinoids to placebo (OR 0.74; 95% CrI 0.26 to 2.45).

**Diagnostics**

Residual deviance of 20.8 compared with 20 data points.

- All PSRF values **<1.002** (trace and density plots below)


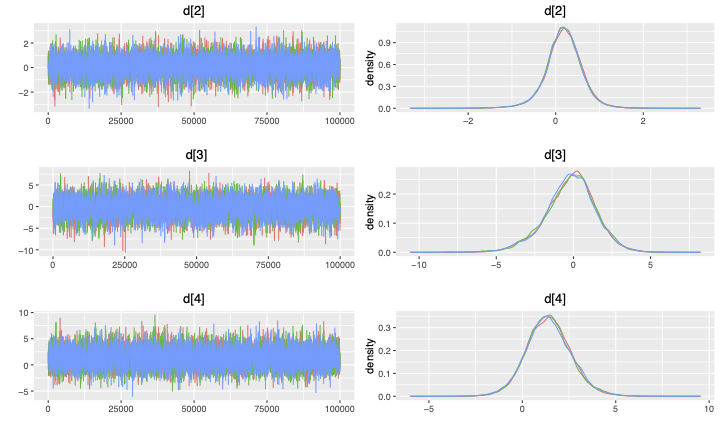


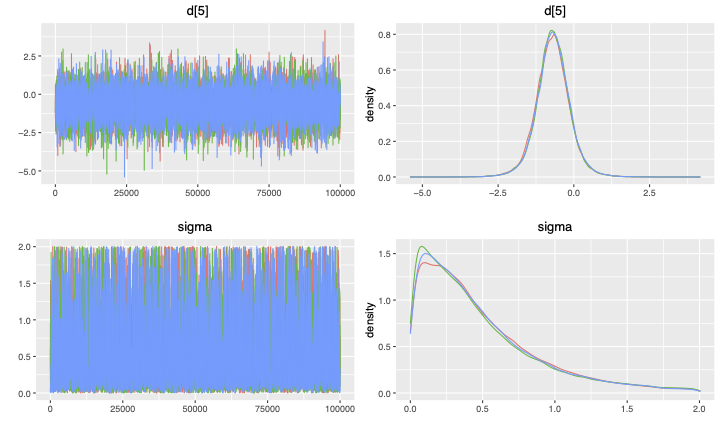


# **CINeMA assessment for incidence of opioids ≤ 6 months**

**
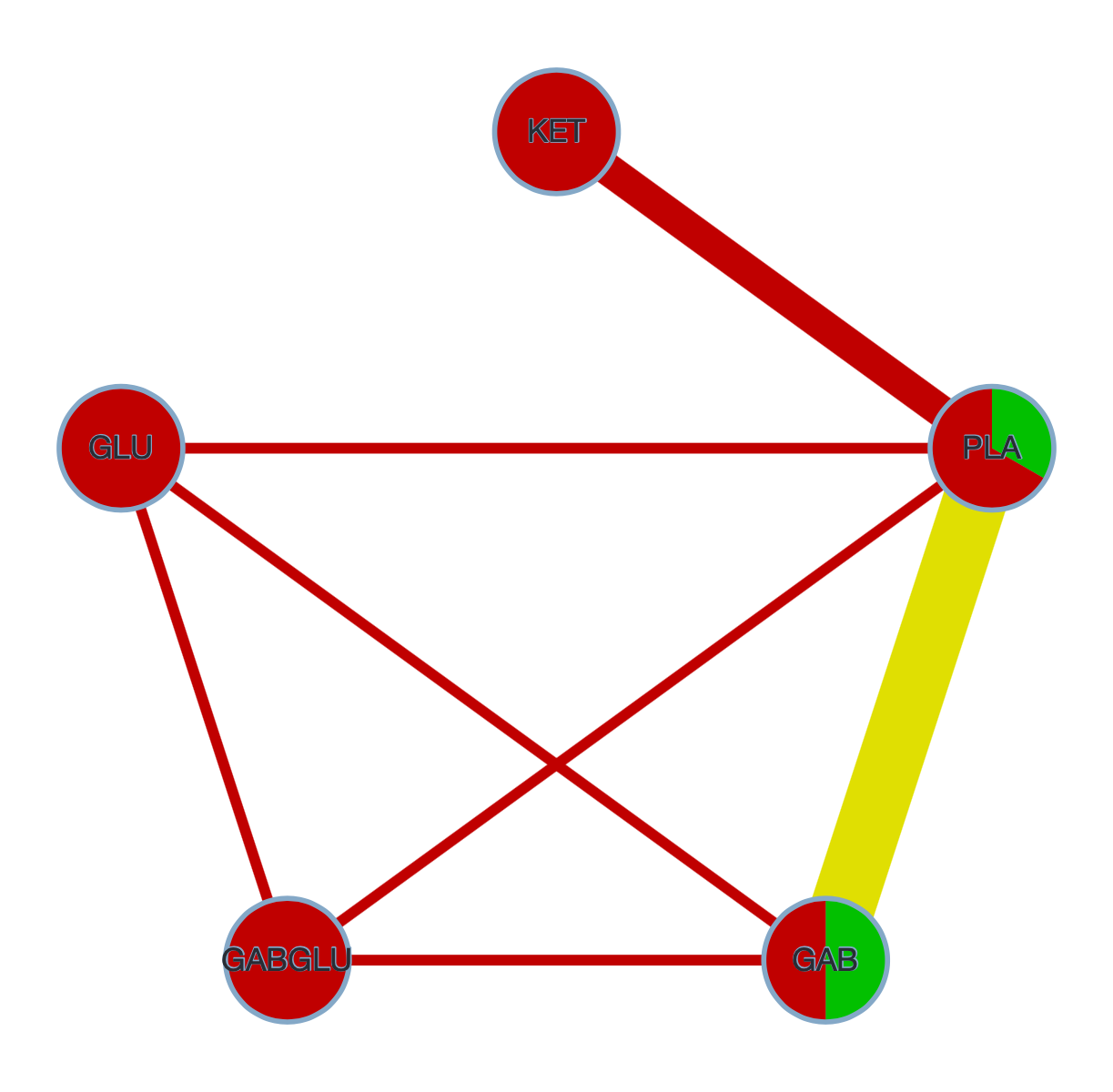
**

**Figure 63:** Network plot from CINeMA for the incidence of opioid use ≤ 6 months. Nodes are equal sizes to aid visualisation. Node colour shows overall risk of bias for each intervention. The colour of the edges shows the average risk of bias between comparisons with thickness being the number of studies in the comparison. Red is high risk, yellow is unclear risk and green is low risk.

**
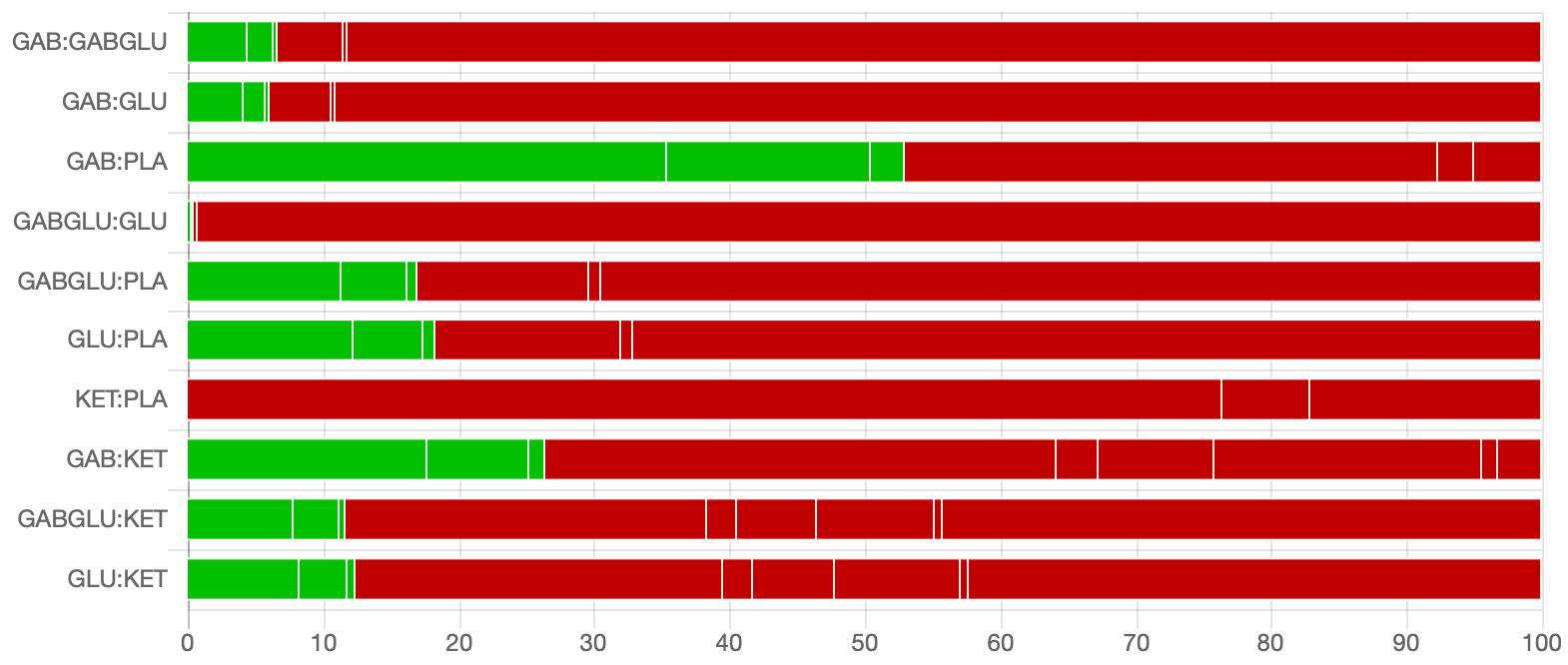
**

**Figure 64:** Contribution matrix for low, unclear and high risk of bias studies per comparison.

| **Comparison** | **N** | **Within-study bias** | **Reporting bias** | **Indirectness** | **Imprecision** | **Heterogeneity** | **Incoherence** | **Confidence** |
| --- | --- | --- | --- | --- | --- | --- | --- | --- |
| GAB-GABGLU | 1 | Major concerns | Low risk | No concerns | Major concerns | No concerns | No concerns | Low |
| GAB-GLU | 1 | Major concerns | Low risk | No concerns | Major concerns | No concerns | No concerns | Low |
| GAB-PLA | 6 | Some concerns | Low risk | No concerns | Major concerns | No concerns | No concerns | Low |
| GABGLU-GLU | 1 | Major concerns | Low risk | No concerns | Major concerns | No concerns | No concerns | Low |
| GABGLU-PLA | 1 | Major concerns | Low risk | No concerns | Major concerns | No concerns | No concerns | Low |
| GLU-PLA | 1 | Major concerns | Low risk | No concerns | Major concerns | No concerns | No concerns | Low |
| KET-PLA | 3 | Major concerns | Low risk | No concerns | Some concerns | No concerns | No concerns | Low |
| GAB-KET | IND | Some concerns | Low risk | No concerns | Some concerns | Some concerns | No concerns | Very low |
| GABGLU-KET | IND | Major concerns | Low risk | No concerns | Major concerns | No concerns | No concerns | Low |
| GLU-KET | IND | Major concerns | Low risk | No concerns | Major concerns | No concerns | No concerns | Low |

**Table 32:** Overall assessment of comparisons from CINeMA for the incidence of opioid use ≤ 6 months. All evidence low to very low mainly due to issues with risk of bias. IND: indirect evidence.

# **Incidence of opioid use > 6 months (single agents)**

**Network meta-analysis results**

**Figure 65:** Network plot for the incidence of opioid use > 6 months (single agents). Node size (black) is proportional to the number of studies evaluating that intervention and the grey lines the number of comparisons between each treatment.

| **Number of Interventions** | 3 |
| --- | --- |
| **Number of Studies** | 3 |
| **Total Number of Patients in Network** | 604 |
| **Total Possible Pairwise Comparisons** | 3 |
| **Total Number of Pairwise Comparisons With Direct Data** | 3 |
| **Is the network connected?** | Yes |
| **Number of Two-arm Studies** | 3 |
| **Number of Multi-Arms Studies** | 0 |
| **Total Number of Events in Network** | 14 |
| **Number of Studies With No Zero Events** | 2 |
| **Number of Studies With At Least One Zero Event** | 1 |
| **Number of Studies with All Zero Events** | 0 |

| **Treatment** | **Studies** | **Events** | **Participants** |
| --- | --- | --- | --- |
| **GAB** | 1 | 4 | 208 |
| **KET** | 2 | 2 | 101 |
| **PLA** | 3 | 8 | 295 |

**Table 33:** Descriptive statistics of network and treatment characteristics for the incidence of opioid use > 6 months (single agents).

**Figure 66:** SUCRA plot for the incidence of opioid use > 6 months. Treatments with lines towards the top left have a higher probability of ranking higher (KET) compared to those at the bottom right (GAB).

| **Intervention** | **SUCRA** |
| --- | --- |
| Ketamine (KET) | 88% |
| Placebo (PLA) | 35.9% |
| Gabapentinoids (GAB) | 26.2% |

**Table 34:** SUCRA scores for each intervention studied. The higher the SUCRA %, the higher the probability that the intervention is top ranked. Note that SUCRA does not take account of risk of bias in included studies or uncertainty of estimates. Interventions are highlighted in bold green if estimates are consistent with a reduction in the incidence of opioid use (no interventions).

**Figure 67:** League heat plot for the incidence of opioid use > 6 months (single agents) demonstrating effects of treatment (X-axis) compared with comparator (Y-axis). Green cells are those consistent with a reduction in opioid use (darker colour means stronger effect) whilst red interventions are consistent with an increase in opioid use (darker colour means stronger effect). Those highlighted with a ****** have credible intervals consistent with an effect (no interventions).

**Figure 68:** Forest plot showing the posterior median odds ratio with 95% CrIs for incidence of opioid use > 6 months. No estimates consistent with a difference. X-axis on log scale to aid visualisation due to extreme values. Effects are relative to placebo.

**Inconsistency assessment (no baseline risk adjustment)**

Unable to perform for this outcome.

**Sensitivity analysis**

**Low risk of bias only for incidence of opioid use > 6 months**

Only one study remained comparing gabapentinoids to placebo (OR 1.3; 95% confidence interval 0.29 to 5.89).

**Diagnostics**

Residual deviance of 6 compared with 6 data points.

- All PSRF values **<1.001** (trace and density plots below)


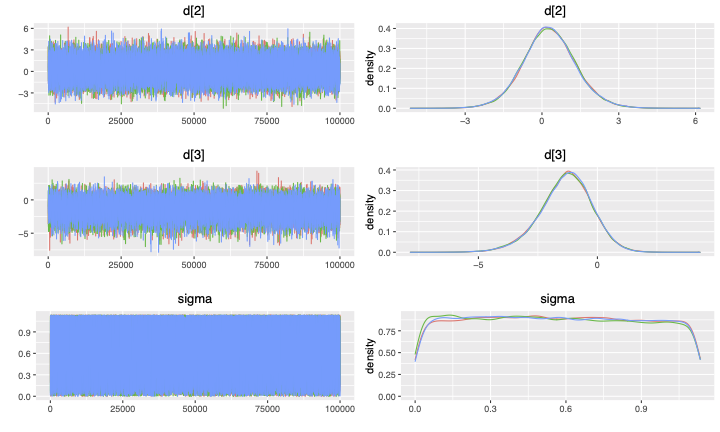


# **CINeMA assessment for incidence of opioids > 6 months**

**
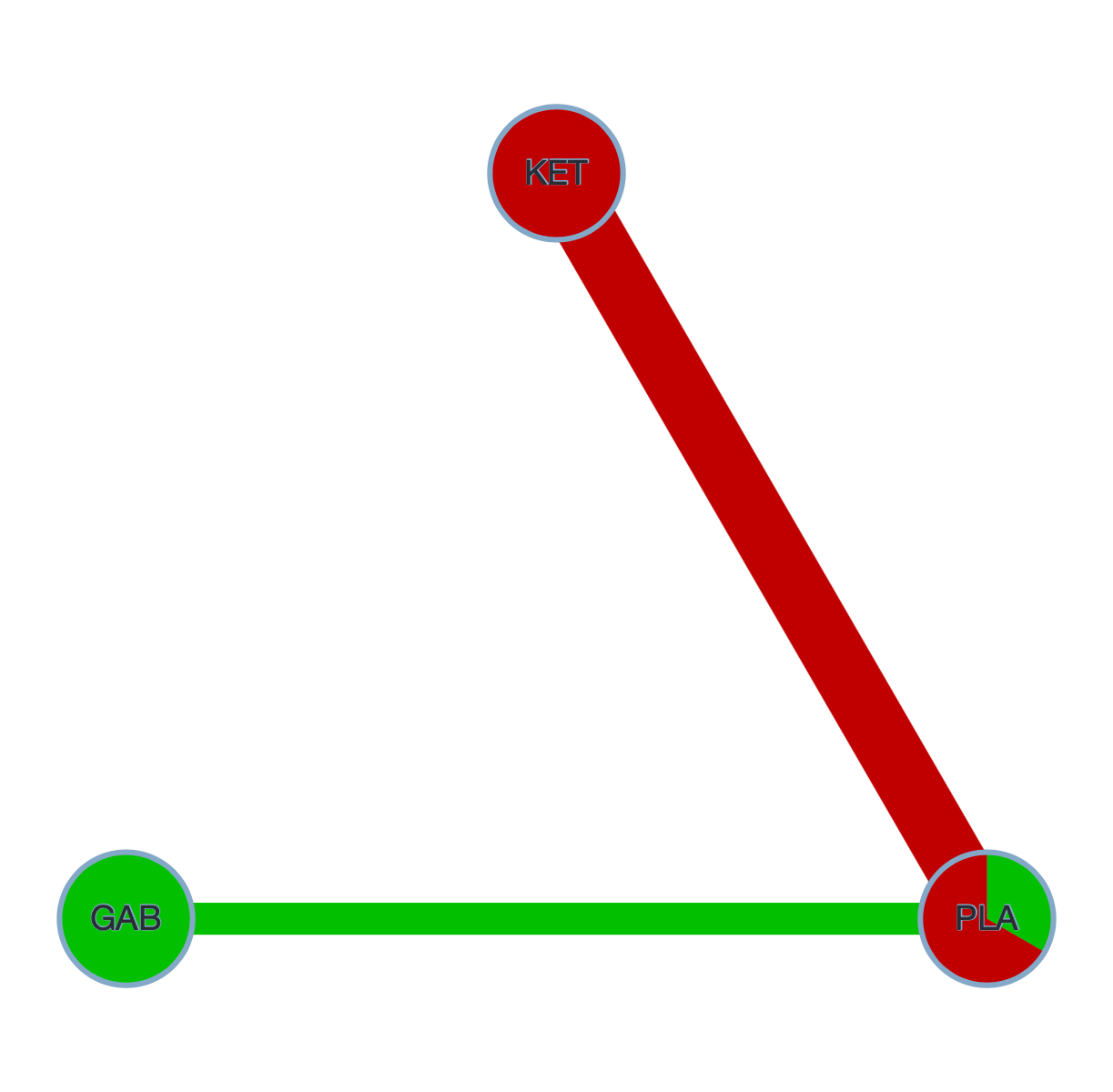
**

**Figure 69:** Network plot from CINeMA for the incidence of opioid use > 6 months. Node are equal sizes to aid visualisation. Node colour shows overall risk of bias from each intervention. The colour of edges shows the average risk of bias between comparisons with thickness being the number of studies in the comparison. Red is high risk, yellow is unclear risk and green is low risk.

**
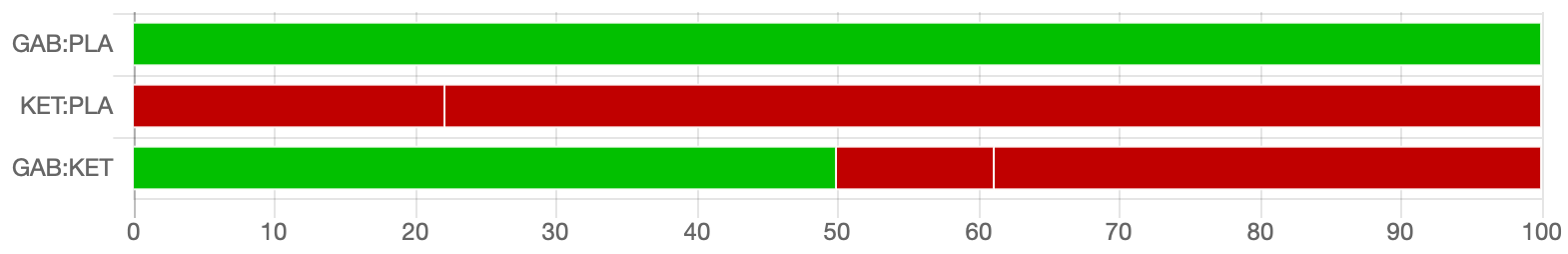
**

**Figure 70:** Contribution matrix for low, unclear and high risk of bias studies per comparison.

| **Comparison** | **N** | **Within-study bias** | **Reporting bias** | **Indirectness** | **Imprecision** | **Heterogeneity** | **Incoherence** | **Confidence** |
| --- | --- | --- | --- | --- | --- | --- | --- | --- |
| GAB-PLA | 1 | No concerns | Low risk | No concerns | Major concerns | No concerns | Major concerns | Low |
| KET-PLA | 2 | Major concerns | Low risk | No concerns | Major concerns | No concerns | Major concerns | Very low |
| GAB-KET | IND | Some concerns | Low risk | No concerns | Major concerns | No concerns | Major concerns | Very low |

**Table 35:** Overall assessment of comparisons from CINeMA for the incidence of opioid use > 6 months. All evidence low to very low mainly due to issues with risk of bias, imprecision and incoherence. IND: indirect evidence.

# **Severity of opioid use ≤ 6 months (single agents)**

**Network meta-analysis results**

**Figure 71:** Network plot for the severity of opioid use ≤ 6 months (single agents). Node size (black) is proportional to the number of studies evaluating that intervention and the grey lines the number of comparisons between each treatment.

| **Number of Interventions** | 3 |
| --- | --- |
| **Number of Studies** | 3 |
| **Total Number of Patients in Network** | 199 |
| **Total Possible Pairwise Comparisons** | 3 |
| **Total Number of Pairwise Comparisons With Direct Data** | 2 |
| **Is the network connected?** | Yes |
| **Number of Two-arm Studies** | 3 |
| **Number of Multi-Arms Studies** | 0 |
| **Average outcome** | 8.74 |

| **Treatment** | **Studies** | **Participants** |
| --- | --- | --- |
| **GAB** | 1 | 16 |
| **KET** | 2 | 77 |
| **PLA** | 3 | 106 |

**Table 36:** Descriptive statistics of network and treatment characteristics for the severity of opioid use ≤ 6 months (single agents).

**Figure 72:** SUCRA plot for the severity of opioid use ≤ 6 months (single agents). Treatments with lines towards the top left have a higher probability of ranking higher (KET) compared to those at the bottom right (PLA).

| **Intervention** | **SUCRA** |
| --- | --- |
| Ketamine (KET) | 73.4% |
| Gabapentinoids (GAB) | 42.1% |
| Placebo (PLA) | 34.5% |

**Table 37:** SUCRA scores for each intervention studied for the severity of opioid use ≤ 6 months (single agents). The higher the SUCRA %, the higher the probability that the intervention is top ranked. Note that SUCRA does not take account of risk of bias in included studies or uncertainty of estimates. Interventions are highlighted in bold green if estimates are consistent with a reduction in the severity of opioid use (no interventions).

**Figure 73:** League heat plot for the severity of opioid use ≤ 6 months (single agents) demonstrating effects of treatment (X-axis) compared with comparator (Y-axis). Green cells are those consistent with a reduction in opioid use (darker colour means stronger effect) whilst red interventions are consistent with an increase in opioid use (darker colour means stronger effect). Those highlighted with a ****** have credible intervals consistent with an effect (no interventions).

**Figure 74:** Forest plot showing the posterior median mean difference with 95% CrIs for severity of opioid use ≤ 6 months (single agents). No estimates consistent with a difference. X-axis on linear scale. Effects are relative to placebo.

**Inconsistency assessment (no baseline risk adjustment)**

Unable to perform for this outcome.

**Sensitivity analysis**

**Low risk of bias only for severity of opioid use ≤ 6 months**

No studies were low risk of bias.

**Diagnostics**

Residual deviance of 6.4 compared with 6 data points.

- All PSRF values **<1.001** (trace and density plots below)

**
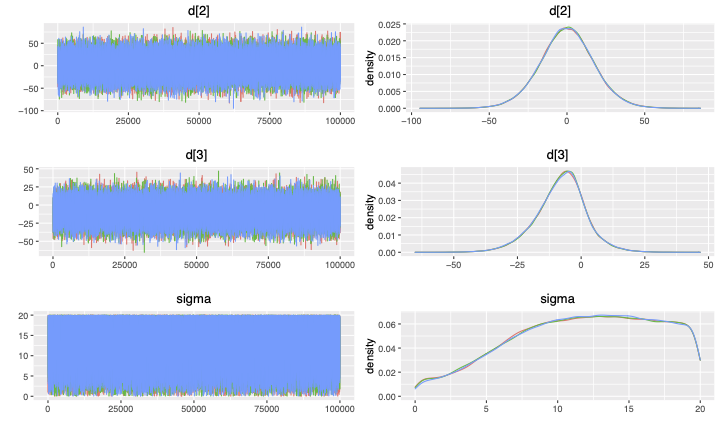
**

# **CINeMA assessment for severity of opioid use ≤ 6 months**

**
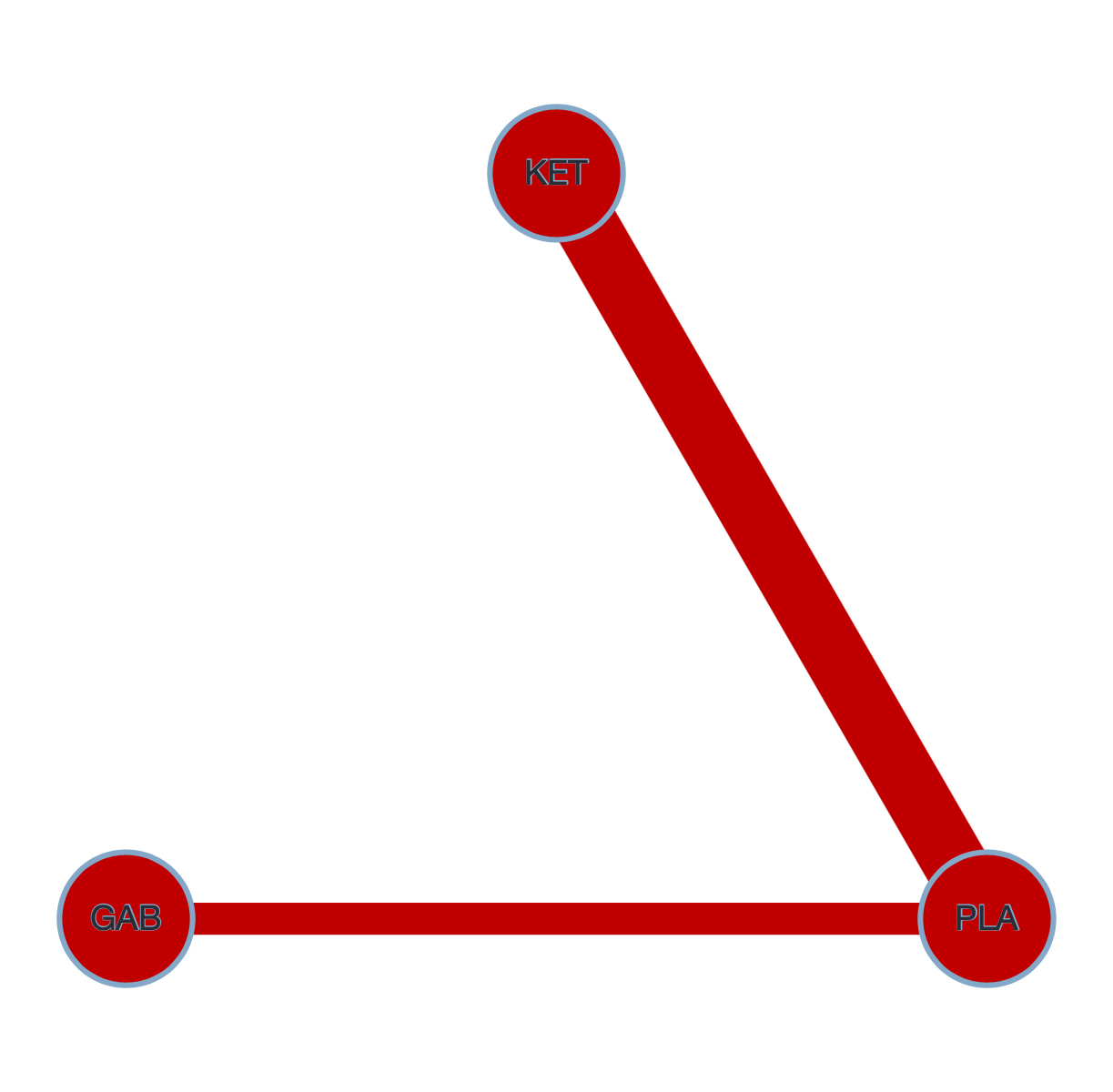
**

**Figure 75:** Network plot from CINeMA for the severity of opioid use ≤ 6 months. Nodes are equal sizes to aid visualisation. Node colour shows overall risk of bias from each intervention. The colour of edges shows the average risk of bias between comparisons with thickness being the number of studies in the comparison. Red is high risk, yellow is unclear risk and green is low risk.

**
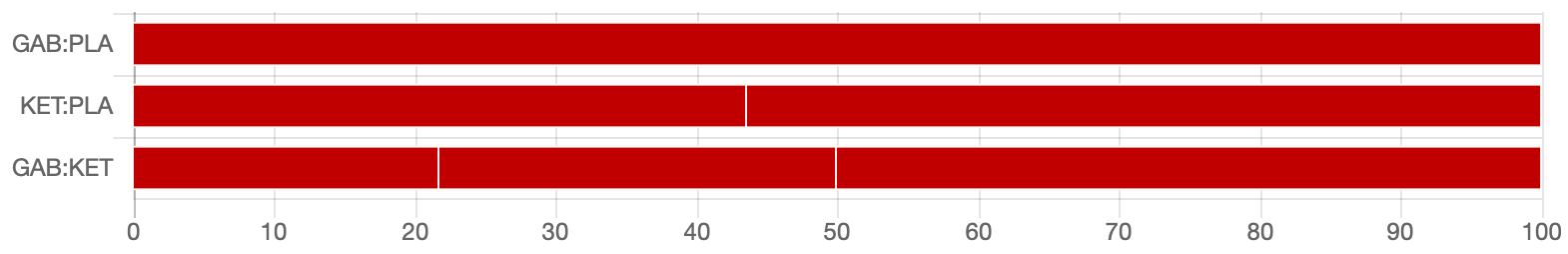
**

**Figure 76:** Contribution matrix for low, unclear and high risk of bias studies per comparison.

| **Comparison** | **N** | **Within-study bias** | **Reporting bias** | **Indirectness** | **Imprecision** | **Heterogeneity** | **Incoherence** | **Confidence** |
| --- | --- | --- | --- | --- | --- | --- | --- | --- |
| GAB-PLA | 1 | Major concerns | Low risk | No concerns | Major concerns | No concerns | No concerns | Low |
| KET-PLA | 2 | Major concerns | Low risk | No concerns | Major concerns | No concerns | No concerns | Low |
| GAB-KET | IND | Major concerns | Low risk | No concerns | Major concerns | No concerns | No concerns | Low |

**Table 38:** Overall assessment of comparisons from CINeMA for the severity of opioid use ≤ 6 months. All evidence low confidence due to issues with risk of bias and imprecision. IND: indirect evidence.

# **Severity of opioid use > 6 months (single agents)**

Only one study which evaluated ketamine versus placebo demonstrated a possible reduction with ketamine (MD -20mg; 95% confidence interval -33.9mg to -6.1mg).

# **R code used in analysis**

X = name of dataset

**library(BUGSnet)**

**#NMA of binary outcome**

**#Data preparation**

dataprep <- data.prep(arm.data = X,

varname.t = "treatment",

varname.s = "study")

**#Network plot**

net.plot(dataprep, node.scale = 0.5,

edge.scale=0.5,

node.colour = "black",

edge.colour = "grey",

label.offset1 = 5)

**#Network characteristics**

network.char <- net.tab(data = dataprep,

outcome = "events",

N = "sampleSize",

type.outcome = "binomial")

network.char$network

**#Characteristics by treatment**

network.char$intervention

**#Characteristics by comparison**

network.char$comparison

**#Random-effects model**

random_effects_model <- nma.model(data=dataprep,

outcome="events",

N="sampleSize",

reference="PLA",

family="binomial",

link="logit",

effects="random")

random_effects_results <- nma.run(random_effects_model,

n.adapt=10000,

n.burnin=20000,

n.iter=100000)

**#Assess model fit**

nma.fit(random_effects_results, main= "Random Effects Model")

**#SUCRA plot**

sucra.out <- nma.rank(random_effects_results, largerbetter=FALSE, sucra.palette= "Set1")

sucra.out$sucraplot

sucra.out$sucratable

**#League heat plot**

league.out <- nma.league(random_effects_results,

central.tdcy = "median",

order = as.vector(t(dataprep$treatments)),

digits = 2,

log.scale = FALSE,

low.colour = "darkgreen",

mid.colour = "white",

high.colour = "red")

league.out$heatplot

**#Forest plot**

nma.forest(random_effects_results,

central.tdcy="median",

comparator = "PLA")

**#Check inconsistency (not used in this review)**

re_inconsistency_model <- nma.model(data=dataprep,

outcome="events",

N="sampleSize",

reference="PLA",

family="binomial",

link="log",

type = "inconsistency",

effects="random")

re_inconsistency_results <- nma.run(re_inconsistency_model,

n.adapt=10000,

n.burnin=20000,

n.iter=100000)

**#Inconsistency plot run**

par(mfrow = c(1,2))

re_model_fit <- nma.fit(random_effects_results)

inconsist_model_fit <- nma.fit(re_inconsistency_results)

**#Inconsistency plot**

nma.compare(re_model_fit, inconsist_model_fit)

**#Diagnostics**

nma.diag(random_effects_results)

**#Network meta-regression (binary outcome)**

**#Data preparation**

dataprep <- data.prep(arm.data = X,

varname.t = "treatment",

varname.s = "study")

**#Network plot (colours removed)**

net.plot(dataprep, node.scale = 0.5,

edge.scale=0.5,

label.offset1 = 5)

**#Network characteristics**

network.char <- net.tab(data = dataprep,

outcome = "events",

N = "sampleSize",

type.outcome = "binomial")

network.char$network

**#Intervention characteristics**

network.char$intervention

**#Comparison characteristics**

network.char$comparison

**#Random-effects model meta-regression with baseline risk covariate (exchangeable)**

random_effects_model <- nma.model(data=dataprep,

outcome="events",

N="sampleSize",

reference="PLA",

family="binomial",

link="logit",

effects="random",

covariate="baselinerisk",

prior.beta="EXCHANGEABLE")

**#Random-effects results**

random_effects_results <- nma.run(random_effects_model,

n.adapt=10000,

n.burnin=20000,

n.iter=100000)

**#Assess model fit**

nma.fit(random_effects_results, main= "Random Effects Model")

**#Regression plot**

nma.regplot(random_effects_results)

**#Distribution of baseline risk by study (covariate plot)**

data.plot(data = dataprep,

covariate = "baselinerisk",

avg.hline = TRUE,

text.size = 10,

by = "treatment")

**#SUCRA plot and tables at covariate value of 0.35**

sucra.out <- nma.rank(random_effects_results,

largerbetter=FALSE,

cov.value=0.35,

sucra.palette= "Set1")

sucra.out$sucraplot

sucra.out$ranktable

sucra.out$sucratable

sucra.out$order

**#League heat plot at covariate level of 0.35**

league.out <- nma.league(random_effects_results,

central.tdcy = "median",

order = as.vector(t(dataprep$treatments)),

cov.value=0.35,

digits = 2,

low.colour = "darkgreen",

mid.colour = "white",

high.colour = "red",

log.scale = FALSE)

league.out$heatplot

**#Forest plot of relative effects at covariate level of 0.35 (log transformed X-axis)**

nma.forest(random_effects_results,

comparator="PLA",

central.tdcy = "median",

cov.value=0.35,

x.trans="log")

**#Diagnostics**

nma.diag(random_effects_results)

**#Check inconsistency (not used in this review)**

re_inconsistency_model <- nma.model(data=dataprep,

outcome="events",

N="sampleSize",

reference="PLA",

family="binomial",

link="log",

type = "inconsistency",

effects="random")

re_inconsistency_results <- nma.run(re_inconsistency_model,

n.adapt=10000,

n.burnin=20000,

n.iter=100000)

**#Inconsistency plot run**

par(mfrow = c(1,2))

re_model_fit <- nma.fit(random_effects_results)

inconsist_model_fit <- nma.fit(re_inconsistency_results)

**#Inconsistency plot**

nma.compare(re_model_fit, inconsist_model_fit)

**#Network meta-regression (continuous outcome)**

**#Data preparation**

dataprep <- data.prep(arm.data = X,

varname.t = "treatment",

varname.s = "study")

**#Network plot**

net.plot(dataprep, node.scale = 0.5,

edge.scale=0.5,

node.colour = "black",

edge.colour = "grey",

label.offset1 = 5)

**#Network characteristics**

network.char <- net.tab(data = dataprep,

outcome="mean",

N = "sampleSize",

type.outcome = "continuous")

network.char$network

**#Intervention characteristics**

network.char$intervention

**#Comparison characteristics**

network.char$comparison

**#Random-effects model meta-regression with baseline risk covariate**

random_effects_model <- nma.model(data=dataprep,

outcome="mean",

sd="SD",

N="sampleSize",

reference="PLA",

family="normal",

link="identity",

effects="random",

covariate="baselinerisk",

prior.beta="EXCHANGEABLE")

random_effects_results <- nma.run(random_effects_model,

n.adapt=10000,

n.burnin=20000,

n.iter=100000)

**#Assess model fit**

nma.fit(random_effects_results, main= "Random Effects Model")

**#Regression plot**

nma.regplot(random_effects_results)

**#Distribution of baseline risk by study** **(covariate plot)**

data.plot(data = dataprep,

covariate = "baselinerisk",

avg.hline = TRUE,

text.size = 10,

by = "treatment")

**#SUCRA plot and tables at covariate value of 2**

sucra.out <- nma.rank(random_effects_results,

largerbetter=FALSE,

cov.value=2,

sucra.palette= "Set1")

sucra.out$sucraplot

sucra.out$ranktable

sucra.out$sucratable

sucra.out$order

**#League heat plot at covariate level of 2**

league.out <- nma.league(random_effects_results,

central.tdcy = "median",

order = as.vector(t(dataprep$treatments)),

cov.value=2,

digits = 2,

log.scale = FALSE,

low.colour = "darkgreen",

mid.colour = "white",

high.colour = "red")

league.out$heatplot

**#Forest plot of relative effects at covariate level of 2**

nma.forest(random_effects_results,

comparator="PLA",

central.tdcy = "median",

cov.value=2)

**#Diagnostics**

nma.diag(random_effects_results)

**#Check inconsistency (not used in this review)**

re_inconsistency_model <- nma.model(data=dataprep,

outcome="mean",

sd="SD",

N="sampleSize",

reference="PLA",

family="normal",

type="inconsistency",

link="identity",

effects="random",

covariate="baselinerisk",

prior.beta="EXCHANGEABLE")

re_inconsistency_results <- nma.run(re_inconsistency_model,

n.adapt=10000,

n.burnin=20000,

n.iter=100000)

**#Inconsistency plot run**

par(mfrow = c(1,2))

re_model_fit <- nma.fit(random_effects_results)

inconsist_model_fit <- nma.fit(re_inconsistency_results)

**#Inconsistency plot**

nma.compare(re_model_fit, inconsist_model_fit)

**#gemtc** (needs column titles to be study, treatment, responders and sampleSize or mean, std.dev and sampleSize)

**library(gemtc)**

**#Set up data (raw data)**

network <- mtc.network(data.ab = X)

**#Network information**

summary(network)

**#Network plot**

plot(network)

**#Compile consistency random-effects model with 4 Markov chains**

model <- mtc.model(network,

linearModel = "random",

type = "consistency",

dic = TRUE,

n.chain = 4)

**#Run MCMC simulation**

mcmc <- mtc.run(model, n.adapt = 10000, n.iter = 100000, thin = 10)

**#Trace and density plot**

plot(mcmc)

**#Gelman plot**

gelman.plot(mcmc)

**#PSRF**

gelman.diag(mcmc)$mpsrf

**#Node-split assessment of inconsistency**

nodesplit <- mtc.nodesplit(network,

linearModel = "random",

n.adapt = 10000,

n.iter = 100000,

thin = 10)

summary(nodesplit)

plot(summary(nodesplit))

**#Network meta-regression**

**#Setup** (requires two data frames, one main data set and another which has one line per study and the covariate on)

network.mr <- mtc.network(data.ab = X,

studies = covariate.dataframe)

**#Regression (shared coefficients as per transitivity assessment)**

regressor <- list(coefficient = 'shared',

variable = 'baselinerisk',

control = 'PLA')

**#Setup model**

model.mr <- mtc.model(network.mr,

type = "regression",

regressor = regressor)

**#Run model**

mcmc <- mtc.run(model.mr,

n.adapt = 10000,

n.iter = 100000,

thin = 10)

**#Model results** (median for beta coefficient is under "2. Quantiles for each variable" and the row 'B' with median at 50% and 95% CrIs are 2.5% and 97.5%)

summary(mcmc)

# **WinBUGS code for baseline risk analysis**

Adapted from: Dias S, Ades AE, Welton NJ, Jansen JP and Sutton AS (2018) Network Meta-Analysis for Decision Making. John Wiley & Sons Ltd

**#Network meta-regression for binomial outcome taking uncertainty of covariate into account**

**# Binomial likelihood, logit link**

**# Random effects model for multi-arm trials**

model{ **# *** PROGRAM STARTS**

for(i in 1:ns){ **# LOOP THROUGH STUDIES**

w[i,1] <- 0 **# adjustment for multi-arm trials is zero for control arm**

delta[i,1] <- 0 **# treatment effect is zero for control arm**

mu[i] ~ dnorm(0,.001**) # vague priors for all trial baselines**

for (k in 1:na[i]) { **# LOOP THROUGH ARMS**

r[i,k] ~ dbin(p[i,k],n[i,k]) **# binomial likelihood**

**# model for linear predictor**

logit(p[i,k]) <- mu[i] + delta[i,k]

+ (beta[t[i,k]]-beta[t[i,1]]) * (mu[i]-mx)

rhat[i,k] <- p[i,k] * n[i,k] **# expected value of the numerators**

**#Deviance contribution**

dev[i,k] <- 2 * (r[i,k] * (log(r[i,k])-log(rhat[i,k]))

+ (n[i,k]-r[i,k]) * (log(n[i,k]-r[i,k]) - log(n[i,k]-rhat[i,k]))) }

**# summed residual deviance contribution for this trial**

resdev[i] <- sum(dev[i,1:na[i]])

for (k in 2:na[i]) { **# LOOP THROUGH ARMS**

**# trial-specific LOR distributions**

delta[i,k] ~ dnorm(md[i,k],taud[i,k])

**# mean of LOR distributions (with multi-arm trial correction)**

**# covariate effect relative to treat in arm 1**

md[i,k] <- d[t[i,k]] - d[t[i,1]] + sw[i,k]

**# precision of LOR distributions (with multi-arm trial correction)**

taud[i,k] <- tau *2*(k-1)/k

**# adjustment for multi-arm RCTs**

w[i,k] <- (delta[i,k] - d[t[i,k]] + d[t[i,1]])

**# cumulative adjustment for multi-arm trials**

sw[i,k] <- sum(w[i,1:k-1])/(k-1)

}

}

totresdev <- sum(resdev[]) **# Total Residual Deviance**

d[1]<-0 **# treatment effect is zero for reference treatment**

beta[1] <- 0 **# covariate effect is zero for reference treatment**

**# vague priors for treatment effects**

for (k in 2:nt){

d[k] ~ dnorm(0,.0001)

beta[k] <- B **# common covariate effect**

}

B ~ dnorm(0,.0001) **# vague prior for covariate effect**

sd ~ dunif(0,5) **# vague prior for between-trial SD**

tau <- pow(sd,-2) **# between-trial precision = (1/between-trial variance)**

**# treatment effect when covariate = z[j] (un-centring treatment effects)**

for (k in 1:nt){

for (j in 1:nz) { dz[j,k] <- d[k] - (beta[k]-beta[1])*(mx-z[j]) }

}

**# pairwise ORs and LORs for all possible pair-wise comparisons, if nt>2**

for (c in 1:(nt-1)) {

for (k in (c+1):nt) {

**# at mean value of covariate**

or[c,k] <- exp(d[k] - d[c])

lor[c,k] <- (d[k]-d[c])

**# at covariate=z[j]**

for (j in 1:nz) {

orz[j,c,k] <- exp(dz[j,k] - dz[j,c])

lorz[j,c,k] <- (dz[j,k]-dz[j,c])

}

}

}

} **# *** PROGRAM ENDS**

**#Network meta-regression for continuous outcome taking uncertainty of covariate into account**

**# Normal likelihood, identity link**

**# Random effects model for multi-arm trials**

**model**{

**# *** PROGRAM STARTS**

for(i in 1:ns){ **# LOOP THROUGH STUDIES**

w[i,1] <- 0 **# adjustment for multi-arm trials is zero for control arm**

delta[i,1] <- 0 **# treatment effect is zero for control arm**

mu[i] ~ dnorm(0,.001) **# vague priors for all trial baselines**

for (k in 1:na[i]) { **# LOOP THROUGH ARMS**

var[i,k] <- pow(se.y[i,k],2)

prec[i,k] <- 1/var[i,k] **# set precisions**

y[i,k] ~ dnorm(theta[i,k],prec[i,k]) **# Normal likelihood**

**# model for linear predictor**

theta[i,k] <- mu[i] + delta[i,k]

+ (beta[t[i,k]]-beta[t[i,1]]) * (mu[i]-mx)

**#Deviance contribution**

dev[i,k] <- (y[i,k]-theta[i,k])*(y[i,k]-theta[i,k])*prec[i,k]

}

**# summed residual deviance contribution for this trial**

resdev[i] <- sum(dev[i,1:na[i]])

for (k in 2:na[i]) { **# LOOP THROUGH ARMS**

**# trial-specific LOR distributions**

delta[i,k] ~ dnorm(md[i,k],taud[i,k])

**# mean of LOR distributions (with multi-arm trial correction)**

**# covariate effect relative to treat in arm 1**

md[i,k] <- d[t[i,k]] - d[t[i,1]] + sw[i,k]

**# precision of LOR distributions (with multi-arm trial correction)**

taud[i,k] <- tau *2*(k-1)/k

**# adjustment for multi-arm RCTs**

w[i,k] <- (delta[i,k] - d[t[i,k]] + d[t[i,1]])

**# cumulative adjustment for multi-arm trials**

sw[i,k] <- sum(w[i,1:k-1])/(k-1)

}

}

totresdev <- sum(resdev[]) **# Total Residual Deviance**

d[1]<-0 **# treatment effect is zero for reference treatment**

beta[1] <- 0 **# covariate effect is zero for reference treatment**

**# vague priors for treatment effects**

for (k in 2:nt){

d[k] ~ dnorm(0,.0001)

beta[k] <- B **# common covariate effect**

}

B ~ dnorm(0,.0001) **# vague prior for covariate effect**

sd ~ dunif(0,5) **# vague prior for between-trial SD**

tau <- pow(sd,-2) **# between-trial precision = (1/between-trial variance)**

**# mean differences**

**for (c in 1:(nt-1))** {

for (k in (c+1):nt) {

md[c,k] <- (d[k]-d[c])

}

}

} **# *** PROGRAM ENDS**

# **PRISMA-NMA Checklist**

1. Identify the report as a systematic review incorporating a network meta-analysis (or related form of meta-analysis) -> **page 1**
2. Provide a structured summary including, as applicable -> **page 2** (limited by word count)
3. Describe the rationale for the review in the context of what is already known, including mention of why a network meta-analysis has been conducted -> **page 3**
4. Provide an explicit statement of questions being addressed, with reference to participants, interventions, comparisons, outcomes, and study design (PICOS) -> **page 4-5**
5. Indicate whether a review protocol exists and if and where it can be accessed (e.g., Web address); and, if available, provide registration information, including registration number -> **page 4**
6. Specify study characteristics (e.g., PICOS, length of follow-up) and report characteristics (e.g., years considered, language, publication status) used as criteria for eligibility, giving rationale. Clearly describe eligible treatments included in the treatment network, and note whether any have been clustered or merged into the same node (with justification) -> **page 4-5**
7. Describe all information sources (e.g., databases with dates of coverage, contact with study authors to identify additional studies) in the search and date last searched -> **page 4**
8. Present full electronic search strategy for at least one database, including any limits used, such that it could be repeated -> **Supplementary data**
9. State the process for selecting studies (i.e., screening, eligibility, included in systematic review, and, if applicable, included in the meta-analysis) -> **page 5**
10. Describe method of data extraction from reports (e.g., piloted forms, independently, in duplicate) and any processes for obtaining and confirming data from investigators -> **page 4**
11. List and define all variables for which data were sought (e.g., PICOS, funding sources) and any assumptions and simplifications made -> **page 4**
12. Describe methods used for assessing risk of bias of individual studies (including specification of whether this was done at the study or outcome level), and how this information is to be used in any data synthesis -> **page 5-6**
13. State the principal summary measures (e.g., risk ratio, difference in means). Also describe the use of additional summary measures assessed, such as treatment rankings and surface under the cumulative ranking curve (SUCRA) values, as well as modified approaches used to present summary findings from meta-analyses -> **page 8-9**
14. Describe the methods of handling data and combining results of studies for each network meta-analysis. This should include, but not be limited to: handling of multi-arm trials; selection of variance structure; selection of prior distributions in Bayesian analyses; and assessment of model fit -> **page 8-9**
15. Specify any assessment of risk of bias that may affect the cumulative evidence (e.g., publication bias, selective reporting within studies) -> **page 6-7**
16. Describe methods of additional analyses if done, indicating which were pre-specified. This may include, but not be limited to, the following: sensitivity or subgroup analyses; meta-regression analyses; alternative formulations of the treatment network; and use of alternative prior distributions for Bayesian analyses (if applicable) -> **page 8-9**
17. Give numbers of studies screened, assessed for eligibility, and included in the review, with reasons for exclusions at each stage, ideally with a flow diagram -> **Supplementary data, Figure 1**
18. For each study, present characteristics for which data were extracted (e.g., study size, PICOS, follow-up period) and provide the citations -> **Supplementary data, Table 3-5 and references**
19. Present data on risk of bias of each study and, if available, any outcome level assessment -> **Supplementary data, Table 5**
20. For all outcomes considered (benefits or harms), present, for each study: 1) simple summary data for each intervention group, and 2) effect estimates and confidence intervals. Modified approaches may be needed to deal with information from larger networks -> **due to the large number of included studies and sensitive nature of our data, we are happy to supply requests for the original data used in this NMA once we have published our methods paper on the methodological issues identified in this review**
21. Present results of each meta-analysis done, including confidence/credible intervals. In larger networks, authors may focus on comparisons versus a particular comparator (e.g. placebo or standard care), with full findings presented in an appendix. League tables and forest plots may be considered to summarize pairwise comparisons. If additional summary measures were explored (such as treatment rankings), these should also be presented -> **Supplementary data**
22. Present results of any assessment of risk of bias across studies for the evidence base being studied -> **Supplementary data, Table 3 and CINeMA assessments**
23. Give results of additional analyses, if done (e.g., sensitivity or subgroup analyses, meta-regression analyses, alternative network geometries studied, alternative choice of prior distributions for Bayesian analyses, and so forth) -> **Supplementary data**
24. Summarize the main findings, including the strength of evidence for each main outcome; consider their relevance to key groups (e.g., healthcare providers, users, and policy- makers)

-> **page 14**

1. Discuss limitations at study and outcome level (e.g., risk of bias), and at review level (e.g., incomplete retrieval of identified research, reporting bias). Comment on the validity of the assumptions, such as transitivity and consistency. Comment on any concerns regarding network geometry (e.g., avoidance of certain comparisons) -> **page 15-16**
2. Provide a general interpretation of the results in the context of other evidence, and implications for future research -> **page 14-15**
3. Describe sources of funding for the systematic review and other support (e.g., supply of data); role of funders for the systematic review. This should also include information regarding whether funding has been received from manufacturers of treatments in the network and/or whether some of the authors are content experts with professional conflicts of interest that could affect use of treatments in the network -> **page 18**

**S1** Describe methods used to explore the geometry of the treatment network under study and potential biases related to it. This should include how the evidence base has been graphically summarized for presentation, and what characteristics were compiled and used to describe the evidence base to readers -> **Supplementary data, using network plot and network characteristics table**

**S2** Describe the statistical methods used to evaluate the agreement of direct and indirect evidence in the treatment network(s) studied. Describe efforts taken to address its presence when found -> **page 8-9**

**S3** Provide a network graph of the included studies to enable visualization of the geometry of the treatment network -> **Supplementary data and Figure 1 and 3, using network plots and network characteristics table**

**S4** Provide a brief overview of characteristics of the treatment network. This may include commentary on the abundance of trials and randomized patients for the different interventions and pairwise comparisons in the network, gaps of evidence in the treatment network, and potential biases reflected by the network structure -> **Supplementary data and page 10**

**S5** Describe results from investigations of inconsistency. This may include such information as measures of model fit to compare consistency and inconsistency models, P values from statistical tests, or summary of inconsistency estimates from different parts of the treatment network -> **Supplementary data**

# **Ongoing and unpublished clinical trials identified**

**NCT02271698**

| Type of surgery | TKA |
| --- | --- |
| Target/actual recruitment | 45 |
| Intervention | IV dexamethasone |
| Comparator | Placebo |
| Chronic pain outcomes | Severity CPSP at 3 and 6 months |
| Status (from investigator or trial database) | Completed. No reply from investigator. |

**NCT02112864**

| Type of surgery | Thoracotomy |
| --- | --- |
| Target/actual recruitment | 60 |
| Intervention | IV dexamethasone |
| Comparator | Placebo |
| Chronic pain outcomes | Incidence and severity of CPSP at 3 and 6 months |
| Status (from investigator or trial database) | Reply for investigator. Recruitment ongoing but closing early due to reduction in surgeries performed. |

**NCT01812057**

| Type of surgery | Caesarean section |
| --- | --- |
| Target/actual recruitment | 53 |
| Intervention | IV dexamethasone |
| Comparator | Placebo |
| Chronic pain outcomes | Incidence CPSP at 6 months |
| Status (from investigator or trial database) | Completed. No reply from investigator. |

**NCT01868633**

| Type of surgery | Caesarean section |
| --- | --- |
| Target/actual recruitment | 52 |
| Intervention | IV dexamethasone |
| Comparator | Placebo |
| Chronic pain outcomes | Incidence CPSP at 6 months |
| Status (from investigator or trial database) | Reply from investigator. Published but no chronic pain data collected. |

**NCT03527576**

| Type of surgery | Osteosynthesis |
| --- | --- |
| Target/actual recruitment | 50 |
| Intervention | IV dexamethasone |
| Comparator | Placebo |
| Chronic pain outcomes | Incidence and severity CPSP at 3 and 6 months |
| Status (from investigator or trial database) | Recently completed. |

**NCT02729805**

| Type of surgery | Breast |
| --- | --- |
| Target/actual recruitment | 135 |
| Intervention | IV ketamine |
| Comparator | Placebo |
| Chronic pain outcomes | Incidence and severity CPSP at 3 months |
| Status (from investigator or trial database) | Unknown. No reply from investigator. |

**NCT00129597**

| Type of surgery | Breast |
| --- | --- |
| Target/actual recruitment | 40 |
| Intervention | Ketamine |
| Comparator | Not reported |
| Chronic pain outcomes | Incidence and severity CPSP at 3 months |
| Status (from investigator or trial database) | Unknown. No reply from investigator. |

**NCT03090776**

| Type of surgery | Breast |
| --- | --- |
| Target/actual recruitment | 200 |
| Intervention | IV ketamine |
| Comparator | Placebo |
| Chronic pain outcomes | Severity CPSP at 2 years |
| Status (from investigator or trial database) | Recruiting. No reply from investigator. |

**NCT02527083**

| Type of surgery | Herniorrhaphy |
| --- | --- |
| Target/actual recruitment | 10 |
| Intervention | IV ketamine |
| Comparator | Not reported |
| Chronic pain outcomes | Severity CPSP at 1, 3, 6 and 12 months |
| Status (from investigator or trial database) | Completed. No contact information. |

**NCT02154438**

| Type of surgery | Spinal (lumbar) |
| --- | --- |
| Target/actual recruitment | 56 |
| Intervention | IV ketamine |
| Comparator | Placebo |
| Chronic pain outcomes | Severity CPSP at 3 months |
| Status (from investigator or trial database) | Recruiting. No reply from investigator. |

**ACTRN12617001619336 (ROCKet trial)**

| Type of surgery | Mixed |
| --- | --- |
| Target/actual recruitment | 4884 |
| Intervention | IV ketamine |
| Comparator | Placebo |
| Chronic pain outcomes | Incidence and severity CPSP at 3 and 12 months |
| Status (from investigator or trial database) | Ongoing, two-thirds of participants enrolled. |

**NCT01022840**

| Type of surgery | Abdominal |
| --- | --- |
| Target/actual recruitment | 60 |
| Intervention | IV S-ketamine |
| Comparator | Placebo |
| Chronic pain outcomes | Opioid consumption at 1 year |
| Status (from investigator or trial database) | Reply from investigator. Acute pain published but no chronic pain data. |

**NCT03105765**

| Type of surgery | Thoracotomy |
| --- | --- |
| Target/actual recruitment | 200 |
| Intervention | IV ketamine |
| Comparator | Placebo |
| Chronic pain outcomes | Incidence and severity CPSP at 3 months |
| Status (from investigator or trial database) | Completed. No contact information. |

**NCT03280017**

| Type of surgery | VATS |
| --- | --- |
| Target/actual recruitment | 32 |
| Intervention | IV ketamine |
| Comparator | Placebo |
| Chronic pain outcomes | Severity CPSP at 3 months |
| Status (from investigator or trial database) | Reply from investigator. Chronic pain data is incomplete. |

**EudraCT Number 2017-002616-13**

| Type of surgery | Craniotomy |
| --- | --- |
| Target/actual recruitment | 62 |
| Intervention | IV S-ketamine |
| Comparator | Placebo |
| Chronic pain outcomes | Unclear if chronic pain included |
| Status (from investigator or trial database) | Ongoing |

**EudraCT Number 2011-004733-14**

| Type of surgery | Major orthopaedic |
| --- | --- |
| Target/actual recruitment | 60 |
| Intervention | IV S-ketamine |
| Comparator | Placebo |
| Chronic pain outcomes | Opioid use at 3 and 6 months |
| Status (from investigator or trial database) | Completed early. No reply from investigator. |

**EudraCT number 2008-007987-40**

| Type of surgery | Thoracotomy |
| --- | --- |
| Target/actual recruitment | 66 |
| Intervention | IV S-ketamine |
| Comparator | Placebo |
| Chronic pain outcomes | Incidence of CPSP at 6 months |
| Status (from investigator or trial database) | Completed. No contact information. |

**ChiCTR2100042239**

| Type of surgery | Thoracoscopic lung resection |
| --- | --- |
| Target/actual recruitment | 146 |
| Intervention | IV S-ketamine |
| Comparator | Placebo |
| Chronic pain outcomes | Severity CPSP at 3 and 6 months |
| Status (from investigator or trial database) | Recruiting. No reply from investigator. |

**ChiCTR2100042140**

| Type of surgery | Thoracoscopic lung resection |
| --- | --- |
| Target/actual recruitment | 434 |
| Intervention | IV S-ketamine |
| Comparator | Placebo |
| Chronic pain outcomes | Incidence and severity CPSP at 3 months |
| Status (from investigator or trial database) | Completed. No reply from investigator. |

**ChiCTR2000040012**

| Type of surgery | Thoracoscopic lung resection |
| --- | --- |
| Target/actual recruitment | 894 |
| Intervention | IV S-ketamine |
| Comparator | Placebo |
| Chronic pain outcomes | Severity CPSP at 3 months |
| Status (from investigator or trial database) | Recruiting. No reply from investigator. |

**ChiCTR2000035373**

| Type of surgery | Thoracoscopic surgery |
| --- | --- |
| Target/actual recruitment | 200 |
| Intervention | IV ketamine |
| Comparator | Placebo |
| Chronic pain outcomes | No details |
| Status (from investigator or trial database) | Not yet recruiting. No reply from investigator. |

**NCT03158376**

| Type of surgery | Thoracotomy |
| --- | --- |
| Target/actual recruitment | 238 |
| Intervention | PO gabapentin |
| Comparator | Placebo |
| Chronic pain outcomes | Incidence of CPSP at 3 months |
| Status (from investigator or trial database) | Unknown. No reply from investigator. |

**NCT00663962**

| Type of surgery | Thoracotomy |
| --- | --- |
| Target/actual recruitment | 15 |
| Intervention | PO pregabalin |
| Comparator | Placebo |
| Chronic pain outcomes | Incidence of CPSP at 4 and 6 months |
| Status (from investigator or trial database) | Completed. No contact information. |

**NCT00934193**

| Type of surgery | Thoracotomy |
| --- | --- |
| Target/actual recruitment | 0 |
| Intervention | PO gabapentin |
| Comparator | Placebo |
| Chronic pain outcomes | Incidence of CPSP at 6 months |
| Status (from investigator or trial database) | Withdrawn. |

**ISRCTN63614165 (GAP trial)**

| Type of surgery | Mixed |
| --- | --- |
| Target/actual recruitment | 1196 |
| Intervention | PO gabapentin |
| Comparator | Placebo |
| Chronic pain outcomes | Severity of CPSP at 4 months |
| Status (from investigator or trial database) | Reply from investigator. Ongoing recruitment, delayed due to COVID-19 pandemic. |

**NCT00631891/NCT01391858**

| Type of surgery | Breast |
| --- | --- |
| Target/actual recruitment | 80 |
| Intervention | PO pregabalin |
| Comparator | Placebo |
| Chronic pain outcomes | Severity CPSP at 3 months |
| Status (from investigator or trial database) | Completed. No reply from investigator. |

**NCT00852683**

| Type of surgery | Breast |
| --- | --- |
| Target/actual recruitment | 70 |
| Intervention | PO pregabalin |
| Comparator | Placebo |
| Chronic pain outcomes | Incidence CPSP at 3 months |
| Status (from investigator or trial database) | Completed. No reply from investigator. |

**NCT02306278**

| Type of surgery | Neurosurgery |
| --- | --- |
| Target/actual recruitment | 122 |
| Intervention | PO gabapentin |
| Comparator | Vitamin capsule |
| Chronic pain outcomes | Incidence and severity of CPSP at 3 and 6 months |
| Status (from investigator or trial database) | Completed. No reply from investigator. |

**NCT03714867**

| Type of surgery | Head and neck cancer resection |
| --- | --- |
| Target/actual recruitment | 0 |
| Intervention | PO pregabalin |
| Comparator | Placebo |
| Chronic pain outcomes | Severity of CPSP at 2 years |
| Status (from investigator or trial database) | Withdrawn. Unable to recruit. |

**RBR-555xm5**

| Type of surgery | Breast |
| --- | --- |
| Target/actual recruitment | 45 |
| Intervention | PO pregabalin |
| Comparator | Placebo |
| Chronic pain outcomes | Unclear |
| Status (from investigator or trial database) | No reply from investigator. |

**EudraCT number 2007-005996-32**

| Type of surgery | Spinal surgery |
| --- | --- |
| Target/actual recruitment | 60 |
| Intervention | PO gabapentin |
| Comparator | Placebo |
| Chronic pain outcomes | Severity of CPSP (unclear time-point) |
| Status (from investigator or trial database) | Prematurely ended. No contact information |

**RBR-3pgyyq**

| Type of surgery | Inguinal hernia repair |
| --- | --- |
| Target/actual recruitment | 60 |
| Intervention | PO pregabalin |
| Comparator | Placebo |
| Chronic pain outcomes | Incidence of CPSP at 3 months |
| Status (from investigator or trial database) | Reply from investigator. Trial stopped before recruitment. |

**CTRI/2016/10/007397**

| Type of surgery | Breast |
| --- | --- |
| Target/actual recruitment | 100 |
| Intervention | PO pregabalin |
| Comparator | Placebo |
| Chronic pain outcomes | Incidence of CPSP at 3 months |
| Status (from investigator or trial database) | No reply from investigator. |

**ACTRN12608000471381**

| Type of surgery | Thoracotomy |
| --- | --- |
| Target/actual recruitment | Unclear |
| Intervention | PO gabapentin |
| Comparator | Unclear |
| Chronic pain outcomes | Unclear |
| Status (from investigator or trial database) | Not yet recruiting. No reply from investigator. |

**NCT02862769**

| Type of surgery | VATS |
| --- | --- |
| Target/actual recruitment | 120 |
| Intervention | IV lidocaine |
| Comparator | Placebo |
| Chronic pain outcomes | Severity of CPSP at 3 and 6 months |
| Status (from investigator or trial database) | Unknown. No reply from investigator. |

**NCT02894710**

| Type of surgery | ENT cancer surgery |
| --- | --- |
| Target/actual recruitment | 143 |
| Intervention | IV lidocaine |
| Comparator | Placebo |
| Chronic pain outcomes | Severity of CPSP at 3 and 6 months |
| Status (from investigator or trial database) | Completed. Attempting to publish as a letter. |

**NCT03673163**

| Type of surgery | Inguinal hernia repair |
| --- | --- |
| Target/actual recruitment | 180 |
| Intervention | IV lidocaine |
| Comparator | Placebo |
| Chronic pain outcomes | Incidence CPSP at 3, 6 and 12 months |
| Status (from investigator or trial database) | Reply from investigator. Trial suspended due to too few surgeries. |

**NCT03666299**

| Type of surgery | Thoracotomy |
| --- | --- |
| Target/actual recruitment | 72 |
| Intervention | IV lidocaine |
| Comparator | Placebo |
| Chronic pain outcomes | Incidence of CPSP at 3 and 6 months |
| Status (from investigator or trial database) | Unknown. No reply from investigator. |

**NCT03677817**

| Type of surgery | VATS |
| --- | --- |
| Target/actual recruitment | 113 |
| Intervention | IV lidocaine |
| Comparator | Placebo |
| Chronic pain outcomes | Severity of CPSP at 3 and 6 months |
| Status (from investigator or trial database) | Reply from investigator. Study finished but not willing to disclose results until published. |

**NCT02786329**

| Type of surgery | Colorectal cancer |
| --- | --- |
| Target/actual recruitment | 450 |
| Intervention | IV lidocaine |
| Comparator | Placebo |
| Chronic pain outcomes | Severity CPSP at 6 months and 1 year |
| Status (from investigator or trial database) | Recruiting. Reply from investigator. No results as yet. |

**ACTRN12621000589886**

| Type of surgery | Gynaecology laparoscopy |
| --- | --- |
| Target/actual recruitment | 70 |
| Intervention | IV lidocaine |
| Comparator | Unclear |
| Chronic pain outcomes | Unclear if CPSP measured |
| Status (from investigator or trial database) | Reply from investigator. Recruitment ongoing. |

**ACTRN12620000203954**

| Type of surgery | Caesarean section |
| --- | --- |
| Target/actual recruitment | 260 |
| Intervention | IV lidocaine |
| Comparator | Placebo |
| Chronic pain outcomes | Incidence of CPSP at 3 months |
| Status (from investigator or trial database) | Recruiting. No reply from investigator. |

**NCT04874038 (PLAN trial)**

| Type of surgery | Breast |
| --- | --- |
| Target/actual recruitment | 1150 |
| Intervention | IV lidocaine |
| Comparator | Placebo |
| Chronic pain outcomes | Incidence and severity of CPSP and opioid consumption at 3 and 12 months |
| Status (from investigator or trial database) | Ongoing recruitment. |

**NCT00413257**

| Type of surgery | Cardiac |
| --- | --- |
| Target/actual recruitment | 90 |
| Intervention | IV nefopam |
| Comparator | Placebo |
| Chronic pain outcomes | Severity of CPSP at 3, 6 and 12 months |
| Status (from investigator or trial database) | Completed. No reply from investigator. |

**KCT0006246**

| Type of surgery | VATS |
| --- | --- |
| Target/actual recruitment | 100 |
| Intervention | IV nefopam |
| Comparator | Placebo |
| Chronic pain outcomes | Severity of CPSP at 3 months |
| Status (from investigator or trial database) | Recently published. |

**NCT04700592**

| Type of surgery | Inguinal hernia repair |
| --- | --- |
| Target/actual recruitment | 60 |
| Intervention | PO gabapentin and IV ketamine |
| Comparator | NA |
| Chronic pain outcomes | Severity of CPSP at 3 months |
| Status (from investigator or trial database) | Completed. No reply from investigator. |

**ChiCTR2000038124**

| Type of surgery | Breast |
| --- | --- |
| Target/actual recruitment | 100 |
| Intervention | IV S-ketamine and PO pregabalin |
| Comparator | Placebo |
| Chronic pain outcomes | Incidence of CPSP at 3 and 6 months |
| Status (from investigator or trial database) | Not yet recruiting. No reply from investigator. |

**EudraCT number 2012-002518-38**

| Type of surgery | Spinal |
| --- | --- |
| Target/actual recruitment | 128 |
| Intervention | IV ketamine and dexamethasone |
| Comparator | Placebo |
| Chronic pain outcomes | Severity of CPSP at 3 months |
| Status (from investigator or trial database) | No reply from investigator. |

**NCT01789216**

| Type of surgery | Orthopaedic |
| --- | --- |
| Target/actual recruitment | 450 |
| Intervention | NSAIDs and PO pregabalin |
| Comparator | Placebo |
| Chronic pain outcomes | Incidence and severity of CPSP at 3, 6 and 12 months and opioid use at 1 year |
| Status (from investigator or trial database) | Reply from investigator. Results published this year. |

**NCT03275207**

| Type of surgery | Breast and thoracic |
| --- | --- |
| Target/actual recruitment | 120 |
| Intervention | IV dexmedetomidine |
| Comparator | Placebo |
| Chronic pain outcomes | Severity of CPSP at 3, 6 and 12 months |
| Status (from investigator or trial database) | Unknown. No reply from investigator. |

**NCT03480061**

| Type of surgery | Cardiac |
| --- | --- |
| Target/actual recruitment | 90 |
| Intervention | IV dexmedetomidine |
| Comparator | Usual care |
| Chronic pain outcomes | Severity of CPSP at 3, 6 and 12 months |
| Status (from investigator or trial database) | Reply from investigator. Pilot study for NCT04289142 so will be published with that study. |

**NCT04289142 (CODEX trial)**

| Type of surgery | Cardiac |
| --- | --- |
| Target/actual recruitment | 2400 |
| Intervention | IV dexmedetomidine |
| Comparator | Usual care |
| Chronic pain outcomes | Severity of CPSP at 3, 6 and 12 months |
| Status (from investigator or trial database) | Reply from investigator. Recruiting. |

**TCTR20140716001**

| Type of surgery | Shoulder arthroscopy |
| --- | --- |
| Target/actual recruitment | 86 |
| Intervention | IV parecoxib |
| Comparator | Placebo |
| Chronic pain outcomes | Unclear |
| Status (from investigator or trial database) | Reply from investigator. Study terminated early with no results available. |

**NCT04622904**

| Type of surgery | Gynaecology surgery |
| --- | --- |
| Target/actual recruitment | 90 |
| Intervention | 1) IV lidocaine  2) IV lidocaine and ketamine  3) IV lidocaine and magnesium |
| Comparator | NA |
| Chronic pain outcomes | Incidence of CPSP at 3 months |
| Status (from investigator or trial database) | Recruiting. No reply from investigator. |

**NCT02450214**

| Type of surgery | Abdominal plastic surgery |
| --- | --- |
| Target/actual recruitment | 63 |
| Intervention | 1) IV ketamine  2) IV ketamine and magnesium |
| Comparator | Placebo |
| Chronic pain outcomes | Severity of CPSP at 3 months. |
| Status (from investigator or trial database) | Completed. No reply from investigator. |

**NCT03063931**

| Type of surgery | Breast |
| --- | --- |
| Target/actual recruitment | 100 |
| Intervention | IV magnesium |
| Comparator | Placebo |
| Chronic pain outcomes | CPSP at 3 months |
| Status (from investigator or trial database) | Unknown. No reply from investigator. |

**ACTRN12617000830392**

| Type of surgery | Thoracotomy |
| --- | --- |
| Target/actual recruitment | 200 |
| Intervention | IV magnesium |
| Comparator | Placebo |
| Chronic pain outcomes | Incidence and severity of CPSP at 6 months |
| Status (from investigator or trial database) | Recruiting. No reply from investigator. |

# **References for included studies**

**Acin 2009**

Acin MP, Bono MC, Rodrigo MD, Martinez R, Faci A, Escartin R. Preventive analgesia with pregabalin in mesh hernia repair. Review at 1 year. *Rev Soc Esp Dolor* 2009; 16: 215.

**Ahiskalioglu 2016**

Ahiskalioglu EO, Ahiskalioglu A, Aydin P, Arslan Z, Aksoy M, Temiz A. Effects of a single-dose preemptive pregabalin on acute and chronic pain after inguinal hernia repair with mesh under spinal anaesthesia: a randomised controlled trial. *European Journal of Anaesthesiology* 2016; 33(8): 605-7.

**Alzeftawy 2015**

Alzeftawy AE, Elsheikh NA. Evaluation of the efficacy of dexmedetomidine infusion on the quality of balanced anesthesia and postmastectomy pain. *Research and Opinion in Anesthesia and Intensive Care* 2015; 2(3): 73.

**Amr 2010**

Amr YM, Yousef AA. Evaluation of efficacy of the perioperative administration of venlafaxine or gabapentin on acute and chronic postmastectomy pain. *The Clinical Journal of Pain* 2010; 26(5): 381-5.

**Anwar 2019**

Anwar S, Cooper J, Rahman J, Sharma C, Langford R. Prolonged perioperative use of pregabalin and ketamine to prevent persistent pain after cardiac surgery. *Anesthesiology* 2019; 131(1): 119-31.

**Aveline 2014**

Aveline C, Le Roux A, Le Hetet H, Gautier JF, Vautier P, Cognet F, Bonnet F. Pain and recovery after total knee arthroplasty: a 12-month follow-up after a prospective randomized study evaluating nefopam and ketamine for early rehabilitation. *The Clinical Journal of Pain* 2014; 30(9): 749-54.

**Bakr 2014**

Bakr M, Khalil AA, Fares KM, Mohamed SA, Abdel-Rahman AM, Doheim AM. The effect of ketamine infusion on post mastectomy pain syndrome: a randomized controlled study. *SECI Oncology* 2014: 25-34.

**Beaussier 2018**

Beaussier M, Parc Y, Guechot J, Cachanado M, Rousseau A, Lescot T, CATCH Study Investigators, Rollin M, Aissou M, Lefevre JH, Restoux A. Ropivacaine preperitoneal wound infusion for pain relief and prevention of incisional hyperalgesia after laparoscopic colorectal surgery: a randomized, triple‐arm, double‐blind controlled evaluation vs intravenous lidocaine infusion, the CATCH study. *Colorectal Disease* 2018; 20(6): 509-19.

**Bergeron 2009**

Bergeron SG, Kardash KJ, Huk OL, Zukor DJ, Antoniou J. Perioperative dexamethasone does not affect functional outcome in total hip arthroplasty. *Clinical Orthopaedics and Related Research* 2009; 467(6): 1463-7.

**Bielka 2018**

Bielka K, Kuchyn I, Babych V, Martycshenko K, Inozemtsev O. Dexmedetomidine infusion as an analgesic adjuvant during laparoscopic cholecystectomy: a randomized controlled study. *BMC Anesthesiology* 2018; 18(1): 1-6.

**Bilgen 2012**

Bilgen S, Köner O, Türe H, Menda F, Fiçicioğlu C, Aykaç B. Effect of three different doses of ketamine prior to general anaesthesia on postoperative pain following caesarean delivery: a prospective randomized study. *Minerva Anestesiologica* 2012; 78(4): 442-9.

**Borys 2020**

Borys M, Hanych A, Czuczwar M. Paravertebral block versus preemptive ketamine effect on pain intensity after posterolateral thoracotomies: a randomized controlled trial. *Journal of Clinical Medicine* 2020; 9(3): 793.

**Bouzia 2017**

Bouzia A, Tassoudis V, Karanikolas M, Vretzakis G, Petsiti A, Tsilimingas N, Arnaoutoglou E. Pregabalin effect on acute and chronic pain after cardiac surgery. *Anesthesiology Research and Practice* 2017; 2017: 2753962.

**Brinck 2020**

Brinck EC, Maisniemi K, Kankare J, Tielinen L, Tarkkila P, Kontinen VK. Analgesic effect of intraoperative intravenous S-ketamine in opioid-naïve patients after major lumbar fusion surgery is temporary and not dose-dependent: a randomized, double-blind, placebo-controlled clinical trial. *Anesthesia & Analgesia* 2020; 132(1): 69-79.

**Brogly 2008**

Brogly N, Wattier JM, Andrieu G, Peres D, Robin E, Kipnis E, Arnalsteen L, Thielemans B, Carnaille B, Pattou F, Vallet B. Gabapentin attenuates late but not early postoperative pain after thyroidectomy with superficial cervical plexus block. *Anesthesia & Analgesia* 2008; 107(5): 1720-5.

**Brulotte 2015**

Brulotte V, Ruel MM, Lafontaine E, Chouinard P, Girard F. Impact of pregabalin on the occurrence of postthoracotomy pain syndrome: a randomized trial. *Regional Anesthesia & Pain Medicine* 2015; 40(3): 262-9.

**Burke 2010**

Burke SM, Shorten GD. Perioperative pregabalin improves pain and functional outcomes 3 months after lumbar discectomy. *Anesthesia & Analgesia* 2010; 110(4): 1180-5.

**Buvanendran 2010**

Buvanendran A, Kroin JS, Della Valle CJ, Kari M, Moric M, Tuman KJ. Perioperative oral pregabalin reduces chronic pain after total knee arthroplasty: a prospective, randomized, controlled trial. *Anesthesia & Analgesia* 2010; 110(1): 199-207.

**Cameron 2020**

Cameron M, Tam K, Al Wahaibi K, Charghi R, Béïque F. Intraoperative ketamine for analgesia post-coronary artery bypass surgery: a randomized, controlled, double-blind clinical trial. *Journal of Cardiothoracic and Vascular Anesthesia* 2020; 34(3): 586-91.

**Çelebi 2013**

Çelebi T, Kocamanoglu IS, Üstün YB, Üstün E, Sahinoglu H. The effects of preemptive ketamine and gabapentin on volatile agent consumption, postoperative analgesic requirement and chronic pain. *Turkish Journal of Anaesthesiology & Reanimation* 2013; 41(2): 38.

**Chan 2020**

Chan TC, Cheung CW, Wong SS, Chung AY, Irwin MG, Chan PK, Fu H, Yan CH, Chiu KY. Preoperative dexamethasone for pain relief after total knee arthroplasty: a randomised controlled trial. *European Journal of Anaesthesiology* 2020; 37(12): 1157-67.

**Chaparro 2010**

Chaparro LE, Muñoz Pérez Y, Gallo CA, Álvarez HA, Restrepo Restrepo SM, Pérez N, Restrepo L. Pain and sensory symptoms following augmentation mammoplasty: a long term follow-up study with intraoperative ketamine use. *Colombian Journal of Anestesiology* 2010; 38(2): 204-12.

**Choi 2013**

Choi YS, Shim JK, Song JW, Kim JC, Yoo YC, Kwak YL. Combination of pregabalin and dexamethasone for postoperative pain and functional outcome in patients undergoing lumbar spinal surgery: a randomized placebo-controlled trial. *The Clinical Journal of Pain* 2013; 29(1): 9-14.

**Choi 2017**

Choi KW, Nam KH, Lee JR, Chung WY, Kang SW, Joe YE, Lee JH. The effects of intravenous lidocaine infusions on the quality of recovery and chronic pain after robotic thyroidectomy: a randomized, double-blinded, controlled study. *World Journal of Surgery* 2017; 41(5): 1305-12.

**Chumbley 2019**

Chumbley GM, Thompson L, Swatman JE, Urch C. Ketamine infusion for 96 hr after thoracotomy: effects on acute and persistent pain. *European Journal of Pain* 2019; 23(5): 985-93.

**Clarke 2009**

Clarke H, Pereira S, Kennedy D, Andrion J, Mitsakakis N, Gollish J, Katz J, Kay J. Adding gabapentin to a multimodal regimen does not reduce acute pain, opioid consumption or chronic pain after total hip arthroplasty. *Acta Anaesthesiologica Scandinavica* 2009; 53(8): 1073-83.

**Clarke 2014**

Clarke HA, Katz J, McCartney CJ, Stratford P, Kennedy D, Pagé MG, Awad IT, Gollish J, Kay J. Perioperative gabapentin reduces 24 h opioid consumption and improves in-hospital rehabilitation but not post-discharge outcomes after total knee arthroplasty with peripheral nerve block. *British Journal of Anaesthesia* 2014; 113(5): 855-64.

**Clarke 2015**

Clarke H, Pagé GM, McCartney CJ, Huang A, Stratford P, Andrion J, Kennedy D, Awad IT, Gollish J, Kay J, Katz J. Pregabalin reduces postoperative opioid consumption and pain for 1 week after hospital discharge, but does not affect function at 6 weeks or 3 months after total hip arthroplasty. *British Journal of Anaesthesia* 2015; 115(6): 903-11.

**Comez 2015**

Comez M, Celik M, Dostbil A, Aksoy M, Ahiskalioglu A, Erdem AF, Aydin Y, İnce İ. The effect of pre-emptive intravenous dexketoprofen and thoracic epidural analgesia on the chronic post-thoracotomy pain. *International Journal of Clinical and Experimental Medicine* 2015; 8(5): 8101.

**Crousier 2008**

Crousier M, Cognet V, Khaled M, Gueugniaud PY, Piriou V. Effect of ketamine on prevention of postmastectomy chronic pain. a pilot study. *Annales Francaises D'anesthesie et de Reanimation* 2008; 27(12): 987-993.

**Czarnetzki 2020**

Czarnetzki C, Desmeules J, Tessitore E, Faundez A, Chabert J, Daali Y, Fournier R, Dupuis‐Lozeron E, Cedraschi C, Richard Tramèr M. Perioperative intravenous low‐dose ketamine for neuropathic pain after major lower back surgery: a randomized, placebo‐controlled study. *European Journal of Pain* 2020; 24(3): 555-67.

**Dai 2020**

Dai Y, Jiang R, Su W, Zuo Y, Wang M, Liu Y. Impact of perioperative intravenous lidocaine infusion on postoperative pain and rapid recovery of patients undergoing gastrointestinal tumor surgery: a randomized, double-blind trial. *Journal of Gastrointestinal Oncology* 2020; 11 (6): 1274.

**De Kock 2001**

De Kock M, Lavand'homme P, Waterloos H. ‘Balanced analgesia’in the perioperative period: is there a place for ketamine? *Pain* 2001; 92(3): 373-80.

**Dhir 2020**

Dhir S, Roy D, Hall T, Bureau Y, Yu J, Dhir AK. Relative contributions of intraoperative low dose ketamine, lidocaine and ketamine-lidocaine combination in addition to intrathecal morphine for postoperative analgesia in open liver resection: a prospective, randomized, four-arm, triple blind, placebo-controlled trial. *Open Journal of Anesthesiology* 2020; 10(09): 313.

**Dualé 2009**

Dualé C, Sibaud F, Guastella V, Vallet L, Gimbert YA, Taheri H, Filaire M, Schoeffler P, Dubray C. Perioperative ketamine does not prevent chronic pain after thoracotomy. *European Journal of Pain* 2009; 13(5): 497-505.

**Dullenkopf 2009**

Dullenkopf A, Müller R, Dillmann F, Wiedemeier P, Hegi TR, Gautschi S. An intraoperative pre-incision single dose of intravenous ketamine does not have an effect on postoperative analgesic requirements under clinical conditions. *Anaesthesia and Intensive Care* 2009; 37(5): 753-7.

**Fassoulaki 2002**

Fassoulaki A, Patris K, Sarantopoulos C, Hogan Q. The analgesic effect of gabapentin and mexiletine after breast surgery for cancer. *Anesthesia & Analgesia* 2002; 95(4): 985-91.

**Fassoulaki 2012**

Fassoulaki A, Melemeni A, Tsaroucha A, Paraskeva A. Perioperative pregabalin for acute and chronic pain after abdominal hysterectomy or myomectomy: a randomised controlled trial. *European Journal of Anaesthesiology* 2012; 29(11): 531-6.

**Fawzi 2014**

Fawzi HM, El-Tohamy SA. Effect of perioperative oral pregabalin on the incidence of post-thoracotomy pain syndrome. *Ain-Shams Journal of Anaesthesiology* 2014; 7(2): 143.

**Fransen 2006**

Fransen M, Anderson C, Douglas J, MacMahon S, Neal B, Norton R, Woodward M, Cameron ID, Crawford R, Lo SK, Tregonning G. Safety and efficacy of routine postoperative ibuprofen for pain and disability related to ectopic bone formation after hip replacement surgery (HIPAID): randomised controlled trial. *British Medical Journal* 2006; 333(7567): 519.

**Gaber 2019**

Gaber S, Saleh E, Elshaikh S, Reyad R, Elramly M, Mourad I, Fattah MA. Role of perioperative pregabalin in the management of acute and chronic post-thoracotomy pain. *Open Access Macedonian Journal of Medical Sciences* 2019; 7(12): 1974.

**Galinski 2007**

Galinski SF, Pereira JA, Maestre Y, Francés S, Escolano F, Puig MM. The combination of intravenous dexamethasone and ketamine does not improve postoperative analgesia when compared to each drug individually. *The Pain Clinic* 2007; 19(5): 223-9.

**Galos 2020**

Galoş EV, Tat TF, Popa R, Vesa ŞC, Vasian H, Ionescu DC, Mihu CM. Continuous intravenous infusion of lidocaine for postoperative pain in breast cancer surgery patients. Effect on acute and chronic pain. *Human and Veterinary Medicine* 2020; 12(4): 149-56.

**Ge 2021** (*unpublished*)

Ge X, Pan Y, Jin D, Wang Y, Ge S. Impact of perioperative use of parecoxib on chronic postsurgical pain in elderly patients undergoing hepatectomy: a prospective randomized controlled study. (*unpublished*)

**Ghimire 2020**

Ghimire A, Subedi A, Bhattarai B, Sah BP. The effect of intraoperative lidocaine infusion on opioid consumption and pain after totally extraperitoneal laparoscopic inguinal hernioplasty: a randomized controlled trial. *BMC Anesthesiology* 2020; 20(1): 1-8.

**Gianesello 2012**

Gianesello L, Pavoni V, Barboni E, Galeotti I, Nella A. Perioperative pregabalin for postoperative pain control and quality of life after major spinal surgery. *Journal of Neurosurgical Anesthesiology* 2012; 24(2): 121-6.

**Grigoras 2012**

Grigoras A, Lee P, Sattar F, Shorten G. Perioperative intravenous lidocaine decreases the incidence of persistent pain after breast surgery. *The Clinical Journal of Pain* 2012; 28(7): 567-72.

**Grosen 2014**

Grosen K, Drewes AM, Højsgaard A, Pfeiffer-Jensen M, Hjortdal VE, Pilegaard HK. Perioperative gabapentin for the prevention of persistent pain after thoracotomy: a randomized controlled trial. *European Journal of Cardio-Thoracic Surgery* 2014; 46(1): 76-85.

**Haddad 2019**

Haddad F, Jaoua H, Mrabet A, Bousselmi J, Fadhel KB. Parécoxib en chirurgie laparoscopique pour lithiase vésiculaire simple. Parecoxib in laparoscopic surgery for simple vesicular lithiasis. *La Tunisie Medicale* 2019; 97(12): 1338-1344.

**Hah 2018**

Hah J, Mackey SC, Schmidt P, McCue R, Humphreys K, Trafton J, Efron B, Clay D, Sharifzadeh Y, Ruchelli G, Goodman S. Effect of perioperative gabapentin on postoperative pain resolution and opioid cessation in a mixed surgical cohort: a randomized clinical trial. *JAMA Surgery* 2018; 153(4): 303-11.

**Han 2019a**

Han C, Lei D, Jiang W, Ren H, Su G, Feng S, Ge Z, Ma T. Pre-emptive dexmedetomidine decreases the incidence of chronic post hysterectomy pain. *International Journal of Clinical and Experimental Medicine* 2019; 12(1): 967-71.

**Han 2019b** (*unpublished*)

The effect of low dose ketamine on postoperative quality of recovery in patients undergoing breast cancer surgery: a randomized, placebo controlled trial (*unpublished*).

**Hayes 2004**

Hayes C, Armstrong-Brown A, Burstal R. Perioperative intravenous ketamine infusion for the prevention of persistent post-amputation pain: a randomized, controlled trial. *Anaesthesia and Intensive Care* 2004; 32(3): 330-8.

**Homma 2019**

Homma T, Doki Y, Yamamoto Y, Ojima T, Shimada Y, Kitamura N, Akemoto Y, Hida Y, Yoshimura N. Efficacy of 50 mg pregabalin for prevention of postoperative neuropathic pain after video-assisted thoracoscopic surgery and thoracotomy: a 3-month prospective randomized controlled trial. *Journal of Thoracic Disease* 2019; 11(3): 694.

**Hu 2014**

Hu J, Liao Q, Zhang F, Tong J, Ouyang W. Chronic postthoracotomy pain and perioperative ketamine infusion. *Journal of Pain & Palliative Care Pharmacotherapy* 2014; 28(2): 117-21.

**Ibrahim 2018**

Ibrahim A, Aly M, Farrag W. Effect of intravenous lidocaine infusion on long-term postoperative pain after spinal fusion surgery. *Medicine* 2018; 97(13).

**Jain 2012**

Jain G, Bansal P, Ahmad B, Singh DK, Yadav G. Effect of the perioperative infusion of dexmedetomidine on chronic pain after breast surgery. *Indian Journal of Palliative Care* 2012; 18(1): 45.

**Jendoubi 2017**

Jendoubi A, Naceur IB, Bouzouita A, Trifa M, Ghedira S, Chebil M, Houissa M. A comparison between intravenous lidocaine and ketamine on acute and chronic pain after open nephrectomy: A prospective, double-blind, randomized, placebo-controlled study. *Saudi Journal of Anaesthesia* 2017; 11(2): 177.

**Jeyamohan 2015**

Jeyamohan SB, Kenning TJ, Petronis KA, Feustel PJ, Drazin D, DiRisio DJ. Effect of steroid use in anterior cervical discectomy and fusion: a randomized controlled trial. *Journal of Neurosurgery: Spine* 2015; 23(2): 137-43.

**Joseph 2012**

Joseph C, Gaillat F, Duponq R, Lieven R, Baumstarck K, Thomas P, Penot-Ragon C, Kerbaul F. Is there any benefit to adding intravenous ketamine to patient-controlled epidural analgesia after thoracic surgery? a randomized double-blind study. *European Journal of Cardio-Thoracic Surgery* 2012; 42(4): e58-65.

**Joshi 2013**

Joshi SS, Jagadeesh AM. Efficacy of perioperative pregabalin in acute and chronic post-operative pain after off-pump coronary artery bypass surgery: a randomized, double-blind placebo controlled trial. *Annals of Cardiac Anaesthesia* 2013; 16(3): 180.

**Kang 2020**

Kang C, Cho AR, Kim KH, Eun-A L, Lee HJ, Jae-Young K, Kim H, Kim E, Baik JS, Kim C. Effects of intraoperative low-dose ketamine on persistent postsurgical pain after breast cancer surgery: a prospective, randomized, controlled, double-blind study. *Pain Physician* 2020; 23(1): 37.

**Katz 2004**

Katz J, Schmid R, Snijdelaar DG, Coderre TJ, McCartney CJ, Wowk A. Pre-emptive analgesia using intravenous fentanyl plus low-dose ketamine for radical prostatectomy under general anesthesia does not produce short-term or long-term reductions in pain or analgesic use. *Pain* 2004; 110(3):707-18.

**Kendall 2018**

Kendall MC, McCarthy RJ, Panaro S, Goodwin E, Bialek JM, Nader A, De Oliveira Jr GS. The effect of intraoperative systemic lidocaine on postoperative persistent pain using initiative on methods, measurement, and pain assessment in clinical trials criteria assessment following breast cancer surgery: a randomized, double‐blind, placebo‐controlled trial. *Pain Practice* 2018; 18(3): 350-9.

**Khan 2019**

Khan JS, Hodgson N, Choi S, Reid S, Paul JE, Hong NJ, Holloway C, Busse JW, Gilron I, Buckley DN, McGillion M. Perioperative pregabalin and intraoperative lidocaine infusion to reduce persistent neuropathic pain after breast cancer surgery: a multicentre, factorial, randomized, controlled pilot trial. *The Journal of Pain* 2019; 20(8): 980-93.

**Khurana 2014**

Khurana G, Jindal P, Sharma JP, Bansal KK. Postoperative pain and long-term functional outcome after administration of gabapentin and pregabalin in patients undergoing spinal surgery. *Spine* 2014; 39(6): E363.

**Kim 2010**

Kim SY, Jeong JJ, Chung WY, Kim HJ, Nam KH, Shim YH. Perioperative administration of pregabalin for pain after robot-assisted endoscopic thyroidectomy: a randomized clinical trial. *Surgical Endoscopy* 2010; 24(11): 2776-81.

**Kim 2017**

Kim MH, Lee KY, Park S, Kim SI, Park HS, Yoo YC. Effects of systemic lidocaine versus magnesium administration on postoperative functional recovery and chronic pain in patients undergoing breast cancer surgery: a prospective, randomized, double-blind, comparative clinical trial. *PLOS One* 2017; 12(3): e0173026.

**Kim 2018**

Kim BG, Moon JY, Choi JY, Park IS, Oh AY, Jeon YT, Hwang JW, Ryu JH. The effect of intraoperative nefopam administration on acute postoperative pain and chronic discomfort after robotic or endoscopic assisted thyroidectomy: a randomized clinical trial. *World Journal of Surgery* 2018; 42(7): 2094-101.

**Kinney 2012**

Kinney MA, Mantilla CB, Carns PE, Passe MA, Brown MJ, Michael Hooten W, Curry TB, Long TR, Thomas Wass C, Wilson PR, Weingarten TN. Preoperative gabapentin for acute post‐thoracotomy analgesia: a randomized, double‐blinded, active placebo‐controlled study. *Pain Practice* 2012; 12(3): 175-83.

**Konstantatos 2016**

Konstantatos AH, Howard W, Story D, Mok LY, Boyd D, Chan MT. A randomised controlled trial of peri‐operative pregabalin vs. placebo for video‐assisted thoracoscopic surgery. *Anaesthesia* 2016; 71(2): 192-7.

**Koşucu 2014**

Koşucu M, Tuğcugil E, Ertürk E, Topbaş M, Eroğlu A, Ulusoy H, Tekinbaş C. The effects of the pre-emptive oral gabapentin on post-anesthesia recovery criteria, acute post-thoracotomy pain and development of chronicity in pain with benign thoracotomy operations. *Turk Gogus Kalp Dama* 2014; 22(2): 389-96.

**Koyuncu 2018**

Koyuncu O, Hakimoglu S, Ugur M, Akkurt C, Turhanoglu S, Sessler D, Turan A. Acetaminophen reduces acute and persistent incisional pain after hysterectomy. *Annali Italiani di Chirurgia* 2018; 89: 357-66.

**Lakdja 1997**

Lakdja F, Dixmerias F, Bussieres E, Fonrouge JM, Lobera A. Preventive analgesic effect of intraoperative administration of ibuprofen-arginine on postmastectomy pain syndrome. *Bulletin du Cancer* 1997; 84(3): 259-63.

**Lee 2017** (*unpublished*)

Lee M, Chung W, Lee J, Kim H. The combination effect of propacetamol with nefopam for postoperative pain after thyroidectomy. *Journal of Pain* 2017; 18(4) (*conference*)

**Lee 2018**

Lee J, Park HP, Jeong MH, Son JD, Kim HC. Efficacy of ketamine for postoperative pain following robotic thyroidectomy: a prospective randomised study. *Journal of International Medical Research*; 46(3): 1109-20.

**Ling 2016**

Ling XM, Fang F, Zhang XG, Ding M, Cang J. Effect of parecoxib combined with thoracic epidural analgesia on pain after thoracotomy. *Journal of Thoracic Disease* 2016; 8(5): 880.

**Lou 2017**

Lou QB, Nan K, Xiang FF, Chen XZ, Zhu WS, Zhang XT, Li J. Effect of perioperative multi-day low dose ketamine infusion on prevention of postmastectomy pain syndrome. *Zhonghua Yi Xue Za Zhi* 2017; 97(46): 3636-41.

**Macheridou 2012** (*unpublished*)

Macheridou A, Giannopoulou A, Karafotia A, Boutsikou M, Kaliviti I, Michaloliakou C. A randomized controlled trial of perioperative pregabalin administration for acute and chronic pain after radical modified mastectomy (RMM). *European Journal of Anaesthesiology* 2012; 29: 198 (*conference*)

**Malek 2006**

Malek J, Kurzova A, Bendova M, Noskova P, Strunova M, Vedral T. The prospective study on the effect of a preemptive long-term postoperative administration of a low-dose ketamine on the incidence of chronic postmastectomy pain. *Anesteziologie a Intenzivni Medicina* 2006; 17(1): 34-7.

**Mao 2020**

Mao Y, Sun X, Si L, Chen L, Liu X, Zhang Z, Gu E. Perioperative dexmedetomidine fails to improve postoperative analgesic consumption and postoperative recovery in patients undergoing lateral thoracotomy for thoracic esophageal cancer: a randomized, double-blind, placebo-controlled trial. *Pain Research and Management* 2020: 4145893.

**Martin 2008**

Martin F, Cherif K, Gentili ME, Enel D, Abe E, Alvarez JC, Mazoit JX, Chauvin M, Bouhassira D, Fletcher D. Lack of impact of intravenous lidocaine on analgesia, functional recovery, and nociceptive pain threshold after total hip arthroplasty. *Anesthesiology* 2008; 109(1): 118-23.

**Martinez 2014**

Martinez V, Cymerman A, Ben Ammar S, Fiaud JF, Rapon C, Poindessous F, Judet T, Chauvin M, Bouhassira D, Sessler D, Mazoit X. The analgesic efficiency of combined pregabalin and ketamine for total hip arthroplasty: a randomised, double‐blind, controlled study. *Anaesthesia* 2014; 69(1): 46-52.

**Matsutani 2015**

Matsutani N, Dejima H, Takahashi Y, Kawamura M. Pregabalin reduces post-surgical pain after thoracotomy: a prospective, randomized, controlled trial. *Surgery Today* 2015; 45(11): 1411-6.

**Mendola 2012**

Mendola C, Cammarota G, Netto R, Cecci G, Pisterna A, Ferrante D, Casadio C, Della Corte F. S (+)-ketamine for control of perioperative pain and prevention of post thoracotomy pain syndrome: a randomized, double-blind study. *Minerva Anestesiologica* 2012; 78(7): 757-66.

**Momon 2019**

Momon A, Verdier B, Dolomie JO, Gardette M, Pereira B, Curt I, Dualé C. A single preoperative administration of dexamethasone, low-dose pregabalin, or a combination of the 2, in spinal surgery, does not provide a better analgesia than a multimodal analgesic protocol alone. *The Clinical Journal of Pain* 2019; 35(7): 594-601.

**Moore 2011**

Moore A, Costello J, Wieczorek P, Shah V, Taddio A, Carvalho JC. Gabapentin improves postcesarean delivery pain management: a randomized, placebo-controlled trial. *Anesthesia & Analgesia* 2011; 112(1): 167-73.

**Myhre 2017**

Myhre M, Romundstad L, Stubhaug A. Pregabalin reduces opioid consumption and hyperalgesia but not pain intensity after laparoscopic donor nephrectomy. *Acta Anaesthesiologica Scandinavica* 2017; 61(10): 1314-24.

**Na 2016**

Na HS, Oh AY, Koo BW, Lim DJ, Ryu JH, Han JW. Preventive analgesic efficacy of nefopam in acute and chronic pain after breast cancer surgery: a prospective, double-blind, and randomized trial. *Medicine* 2016; 95(20): e3705.

**Nielsen 2015**

Nielsen RV, Siegel H, Fomsgaard JS, Andersen JD, Martusevicius R, Mathiesen O, Dahl JB. Preoperative dexamethasone reduces acute but not sustained pain after lumbar disk surgery: a randomized, blinded, placebo-controlled trial. *Pain* 2015; 156(12): 2538-44.

**Nielsen 2016**

Nielsen RV, Fomsgaard J, Mathiesen O, Dahl JB. The effect of preoperative dexamethasone on pain 1 year after lumbar disc surgery: a follow-up study. *BMC Anesthesiology* 2016; 16(1): 1-9.

**Nielsen 2017**

Nielsen RV, Fomsgaard JS, Siegel H, Martusevicius R, Nikolajsen L, Dahl JB, Mathiesen O. Intraoperative ketamine reduces immediate postoperative opioid consumption after spinal fusion surgery in chronic pain patients with opioid dependency: a randomized, blinded trial. *Pain* 2017; 158(3): 463-70.

**Nielsen 2019**

Nielsen RV, Fomsgaard JS, Nikolajsen L, Dahl JB, Mathiesen O. Intraoperative S‐ketamine for the reduction of opioid consumption and pain one year after spine surgery: A randomized clinical trial of opioid‐dependent patients. *European Journal of Pain* 2019; 23(3): 455-60.

**Nikolajsen 2006**

Nikolajsen L, Finnerup NB, Kramp S, Vimtrup AS, Keller J, Jensen TS. A randomized study of the effects of gabapentin on postamputation pain. *Anesthesiology* 2006; 105(5): 1008-15.

**Pavelescu 2009** (*unpublished*)

Pavelescu D, Pavelescu C, Grintescu I. Pregabalin vs paracetamol in multimodal postoperative analgesia for neoplastic and traumatic thoracic surgery. Which is the best? *Euroanaesthesia* 2009 (*conference abstract*).

**Pavelescu 2020** (*unpublished*)

Pavelescu D, Grintescu I. Multimodal analgesia with low dose of ketamine and dexamethasone, an interesting option for patients with total hip arthroplasty? *Critical Care* 2020; 24 (*conference abstract*).

**Pehlivan 2019**

Pehlivan SS, Ülgey A, Bayram A, Biçer C, Oğuzkaya F, Boyaci A. The effect of low dose ketamine infusion on postoperative acute and chronic pain after thoracotomy. *Dicle Tıp Dergisi* 2019; 46(4): 677-84.

**Perrin 2009**

Perrin SB, Purcell AN. Intraoperative ketamine may influence persistent pain following knee arthroplasty under combined general and spinal anaesthesia: a pilot study. *Anaesthesia and Intensive Care* 2009; 37(2): 248-53.

**Pesonen 2011**

Pesonen A, Suojaranta-Ylinen R, Hammaren E, Kontinen VK, Raivio P, Tarkkila P, Rosenberg PH. Pregabalin has an opioid-sparing effect in elderly patients after cardiac surgery: a randomized placebo-controlled trial. *British Journal of Anaesthesia* 2011; 106(6): 873-81.

**Petersen 2018**

Petersen KK, Lunn TH, Husted H, Hansen LT, Simonsen O, Laursen MB, Kehlet H, Arendt-Nielsen L. The influence of pre-and perioperative administration of gabapentin on pain 3–4 years after total knee arthroplasty. *Scandinavian Journal of Pain* 2018; 18(2): 237-45.

**Peyton 2017**

Peyton PJ, Wu C, Jacobson T, Hogg M, Zia F, Leslie K. The effect of a perioperative ketamine infusion on the incidence of chronic postsurgical pain-a pilot study. *Anaesthesia and Intensive Care* 2017; 45(4): 459-65.

**Quail 2017**

Quail J, Spence D, Hannon M. Perioperative gabapentin improves patient-centered outcomes after inguinal hernia repair. *Military Medicine* 2017; 182(11-12): e2052-5.

**Rekatsina 2019** (*unpublished*)

Rekatsina M, Theodosopoulou P, Staikou C. Perioperative administration of lidocaine and dexmedetomidine for the prevention of chronic postoperative pain. *Regional Anesthesia and Pain Medicine* 2019; 44(10).

**Remerand 2009**

Remérand F, Le Tendre C, Baud A, Couvret C, Pourrat X, Favard L, Laffon M, Fusciardi J. The early and delayed analgesic effects of ketamine after total hip arthroplasty: a prospective, randomized, controlled, double-blind study. *Anesthesia & Analgesia* 2009; 109(6): 1963-71.

**Reyad 2019**

Reyad RM, Omran AF, Abbas DN, Kamel MA, Shaker EH, Tharwat J, Reyad EM, Hashem T. The possible preventive role of pregabalin in postmastectomy pain syndrome: a double-blinded randomized controlled trial. *Journal of Pain and Symptom Management* 2019; 57(1): 1-9.

**Romundstad 2006**

Romundstad L, Breivik H, Roald H, Skolleborg K, Romundstad PR, Stubhaug A. Chronic pain and sensory changes after augmentation mammoplasty: long term effects of preincisional administration of methylprednisolone. *Pain* 2006; 124(1-2): 92-9.

**Sadatsune 2016**

Sadatsune EJ, Leal PD, Cossetti RJ, Sakata RK. Efeito da gabapentina pré-operatória na intensidade da dor e desenvolvimento de dor crônica após o tratamento cirúrgico da síndrome do túnel do carpo em mulheres: estudo randomizado duplo-cego controlado com placebo. *Sao Paulo Medical Journal* 2016; 134(4): 285-91.

**Sen 2009a**

Sen H, Szlan A, Yanarates Ö, Senol MG, Inangil G, Sücüllü I, Özkan S, Dagl G. The effects of gabapentin on acute and chronic pain after inguinal herniorrhaphy. *European Journal of Anaesthesiology* 2009; 26(9): 772-6.

**Sen 2009b**

Sen H, Sizlan A, Yanarates O, Emirkadi H, Ozkan S, Dagli G, Turan A. A comparison of gabapentin and ketamine in acute and chronic pain after hysterectomy. *Anesthesia & Analgesia* 2009; 109(5): 1645-50.

**Sessler 2020**

Sessler DI, Conen D, Leslie K, Yusuf S, Popova E, Graham M, Kurz A, Villar JC, Mrkobrada M, Sigamani A, Biccard BM. One-year results of a factorial randomized trial of aspirin versus placebo and clonidine versus placebo in patients having noncardiac surgery. *Anesthesiology* 2020; 132(4): 692-701.

**Shanthanna 2020**

Shanthanna H, Turan A, Vincent J, Saab R, Shargall Y, O’Hare T, Davis K, Fonguh S, Balasubramaniam K, Paul J, Gilron I. N-Methyl-D-aspartate antagonists and steroids for the prevention of persisting post-surgical pain after thoracoscopic surgeries: a randomized controlled, factorial design, international, multicenter pilot trial. *Journal of Pain Research* 2020; 13: 377.

**Shimony 2016**

Shimony N, Amit U, Minz B, Grossman R, Dany MA, Gonen L, Kandov K, Ram Z, Weinbroum AA. Perioperative pregabalin for reducing pain, analgesic consumption, and anxiety and enhancing sleep quality in elective neurosurgical patients: a prospective, randomized, double-blind, and controlled clinical study. *Journal of Neurosurgery* 2016; 125(6): 1513-22.

**Short 2012**

Short J, Downey K, Bernstein P, Shah V, Carvalho JC. A single preoperative dose of gabapentin does not improve postcesarean delivery pain management: a randomized, double-blind, placebo-controlled dose-finding trial. *Anesthesia & Analgesia* 2012; 115(6): 1336-42.

**Sidiropoulou 2016**

Sidiropoulou T, Giavasopoulos E, Kostopanagiotou G, Vafeiadou M, Lioulias A, Stamatakis E, Matsota P. Perioperative pregabalin for postoperative pain relief after thoracotomy. *Journal of Anesthesia and Surgery* 2016; 3: 1-6.

**Singla 2015**

Singla NK, Chelly JE, Lionberger DR, Gimbel J, Sanin L, Sporn J, Yang R, Cheung R, Knapp L, Parsons B. Pregabalin for the treatment of postoperative pain: results from three controlled trials using different surgical models. *Journal of Pain Research* 2015; 8: 9.

**Spreng 2010**

Spreng UJ, Dahl V, Ræder J. Effects of perioperative S (+) ketamine infusion added to multimodal analgesia in patients undergoing ambulatory haemorrhoidectomy. *Scandinavian Journal of Pain* 2010; 1(2): 100-5.

**Sun 2013**

Sun M, Liao Q, Wen L, Yan X, Zhang F, Ouyang W. Effect of perioperative intravenous flurbiprofen axetil on chronic postmastectomy pain. *Journal of Central South University Medical Sciences* 2013; 38(7): 653-60.

**Suppa 2012**

Suppa E, Valente A, Catarci S, Zanfini BA, Draisci G. A study of low-dose S-ketamine infusion as "preventive" pain treatment for cesarean section with spinal anesthesia: benefits and side effects. *Minerva Anestesiologica* 2012; 78(7): 774.

**Suzuki 2006**

Suzuki M, Haraguti S, Sugimoto K, Kikutani T, Shimada Y, Sakamoto A. Low-dose intravenous ketamine potentiates epidural analgesia after thoracotomy. *Anesthesiology* 2006; 105(1): 111-9.

**Sveticic 2008**

Sveticic G, Farzanegan F, Zmoos P, Zmoos S, Eichenberger U, Curatolo M. Is the combination of morphine with ketamine better than morphine alone for postoperative intravenous patient-controlled analgesia? *Anesthesia & Analgesia* 2008; 106(1): 287-93.

**Tena 2014**

Tena B, Gomar C, Rios J. Perioperative epidural or intravenous ketamine does not improve the effectiveness of thoracic epidural analgesia for acute and chronic pain after thoracotomy. *The Clinical Journal of Pain* 2014; 30(6): 490-500.

**Terkawi 2015**

Terkawi AS, Sharma S, Durieux ME, Thammishetti S, Brenin D, Tiouririne M. Perioperative lidocaine infusion reduces the incidence of post-mastectomy chronic pain: a double-blind, placebo-controlled randomized trial. *Pain Physician* 2015; 18(2): e139.

**Toner 2021**

Toner AJ, Bailey MA, Schug SA, Corcoran TB. A pilot multicentre randomised controlled trial of lidocaine infusion in women undergoing breast cancer surgery. *Anaesthesia* 2021; 76(10): 1326-41.

**Turan 2015**

Turan A, Belley-Cote EP, Vincent J, Sessler DI, Devereaux PJ, Yusuf S, van Oostveen R, Cordova G, Yared JP, Yu H, Legare JF. Methylprednisolone does not reduce persistent pain after cardiac surgery. *Anesthesiology* 2015; 123(6): 1404-10.

**Turan 2017**

Turan A, Karimi N, Zimmerman NM, Mick SL, Sessler DI, Mamoun N. Intravenous acetaminophen does not decrease persistent surgical pain after cardiac surgery. *Journal of Cardiothoracic and Vascular Anesthesia* 2017; 31(6): 2058-64.

**Ucak 2011**

Ucak A, Onan B, Sen H, Selcuk I, Turan A, Yilmaz AT. The effects of gabapentin on acute and chronic postoperative pain after coronary artery bypass graft surgery. *Journal of Cardiothoracic and Vascular Anesthesia* 2011; 25(5): 824-9.

**Vasilev 2019**

Vasilev YI, Marova NG, Karelov AE, Grib PA, Timofeev NA. Intraoperative intravenous lidocaine for prevention of chronic pain syndrome. *Annals of Critical Care* 2019; 2: 92-97.

**van Helmond 2016**

van Helmond N, Steegers MA, Filippini-de Moor GP, Vissers KC, Wilder-Smith OH. Hyperalgesia and persistent pain after breast cancer surgery: a prospective randomized controlled trial with perioperative COX-2 inhibition. *PLOS One* 2016; 11(12): e0166601.

**Vig 2019**

Vig S, Kumar V, Deo S, Bhan S, Mishra S, Bhatnagar S. Effect of perioperative pregabalin on incidence of chronic postmastectomy pain syndrome: a prospective randomized placebo-controlled pilot study. *Indian Journal of Palliative Care* 2019; 25(4): 508.

**Weis 2006**

Weis F, Kilger E, Roozendaal B, Dominique JF, Lamm P, Schmidt M, Schmölz M, Briegel J, Schelling G. Stress doses of hydrocortisone reduce chronic stress symptoms and improve health-related quality of life in high-risk patients after cardiac surgery: a randomized study. *The Journal of Thoracic and Cardiovascular Surgery* 2006; 131(2): 277-82.

**YaDeau 2015**

YaDeau JT, Lin Y, Mayman DJ, Goytizolo EA, Alexiades MM, Padgett DE, Kahn RL, Jules-Elysee KM, Ranawat AS, Bhagat DD, Fields KG. Pregabalin and pain after total knee arthroplasty: a double-blind, randomized, placebo-controlled, multidose trial. *British Journal of Anaesthesia* 2015; 115(2): 285-93.

**Zarei 2016**

Zarei M, Najafi A, Mansouri P, Sadeghi-Yazdankhah S, Saberi H, Moradi M, Farzan M. Management of postoperative pain after lumbar surgery—pregabalin for one day and 14 days—a randomized, triple-blinded, placebo-controlled study. *Clinical Neurology and Neurosurgery* 2016; 151: 37-42.
